# Non-Māori-speaking New Zealanders have a Māori proto-lexicon

**DOI:** 10.1038/s41598-020-78810-4

**Published:** 2020-12-18

**Authors:** Y. Oh, S. Todd, C. Beckner, J. Hay, J. King, J. Needle

**Affiliations:** 1 New Zealand Institute of Language, Brain and Behaviour, NZILBB, University of Canterbury, Private Bag 4800, Christchurch, 8140 New Zealand; 2 Department of French Language and Literature, Ajou University, Suwon, South Korea; 3 Department of Linguistics, University of California, Santa Barbara, USA; 4 Department of Applied Linguistics, University of Warwick, Coventry, UK; 5 Department of Linguistics, University of Canterbury, Christchurch, New Zealand; 6 Aotahi - School of Māori and Indigenous Studies, University of Canterbury, Christchurch, New Zealand

**Keywords:** Psychology, Human behaviour

## Abstract

We investigate implicit vocabulary learning by adults who are exposed to a language in their ambient environment. Most New Zealanders do not speak Māori, yet are exposed to it throughout their lifetime. We show that this exposure leads to a large proto-lexicon – implicit knowledge of the existence of words and sub-word units without any associated meaning. Despite not explicitly knowing many Māori words, non-Māori-speaking New Zealanders are able to access this proto-lexicon to distinguish Māori words from Māori-like nonwords. What's more, they are able to generalize over the proto-lexicon to generate sophisticated phonotactic knowledge, which lets them evaluate the well-formedness of Māori-like nonwords just as well as fluent Māori speakers.

## Introduction

What does it mean to ‘know’ a word? Research on language-learning shows that knowledge can range from being able to use the word well, through to having only a vague sense of its meaning^1^. All speakers are aware of words in their native language that they can confidently identify as being a word, but that they would struggle to provide a definition for. We can say that a speaker might ‘know’ this word, albeit not in the same detail as a word they would use on a daily basis.

While adults learning a language explicitly attend to acquiring new vocabulary, this is something that happens implicitly for infants acquiring their first language. The child’s lexicon begins to be acquired substantially earlier than meaning comes to be associated with these words. The set of words that is known, but for which there is no semantic knowledge, is known as the *proto-lexicon*^2–4^ and is an important step in successful language acquisition by infants.

Do adults who are regularly exposed to another language in their ambient environment also build a proto-lexicon? The literature on second language learning focuses on adults who are actively trying to acquire vocabulary, and identifies the first step in knowing a word to be ‘knowing that you know it’ without knowing the meaning^5^. But how much do adults start to automatically and implicitly build a proto-lexicon from languages in the environment around them? New Zealanders have regular exposure to Māori in their ambient environment, although very few people speak the language well. We conduct experiments with non-Māori-speaking New Zealanders in order to investigate whether they have acquired a Māori proto-lexicon. Our first experiment probes this question directly, by assessing whether non-Māori-speaking New Zealanders can distinguish real words from highly Māori-like nonwords. We show that they can.

Our second experiment investigates knowledge of Māori lexical statistics. The lexicon is a core part of a language, from which much linguistic knowledge about the language is generated. Speakers of a language can identify words that are possible and impossible in that language (e.g. *blick* vs. *mgla* in English), and also words that are more or less likely (*clenk* vs. *sishosh*). They do this by learning the statistical properties of the language’s sound patterns. This ‘phonotactic knowledge’ is understood to be a process of statistical generalization over known words^6,7^. The literature on phonotactic well-formedness focusses on adult speakers of a language, and has shown across many studies that their ratings of how well-formed a nonword appears are highly correlated with the statistical properties of the words in their lexicon, statistics that can be simulated by calculating probabilities over the words in a dictionary of that language. Can non-Māori-speaking New Zealanders generalize over a proto-lexicon to generate well-formedness ratings of this type? In Experiment 2 we show that non-Māori-speaking New Zealanders do indeed have extremely sophisticated phonotactic knowledge, which is best modelled by assuming they possess a large proto-lexicon.

## Background

A large literature examines how infants acquire their early vocabulary^2–4,8–11^. Infants harness statistical learning to spot patterns in the co-occurrences of sounds in the language around them^2–4,8–12^. These patterns can be used to identify the boundaries between words, and to start to acquire a lexicon. The identification of even a small number of words can further facilitate overall segmentation success^13^. In the language acquisition literature, statistical learning is regarded as a powerful mechanism underpinning how humans learn natural languages. It has been shown that the statistical learning mechanism is relatively resilient to aging and stable across the lifespan^14,15^. It is a simple, domain-general mechanism which has been applied in different research areas^12^.

Many experiments conducted in laboratory environments have also demonstrated adults’ ability to generate statistical knowledge regarding artificial languages they are exposed to, and to recognize words that have been embedded in a speech stream^15–17^. Most studies are conducted in a single session, and so it is not clear whether these artificial ‘proto-words’ are retained over a longer period. However, Frank et al.^18^ exposed individuals to an artificial language for 10 h. Three years later, participants retained the ability to recognize high frequency words. Moreover, speakers who have experienced language attrition, and lost the ability to speak or understand a language, still show some evidence of implicit vocabulary retention, and are more able to ‘relearn’ vocabulary items later in life^19–21^. We thus know that adults can form a small proto-lexicon from meaningless running speech, and also that previously meaningful words can remain in an implicit proto-lexicon in later life. It is possible that a very large proto-lexicon could be built by life-time residents of a country who are regularly exposed to a language they do not speak.

Studies on statistical learning have been criticised for lack of ecological validity since artificial languages cannot fully account for the complexity and noise of natural languages, and laboratory settings do not resemble natural learning environments^22,23^. In our study, instead of providing an artificial language learning environment, we investigate the knowledge of a real language among adults who do not speak it but have been exposed to it over years of daily life. Specifically, we investigate the knowledge of Māori among adult New Zealanders.

Māori is the indigenous language of New Zealand. Since European arrival, the language has become endangered, although since becoming an official language in 1987 the government has provided funding and devised strategies to promote and revitalize Māori. Most New Zealanders are exposed to Māori in both its written and oral forms on a regular basis^24^. For instance, all school children are exposed to Māori songs, instructions and colour words. Some words have become part of New Zealand English (for example: *whānau* ‘family’; *kai* ‘food’) and approximately half of all place names are Māori. New Zealanders are also exposed to Māori speeches of greeting which, for example, are used to open meetings and official events.

Despite this exposure, the size of active Māori vocabulary in non-Māori-speakers (NMS) is still small: a multi-choice definition task suggests semantic knowledge of just 70–80 words on average^25^. NMS’ small active Māori vocabulary and their exposure to Māori provides a perfect setting for studying an adult proto-lexicon acquired through ambient exposure.

Because Māori has a small phoneme inventory of ten consonants and five vowels (each with a short and a long counterpart, distinguished orthographically by macrons), there is a transparent correlation between symbol and sound. Consequently, written stimuli provide an appropriate proxy through which we can investigate phonotactic knowledge.

## Experiment 1: word identification task

To probe the potential proto-lexicon of NMS, we conducted an online word identification task containing Māori words with varying frequencies, phonotactic probabilities, and phoneme lengths (ranging from 3 to 12 phonemes), together with Māori-like nonwords that span a similar range of phonotactic probabilities and have the same distribution of lengths. After removing unusable participants, data from 85 adult NMS are available for analysis. Most participants self-reported that they had some very basic knowledge of Māori and some low level of ongoing exposure to Māori.

The full set of stimulus materials consists of 1,000 Māori words and 1,000 Māori-like nonwords. Both the words and nonwords span a range of phonotactic probability scores, to enable us to assess the extent to which participants base their ratings on phonotactic knowledge, though the scores of real words tend to be somewhat higher than those of nonwords. The words span five frequency bins, ranging from highly frequent words to words that are relatively rare, and each word is paired with a nonword that has a similar phonotactic score (i.e. within a fixed range), which is assigned to the same frequency bin for the purposes of comparison. Further details are available in the Detailed Materials and Methods Supplement; for details of the properties of the words and their paired nonwords, see Sect. 2.1, and for details of phonotactic scores, see Sect. 4.2.

Every participant responded to a different subset of these words in a randomized order without pairing real words and nonwords: 300 stimuli containing 30 pairs of words and nonwords, randomly selected from each of the five frequency bins. Participants were asked to rate how confident they were that each item they saw was a Māori word, using a scale ranging from 1 (‘Confident that it is NOT a Māori word’) to 5 (‘Confident that it IS a Māori word’). All experimental protocols in Experiments 1 and 2 were approved by the Human Ethics Committee of the University of Canterbury. All experiments were performed in accordance with relevant guidelines and regulations and informed consent was obtained from all participants.

### Can non-Māori speakers distinguish real words from nonwords in our task?

**Figure 1:**
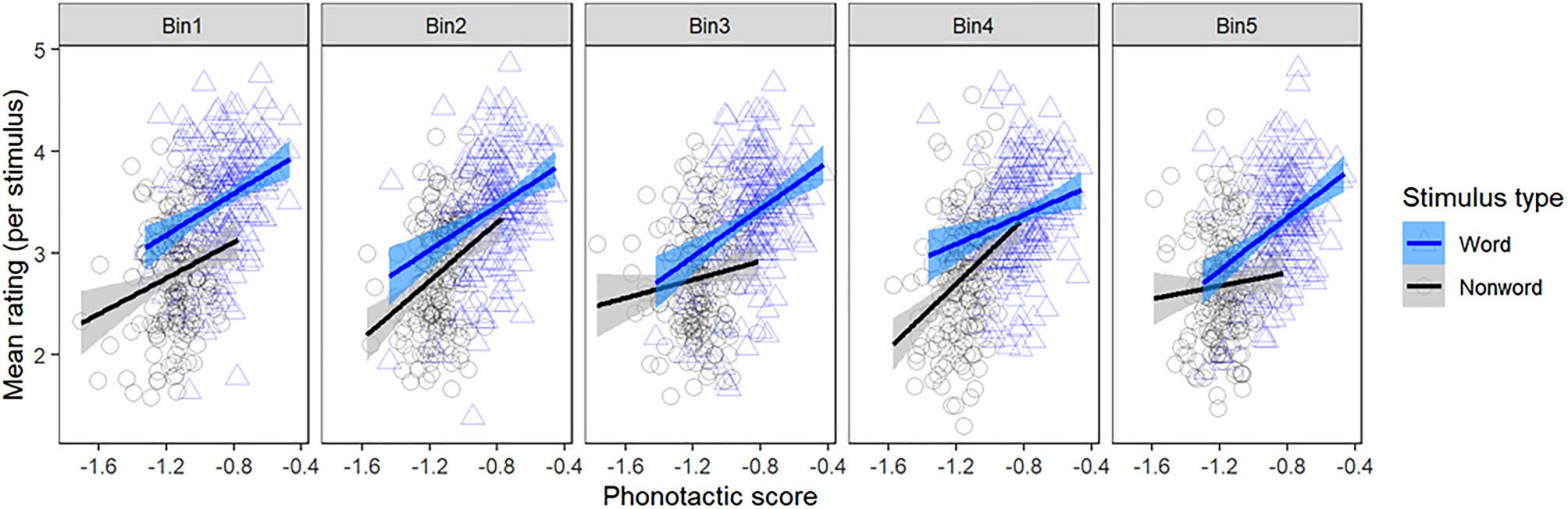
Non-Māori-speakers’ mean wordhood confidence ratings for each stimulus per frequency bin, and their relation to phonotactic score. Bin1 contains the most frequent real words and Bin5 contains the least frequent real words, together with their paired nonwords. Points represent mean ratings for each real word (blue) and nonword (black) within each bin, and straight lines show correlations with phonotactic scores. Across all bins, real words receive higher ratings than non-words, and items with higher phonotactic scores receive higher ratings.

NMS give higher ratings to Māori words than Māori-like nonwords on average per stimulus, as displayed in Fig. 1. This pattern is supported by a significant word/nonword distinction across all five frequency bins in a combined ordinal mixed-effects regression model, which holds even if the analysis is restricted to words and nonwords that are much more closely matched phonotactically (see the Detailed Analysis and Results Supplement, Sects. 2.4–2.5). There is some indication that the distinction is most pronounced for highly frequent words, as demonstrated by a larger separation in Bin1 than in other bins (Fig. 1), but it is also evident even for very low-frequency words. In other words, adult non-Māori speakers can successfully identify many real Māori words, even when they are very low-frequency.

In performing this task, participants show evidence of harnessing phonotactic knowledge: the average rating of an item increases significantly with its phonotactic probability. However, it is important to note that participants’ success at this task cannot be fully explained by knowledge of phonotactic probabilities of Māori. Over and above the effect of phonotactic probability, there is evidence that participants actually know which items are real words and which are nonwords, as reflected in the aforementioned significant main effect of word/nonword distinction in Fig. 1. However, they are not confident in this knowledge. While the ratings of the words and the nonwords are significantly different, they are numerically close together, and participants are not, in general, rating the real words with the top rating of ‘5: Confident that it IS a Māori word’.

The results of Experiment 1 suggest that ambient exposure to a natural language leads adults to develop a proto-lexicon without any active learning. We now investigate the degree to which they can generalize over this proto-lexicon to create phonotactic knowledge of Māori. When presented with nonwords that resemble Māori words to different degrees, are non-Māori-speaking New Zealanders able to rate how Māori-like the words seem? And, if so, how do their ratings compare to those of fluent speakers of Māori?

## Experiment 2: well-formedness rating tasks

In Experiment 2, we conduct a series of online well-formedness rating tasks of Māori-like nonwords. On each trial of this experiment, a participant rated a Māori-like nonword for how good it would be as a real Māori word, using a scale ranging from 1 (‘Non Māori-like non-word’) to 5 (‘Highly Māori-like non-word’). Each participant rated nonwords of a single phoneme length, ranging from 3 to 8. There were 240 or 320 stimuli per length, for a total of 1760 different nonwords overall. Further details about the stimuli are available in the Detailed Materials and Methods Supplement, Sect. 2.2.

Experiment 2 involved three groups of adult participants: non-Māori-speaking New Zealanders (NMS; *N* = 113), fluent Māori-speakers (MS; *N* = 40), and non-Māori-speaking Americans (US; *N* = 94). Most NMS participants self-reported speaking New Zealand English as their first language, though the dataset retains 12 NMS participants who were born outside of New Zealand but have lived there for at least 10 years. Fluent Māori-speakers provide a baseline for task performance given a full lexicon and a lot of phonotactic knowledge, and US speakers for performance given no lexicon and little phonotactic knowledge.

### What type of input predicts the well-formedness ratings best?

We model phonotactic knowledge of Māori by assessing sensitivity to phonotactic probability, calculated over trigrams of phonemes (see the Detailed Materials and Methods Supplement, Sect. 4.2). We expect MS participants to show high sensitivity to phonotactic probability and US participants to show low sensitivity. We predicted that NMS would have an intermediate degree of sensitivity to phonotactic probability, reflecting an intermediate degree of phonotactic knowledge.

We model different potential sources of phonotactic knowledge by using different kinds of data to generate phonotactic probabilities (see the Detailed Materials and Methods Supplement, Sect. 4.1). We compare sources based on their ability to predict the ratings given by each group of participants. For MS, the literature on phonotactics leads us to expect that ratings would be best predicted by statistics generated over a large set of word types in a *dictionary*^6,7^. If NMS are generalizing over a large proto-lexicon, we similarly expect that their ratings should be best predicted by statistics generated over a large set of word types^26^. If, however, they are generalizing over streams of past experience without forming a proto-lexicon, we expect that their ratings should be best predicted by statistics generated over such streams, without necessarily segmenting them into words or coalescing repeated tokens into a single type. We model each type of phonotactic knowledge under different assumptions about vowel length. This is because the literature on the contemporary production of Māori shows that, for the most part, vowel length distinctions are not stably produced^27^*.*

We address the question of where phonotactic knowledge comes from, by comparing the ability of various ordinal mixed-effects regression models to predict participant ratings, across all groups (see the Detailed Analysis and Results Supplement, Sect. 3.3.1). Phonotactics generated over word types in a dictionary are most predictive of ratings, consistent with the idea from Experiment 1 that NMS have access to a large proto-lexicon. The ability to predict ratings is further increased by the assumption that participants do not track vowel length in their phonotactics (see the Detailed Analysis and Results Supplement, Sect. 3.3.2), though they retain a holistic sensitivity to the visual presence of macrons that indicate long vowels.

### Does non-Māori speakers’ phonotactic knowledge differ from fluent Māori speakers?

**Figure 2:**
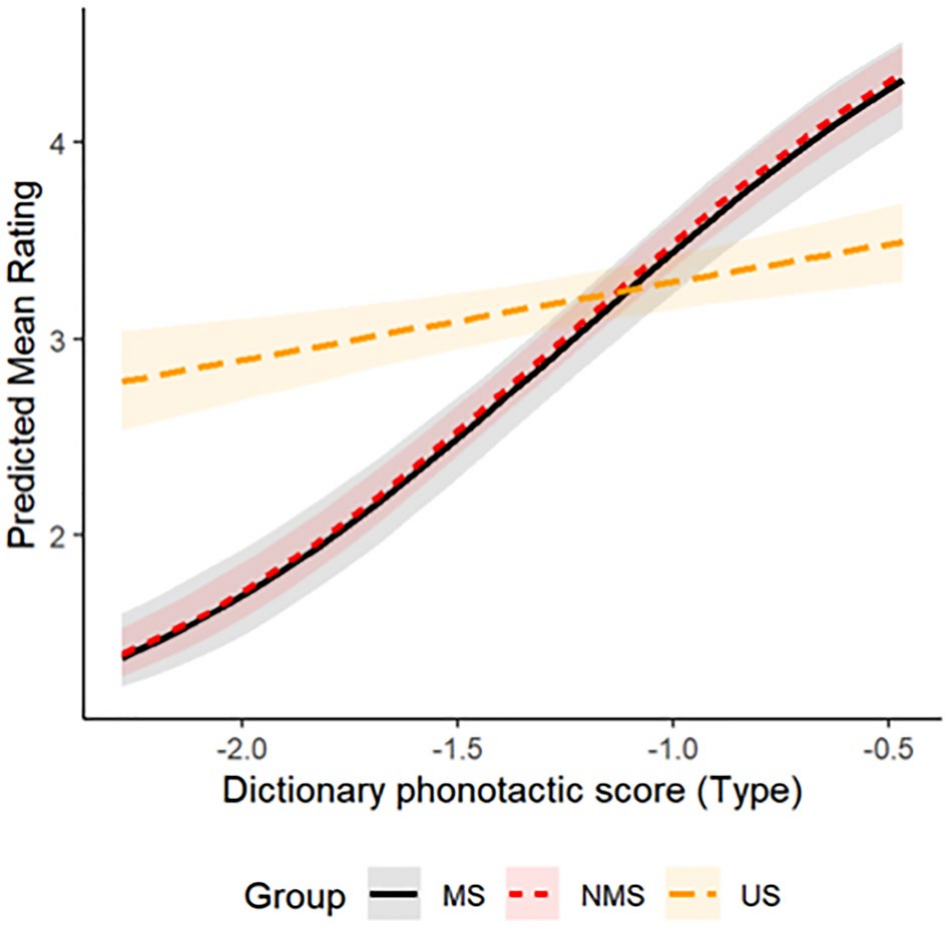
Interaction between dictionary phonotactic score without vowel-length distinction and three groups of participants.

We next address the question of how NMS participants’ sensitivity to phonotactic probability compares to that of MS and US participants. Figure 2 shows a robust interaction from the best-fitting ordinal mixed-effects regression model. As phonotactic probability increases, so do ratings across all participant groups, indicating the use of phonotactic knowledge. The US participants exhibit a relatively small increase in ratings across the phonotactic probability range, indicating minimal phonotactic knowledge that is specific to Māori. By contrast, the NMS and MS participants exhibit a sharp increase in ratings with phonotactic probability, indicating that they have non-negligible phonotactic knowledge of Māori. In fact, the degree of phonotactic knowledge used by NMS in this task does not appear to differ significantly from that used by MS, as shown by the extensive overlap between the red (NMS) and black (MS) lines.

The fact that the US participants show a small sensitivity to Māori phonotactic probability, in spite of most assuredly not having a Māori proto-lexicon, suggests that the proto-lexicon may not be the only source of NMS’ performance in this task. Indeed, it is likely that some amount of phonotactic knowledge may be learned over the course of the experiment, or may be derivable from experience with languages other than Māori or from universal linguistic tendencies. Nevertheless, given the large difference between the sensitivities of the US and NMS participants to phonotactic probabilities, it is apparent that NMS participants must have access to a source of phonotactic knowledge that US participants do not have access to. In other words, while it may not be possible to attribute the entirety of NMS’ performance on this task to the proto-lexicon, we must attribute the majority of it to the proto-lexicon.

### What size does the proto-lexicon need to be?

Taken together, the experimental results so far suggest that ambient exposure has enabled adult NMS to acquire a large proto-lexicon, from which they can generate native-like phonotactic knowledge of Māori. The model that best predicts participants’ ratings in Experiment 2 assumes that their phonotactic knowledge is generated from knowledge of over 18,000 word types (see Sect. 4.1.2 of the Detailed Materials and Methods Supplement for details of these word types). Is it realistic to assume that NMS have a proto-lexicon of this size? Or is the model capturing a generalization that could also be captured by a much smaller vocabulary of types?

To assess the likelihood that NMS’ apparent knowledge of Māori dictionary phonotactics could be captured by small subsets of the Māori lexicon, we next conducted Monte Carlo simulations with varying vocabulary sizes, attempting to predict NMS’ well-formedness ratings (Detailed Analysis and Results Supplement, Sect. 3.3.3). We explored vocabularies between 1,000 and 18,000 words. For each vocabulary size, we repeated the following procedure 1,000 times and collated the results. First, we subsampled the dictionary using one of three different sampling schemes: *unweighted*, which samples types uniformly at random; *frequency-weighted*, which is biased toward sampling types that occur often in corpora; and *N-highest-frequency*, which always samples the types that occur most often in corpora (see the Detailed Materials and Methods Supplement, Sect. 5.3, for details). For present purposes, we focus on the distinction between the unweighted scheme, which assumes that NMS proto-lexicon formation may be independent of experiential statistics, and the frequency-weighted and *N*-highest-frequency schemes, which assume that NMS proto-lexicon formation is guided by experiential statistics. Second, we calculated phonotactic probabilities for the stimuli assuming the subsampled vocabulary as the source of phonotactic knowledge. Third, we used these phonotactic probabilities to predict NMS’ and MS’ ratings respectively, by constructing separate ordinal regression models for each group. Finally, we extracted the AIC score for each model, which represents the amount of error in the model's prediction.

**Figure 3:**
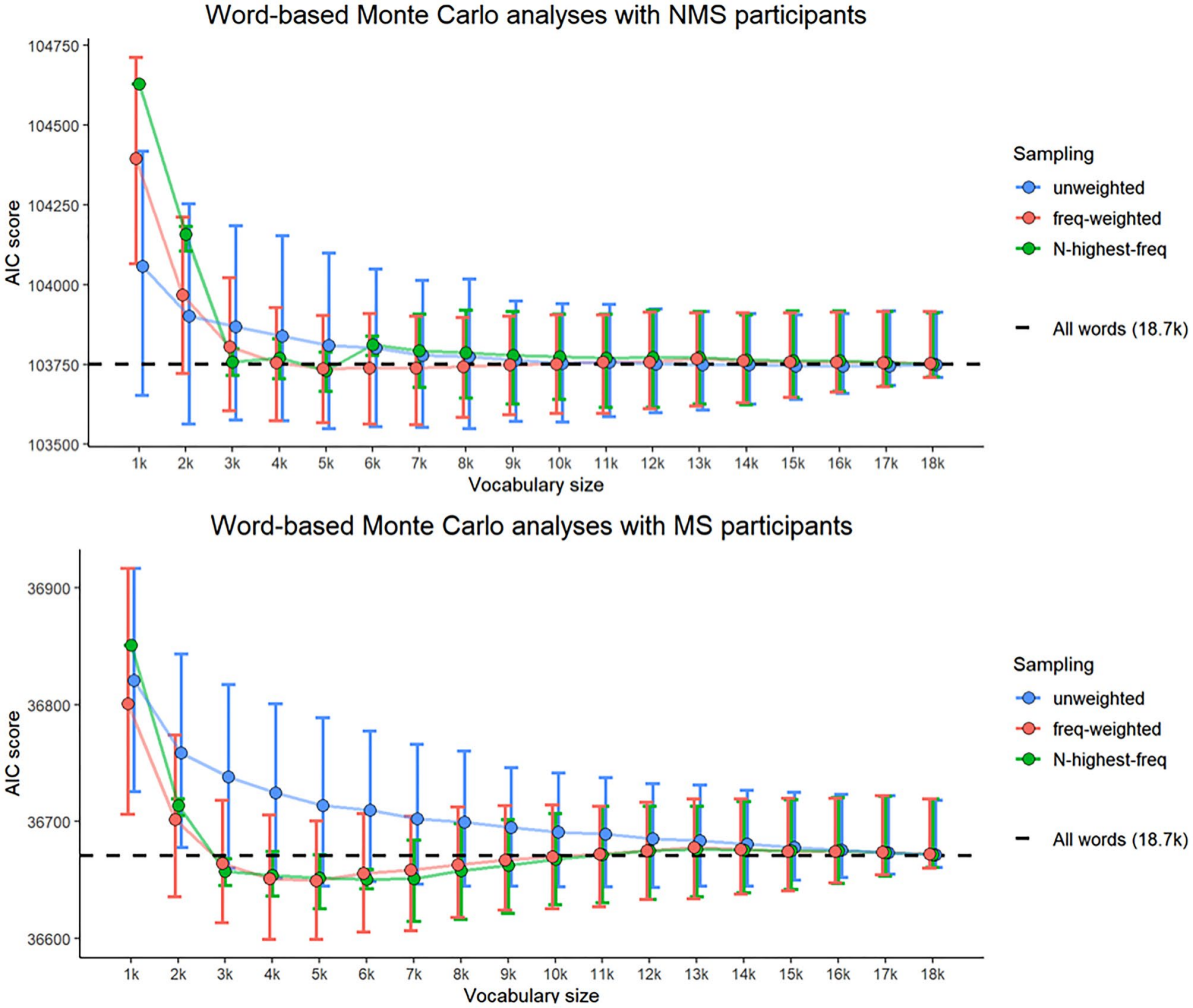
Monte Carlo Simulations with 1,000 samples for each vocabulary size. Lower AIC score indicates greater ability to predict participants’ well-formedness judgements. The black dashed line shows the AIC score of a model that assumes phonotactic knowledge generated from the full dictionary of 18,703 words. The error bars represent 95% bootstrap percentile intervals.

The results of the Monte Carlo simulations are shown in Fig. 3. The AIC score is shown on the *y*-axis; lower values represent sources of phonotactic knowledge that are better able to predict participant ratings. For both groups, a vocabulary of between 3,000 and 6,000 words generates sufficient phonotactic knowledge to predict their ratings at least as well as the full dictionary of over 18,000 words, if not better. However, not any random sample will do: the vocabulary must be sampled in a way that reflects experiential statistics (i.e. word frequency).

The Monte Carlo results show that the well-formedness ratings of NMS participants can be adequately explained by assuming that their phonotactic knowledge is based on a subset of the Māori lexicon. In a follow-up analysis, we found that the subset of Māori words and place names that have been borrowed into New Zealand English does not constitute a sufficient basis for the required phonotactic knowledge (see the Detailed Analysis and Results Supplement, Sect. 3.3.4). In other words, the source of NMS participants' phonotactic knowledge of Māori extends beyond their lexicon of relevant words in New Zealand English, to a proto-lexicon which appears to consist of at least 3,000 words that are common in ambient Māori speech.

### Does the proto-lexicon include morphologically complex words?

The NMS proto-lexicon presumably contains only those phonological sequences that recur with sufficient statistical regularity to be extracted reliably from ambient speech. Given this perspective, it is possible that the NMS proto-lexicon does not consist of words at all, but rather of *morphs*, which are phonological (sub)sequences that recur across different words. Like a morpheme, a morph may correspond to a word or a sub-word part, but unlike a morpheme, a morph need not be identifiable with a particular grammatical function or meaning. Work on native speaker reactions to non-words, for example, shows that people decompose words into morphs without knowledge of the meaning of their subparts^28,29^*.* Morphs therefore represent precisely the kind of unit that would be expected to be learned from passive exposure to ambient speech, and there exist a number of statistical learning algorithms that demonstrate how this process might work^30,31^. Furthermore, morphs can be learnable even with a relatively low level of language exposure, and can provide a foundation for recognizing or analyzing words that have never been experienced before^32,33^*.*

To test the possibility that the NMS proto-lexicon consists of morphs, we had a fluent Māori speaker segment each word in the dictionary into its constituent morphs, which we used to generate phonotactic probabilities (see the Detailed Materials and Methods Supplement, Sect. 4.1.3, for details). Because Māori makes heavy use of compounding, most of the resultant morphs correspond to simplex words, and limiting the analysis to morphs has the primary effect of eliminating a large number of morphologically complex forms. Using ordinal mixed-effects regression models, we compared the morph-based phonotactic probabilities with the word-based ones identified earlier in terms of their ability to predict participants’ well-formedness ratings (see the Detailed Analysis and Results Supplement, Sect. 3.3.5). Participants’ ratings were best predicted by phonotactic knowledge based on morph types, suggesting that the NMS proto-lexicon indeed likely consists of morphs.

We went further to explore how many morphs are required to adequately predict the well-formedness ratings of NMS and MS participants. Similarly to the previous Monte Carlo simulations, we sampled the full set of 3,636 morphs 1,000 times using three different sampling methods (*unweighted, frequency-weighted,* and *N-highest-frequency*), to create sets of morphs ranging in size from 500 to 3,500. For each set of morphs, we calculated phonotactic probabilities for both participant groups in two ways, either assuming that the experimental stimuli were phonotactically *parsed* into morphs by participants, or that they were left *unparsed* (see the Detailed Materials and Methods Supplement, Sects. 4.2.2–4.2.3, for details). We tested the ability of these phonotactic probabilities to predict participant ratings in ordinal regression models.

**Figure 4:**
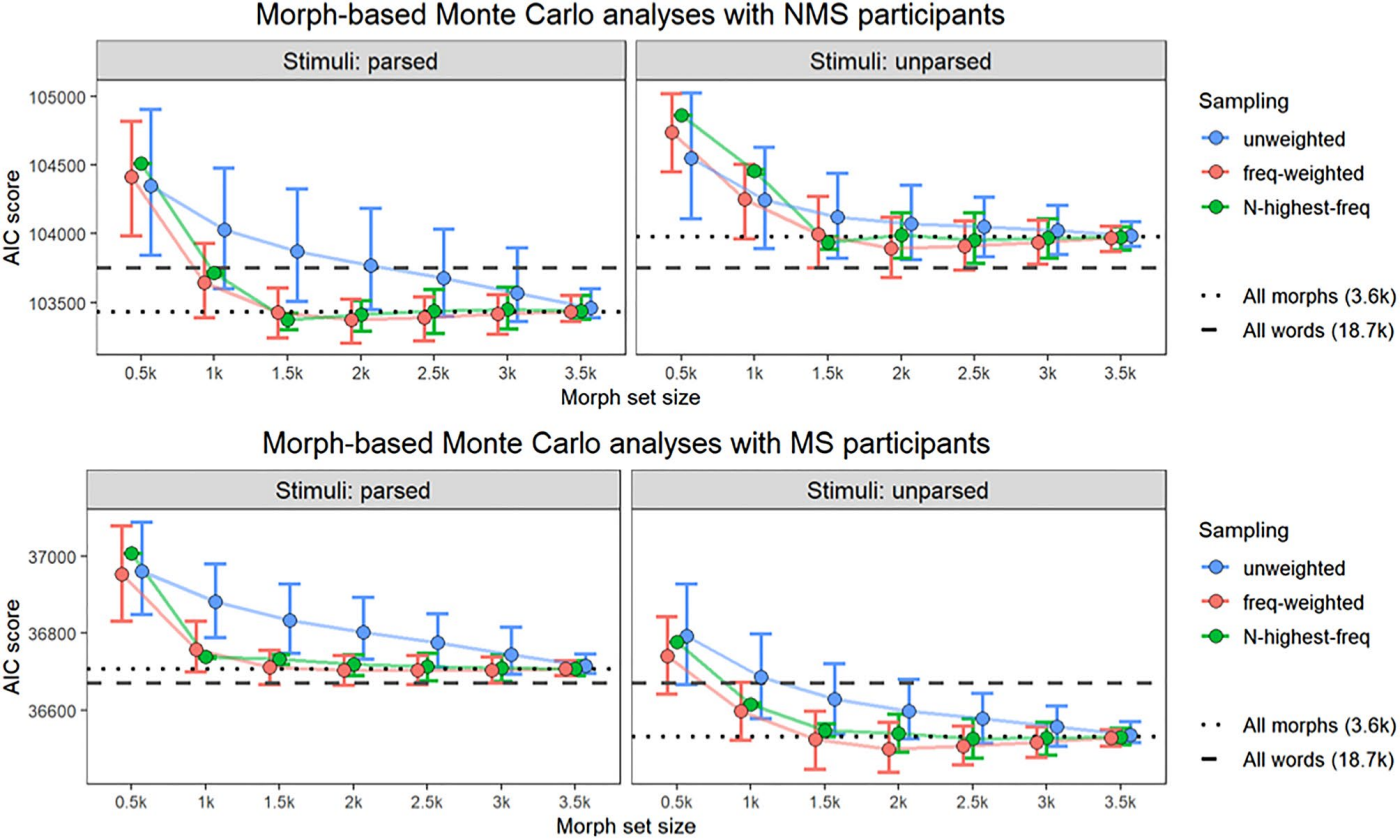
Monte Carlo Simulations with 1,000 samples for each size of morph set. Lower AIC score indicates greater ability to predict participants’ well-formedness ratings. The black dashed line shows the AIC score of a model that assumes phonotactic knowledge generated from the full dictionary of 18,703 words, and the black dotted line shows the AIC score of a model that assumes phonotactic knowledge generated from the full set of 3,636 morphs (assuming that participants are parsing stimuli or not, as appropriate). The error bars represent 95% bootstrap percentile intervals.

Figure 4 shows that the ratings of NMS participants can be adequately predicted by phonotactic knowledge generated over a set of approximately 1,500 of the most common morphs. Follow-up comparison of mixed-effects ordinal regression models confirms that this set of morphs enables better prediction of NMS ratings than the smallest adequate set of words or the set of morphs derived from those words (see the Detailed Analysis and Results Supplement, Sect. 3.3.7). Thus, our analyses suggest that the remarkable phonotactic knowledge of non-Māori-speaking New Zealanders, which appears on par with that of fluent Māori-speakers, is the product of a proto-lexicon consisting of approximately 1,500 of the most common morphs in ambient speech. To get to this size, the NMS proto-lexicon would have to grow at an average rate of 80 or fewer morphs per year (see the Detailed Analysis and Results Supplement, Sect. 3.3.6), which is below the estimated growth rate of the infant proto-lexicon^34^.

Figure 4 also reveals a clear difference between NMS and MS, in the form of whether or not they attempt to parse stimuli into morphs when evaluating them phonotactically. The well-formedness ratings of NMS participants are best predicted if we assume that they attempt to parse the stimuli, while the ratings of MS participants are best predicted if we assume that they do not. We interpret this difference as a reflection of the stimuli used in the task, which were designed according to Māori phonotactics but not according to a grammar of word construction. The NMS participants do not have such a grammar, so they have no reason not to attempt to parse the stimuli. The MS participants do have a grammar of word construction, and can recognize that the stimuli cannot be parsed under that grammar. Thus, the surface similarities between NMS and MS in the effects of phonotactic knowledge (Fig. 2) may mask deeper differences in the way they interact with that knowledge.

### Does reported self-exposure to Māori impact performance?

Finally, to test the effect of participants’ potential exposure background, we added their self-reported exposure to Māori as an additional predictor to the best ordinal mixed-effects model with NMS (see the Detailed Analysis and Results Supplement, Sect. 3.3.8). We observed indications that NMS with more exposure to Māori are more sensitive to phonotactic probability, but the effect was small and did not reach statistical significance. This result affirms that even incidental exposure, repeated over a long period of time, is sufficient to implicitly acquire a substantial proto-lexicon.

## Conclusion

New Zealanders who do not speak Māori have a relatively large Māori proto-lexicon, consisting of more than a thousand phonological sequences that recur with statistical regularity in the language. By statistically generalizing over this proto-lexicon, they can distinguish real words from highly Māori-like nonwords, and they can rate the well-formedness of non-words as accurately as fluent Māori-speakers. This provides a real-world example of the impressive degree to which humans automatically orient to language stimuli in their ambient environment. It is not just infants who possess a ‘proto-lexicon’ of languages in their ambient environment. Adults, too, can possess a large pre-semantic proto-lexicon of a language to which they are regularly exposed.

Without effort or awareness, listeners build a proto-lexicon based on what they hear around them. For many individuals, it may permanently remain a proto-lexicon. For those who are motivated to learn the second language to which they have been exposed, we expect that there is potential for ‘awakening’ the proto-lexicon to more readily attach meanings to the words they already ‘know.’ This should confer them significant advantages over learners who have not accumulated this previous experience.

## Supplementary information

Supplementary Information

Supplementary Information

## Data Availability

All data and materials are available in the provided supplements and the dedicated GitHub repository https://github.com/yoonmioh/SuppInfoProtoLexicon, except the list of words from the *Te Aka* dictionary that were used to train word-based phonotactic models. We are not able to share this list because we purchased it from the publisher for private use; however, we note that an interface to the dictionary is available online at https://maoridictionary.co.nz.
