# Supplementary material for: Non-Māori-speaking New Zealanders have a Māori proto-lexicon
Source: Sci Rep. 2020 Dec 18;10:22318. doi: 10.1038/s41598-020-78810-4 (PMC7749111; doi:10.1038/s41598-020-78810-4)
Supplement: Supplementary file 1 — Supplementary Information [file 41598_2020_78810_MOESM1_ESM.pdf]

Non-Māori-speaking New Zealanders have a Māori proto-lexicon

Detailed Materials and Methods

Authors: Yoon Mi Oh, Simon Todd, Clay Beckner, Jen Hay, Jeanette King, Jeremy Needle

1. Participants

1.1. Experiment 1

In Experiment 1, 100 non-Māori-speaking participants (NMS) were recruited online by Facebook Ads. Participants were based in New Zealand and were 18 years or older. After exclusions, all participants self-reported speaking New Zealand English as their first language.

An overview of participants' demographics is given here; for additional details, see the Detailed Analysis and Results Supplement, Section 2.2. The distributions of participants' age and highest level of education are fairly balanced. There are substantially more female participants than male, and more participants living in the North Island than in the South Island. After removing participants who considered their level of Māori proficiency as at least understanding or speaking 'fairly well', most participants reported that they had some basic knowledge of Māori. Only a few participants responded that their level of exposure to Māori was less than once a year.

15 participants were excluded from the analysis of Experiment 1, leaving 85 participants in total. Reasons for the exclusions were as follows:

- *Failure to recognize borrowings*: Three participants indicated for at least one highly common Māori word borrowed into New Zealand English (*kai*, *haka*, *Aotearoa*) that they were not confident it was a real Māori word.
- *Variety of English*: One participant learned English outside New Zealand and had lived in New Zealand for less than ten years.
- *Māori proficiency*: Two participants had a level of self-reported proficiency of Māori corresponding to at least 'fairly well' (based on their answers to the post-questionnaire).
- *Knowledge of related Polynesian languages*: Two participants had some knowledge of a related Polynesian language.
- *Speech or language impairments*: Five participants reported a history of speech or language impairments.
- *Technical errors*: One participant suffered a technical error which resulted in an incomplete experiment.

- *Uniformity of ratings*: One participant had highly uniform ratings across all items, as indicated by the fact that the standard deviation of their ratings was more than two standard deviations less than the mean standard deviation across all participants.

1.2. Experiment 2

In Experiment 2, there are three groups of participants: fluent Māori speakers (MS), non-Māori-speaking New Zealanders (NMS), and non-Māori-speaking Americans (US). All participants were adults (18 years or older) who self-reported not having previously studied linguistics. MS and NMS were recruited online by Facebook Ads in Māori and English, respectively. US were recruited online from Amazon Mechanical Turk. 40 MS, 151 NMS, and 121 US participants were recruited in total.

Six NMS who self-reported that they could both speak and understand Māori at least 'fairly well' were recategorized as MS, since they met our inclusion criteria for MS participants (though one was later excluded due to technical errors). Note that the instructions and task frames for these MS-by-recategorization participants were in English, whereas they were in Māori for the rest of the MS participants. After this recategorization, we had 46 MS, 145 NMS, and 121 US participants.

After exclusions, most NMS self-reported speaking New Zealand English as their first language, though the dataset retains 12 NMS participants who did not learn their English in New Zealand but have lived there for at least 10 years. After exclusions, all US self-reported speaking American English as their first language.

Detailed information about participants' demographics is presented in the Detailed Analysis and Results Supplement, Section 3.2. There are substantially more female than male participants in MS and NMS; the gender distribution is balanced in US. The distribution of participants' age is also particularly disproportionate in MS and NMS, with more participants in younger groups. There are more high school graduates than undergraduates and graduates in MS, whereas there are more undergraduates in NMS and US. There are more participants from the North Island in both MS and NMS (the North Island is more populous). Most MS self-report being very frequently exposed to Māori in daily life, and only a very small number of MS and NMS self-report being exposed to Māori less than once a year.

6 MS, 32 NMS, and 27 US participants were excluded from the analysis of Experiment 2, leaving 40 MS, 113 NMS, and 94 US in total. Reasons for the exclusions were as follows:

- *Variety of English*
    - 10 NMS participants learned English outside of New Zealand and had lived in New Zealand for less than 10 years.
    - One US participant learned English outside of the United States.

- *Māori proficiency*
    - Two MS participants self-reported that their proficiency level of Māori speaking or understanding corresponded to either 'no more than a few words or phrases' or 'not very well'.
    - 9 NMS participants self-reported that they could either speak or understand Māori at least 'fairly well'. (This does not include the six original-NMS participants who self-reported that they could both speak and understand Māori at least fairly well, and who were reallocated to the MS group prior to exclusions.)
    - 20 US participants reported non-zero Māori proficiency or some basic knowledge of Māori words or phrases.
- *Knowledge of related Polynesian languages*
    - Five NMS participants had lived in Hawaii or had some knowledge of a related Polynesian language.
    - Three US participants had lived in Hawaii.
- *Speech or language impairments*
    - One MS participant reported a history of speech or language impairments.
    - Three NMS participants reported a history of speech or language impairments.
    - One US participant reported a history of speech or language impairments.
- *Technical errors*
    - Two MS participant suffered a technical error which resulted in the wrong number of experimental items (too many for one participant, too few for the other).
    - One US participant suffered a technical error which resulted in them rating too many items.
- *Overly fast responses*
    - One NMS participant had unusually fast responses, as indicated by the fact that their median response time was more than two standard deviations faster than the mean across all NMS participants.

- *Uniformity of ratings*
    - One MS participant had highly uniform ratings across all items, as indicated by the fact that the standard deviation of their ratings was more than two standard deviations less than the mean standard deviation across all MS participants.
    - Four NMS participants had highly uniform ratings across all items, as indicated by the fact that the standard deviation of their ratings was more than two standard deviations less than the mean standard deviation across all NMS participants.
    - One US participant had highly uniform ratings across all items, as indicated by the fact that the standard deviation of their ratings was more than two standard deviations less than the mean standard deviation across all US participants.

2. Stimuli

2.1. Experiment 1

Experiment 1 (identification task) tests NMS' implicit knowledge of Māori words with varying frequencies. Each participant responds to 30 words and 30 matched nonwords from each of 5 bins corresponding to word frequency, for a total of 300 items. These items are randomly sampled from a larger pool of 200 words and 200 matched nonwords per bin (1,000 words and 1,000 nonwords in total).

In addition, each participant responds to three control items that are highly common Māori words borrowed into New Zealand English (*kai*, *haka*, *Aotearoa*). These items were used for participant exclusion (see Section 1.1), but were not otherwise included in the analysis.

The pool of word stimuli was obtained from two corpora of spoken Māori: the Māori Broadcast Corpus (MBC) [*35*] and MAONZE [*36*]. All word tokens in both corpora (approximately 1.35M in total) were counted and combined, and initial bins were formed by splitting words with counts larger than 1 into quintiles by frequency. For ease of interpretation, we have assigned each bin a frequency category following standards in psycholinguistics, based on number of tokens per million words [*37*]. These initial bins were then filtered to exclude: words with fewer than 3 or more than 12 phonemes; proper nouns; words that have been borrowed into New Zealand English; words that have the same orthographic form as an English word; and words that are not listed in the *Te Aka* dictionary [*38*]. From the filtered results, 200 words were randomly sampled for each bin and verified as a real Māori word by a fluent Māori-speaking research assistant. The resulting pools in each bin have the following properties:

- Bin1 (mid-high frequency)
    - *Frequency.* min: 32; max: 14,683; median: 94.5; mean: 574.55; SD: 1856.79
    - *Phoneme length.* min: 3; max: 12; median: 5; mean: 5.11; SD: 1.74

- Bin2 (mid frequency)
    - *Frequency.* min: 10; max: 31; median: 16; mean: 17.54; SD: 6.42
    - *Phoneme length.* min: 3; max: 12; median: 5; mean: 5.2; SD: 1.97

- Bin3 (mid-low frequency)
    - *Frequency.* min: 5; max: 9; median: 6; mean: 6.53; SD: 1.41
    - *Phoneme length.* min: 3; max: 12; median: 5; mean: 5.07; SD: 1.79

- Bin4 (low frequency)
    - *Frequency.* min: 3; max: 4; median: 3; mean: 3.46; SD: 0.50
    - *Phoneme length.* min: 3; max: 12; median: 4; mean: 4.83; SD: 1.88

- Bin5 (low frequency)
    - *Frequency.* min: 2; max: 2; median: 2; mean: 2; SD: 0
    - *Phoneme length.* min: 3; max: 12; median: 4; mean: 4.78; SD: 1.52

The pool of nonword stimuli was generated on the basis of phonotactic statistics calculated across all word types that occur in either the MBC, MAONZE, or *Te Aka*. A triphone-based pseudoword generator [*39*] was used to generate a candidate set of 1,000 nonwords for each phoneme length from 3 to 12. This candidate set was then filtered to remove accidentally-generated real words, as well as nonwords that have the same orthographic form as an English word. After filtering, each word and each nonword was assigned a phonotactic score using the method described in Section 4.2.1, which was used for stimulus selection (note that a different phonotactic score was used during the analysis; see Section 4.2.2). For each real word, the nonword of the same phoneme length that had the most similar phonotactic score was selected (all other generated nonwords were discarded). All word-nonword pairs had phonotactic scores within 0.3 of each other; at worst, this difference in phonotactic score means that one item in the pair could have twice the phonotactic probability of the other.

The list of stimuli used in Experiment 1 can be found in the supplementary file `StimuliExp1.txt`.

2.2. Experiment 2

In Experiment 2, each participant is assigned to one of six conditions representing a fixed phoneme length (from 3 to 8), and all stimuli they observe are nonwords with that phoneme length. This arrangement of conditions mitigates the effect of phoneme length on the results. There is a single set of nonword stimuli for each phoneme length condition. The stimuli sets for lengths 3 and 4 consist of 240 nonwords each, and the stimuli sets for lengths 5, 6, 7, and 8 consist of 320 nonwords each.

As in Experiment 1, the nonword stimuli were generated using a triphone-based pseudoword generator trained on all word types in the MBC, MAONZE, and *Te Aka*. A candidate

pool was generated for each length and filtered to remove accidentally-generated real words, as well as nonwords that have the same orthographic form as an English word. Nonwords were chosen from this candidate pool in order to span a range of phonotactic scores.

The list of stimuli used in Experiment 2 can be found in the supplementary file `StimuliExp2.txt`.

3. Procedure

3.1. Experiment 1

Experiment 1 is an online experiment consisting of three parts: an identification task, a grammaticality task (not reported in this paper), and a post-questionnaire (see Section 6).

During the identification task, participants are shown orthographic forms of each item onscreen, in random order. As each item is presented, participants rate how confident they are that it is a real Māori word. The response is a 5-point scale ranging from 1 ('Confident that it is NOT a Māori word') to 5 ('Confident that it IS a Māori word'). There are no prescribed breaks.

3.2. Experiment 2

Experiment 2 is an online experiment consisting of two parts: a well-formedness rating task and a post-questionnaire (see Section 6).

For the rating task, participants are informed that the items are nonwords. They are shown orthographic forms of each nonword onscreen, in random order. As each nonword is presented, participants rate how good it would be as a Māori word. The response is a 5-point scale ranging from 1 ('Non Māori-like non-word') to 5 ('Highly Māori-like non-word'). There are no prescribed breaks.

4. Phonotactic models

4.1. Training data

Phonotactic models were trained using three primary sources of training data: transcribed spoken corpora, i.e. the Māori Broadcast Corpus (MBC) [*35*] and MAONZE [*36*]; the *Te Aka* dictionary [*38*]; and morphs obtained from decompositions of words in *Te Aka* by a fluent Māori-speaking research assistant. Additional models were trained using a dictionary of common Māori borrowings and placenames in New Zealand English [*40*]. Here, we describe the data preparation steps for the three primary data sources.

4.1.1. Transcribed spoken corpora

For both corpora, we stripped punctuation and removed English words (i.e. words that don't conform to categorical Māori phonotactics), incomplete words, hesitations, and fillers. We split hyphenated compounds into separate component words whenever those components were listed in the *Te Aka* dictionary; when they were not listed, we concatenated the components to form a single word without hyphens. From the resulting set of all tokens in the corpora, we compiled a list of all words not listed in Te Aka, and a fluent Māori-speaking research assistant identified them as nonwords or as mistranscribed words. We removed nonwords and corrected mistranscriptions. For MAONZE, which is composed of sociolinguistic interviews, we removed utterances made by the interviewer, as well as word lists and reading passages.

We concatenated the corpora and prepared two forms of training data: segmented and unsegmented. For the segmented data, we extracted a list of Māori word tokens and their counts. We added to this list all words from *Te Aka* that were not found in either corpus, with a raw token count of 0. This yielded a list containing 24,856 word types. We obtained word frequency estimates by smoothing the raw token counts, using the methodology described in Section 4.1.4.

For the unsegmented data, we did not separate individual word tokens. Instead, we separated the corpora into uninterrupted speech streams, where a new stream was initialized by any of the following: (i) a change of speaker; (ii) an omitted portion of the transcription; (iii) a filler or nonword; (iv) an English word; (v) a long pause; or (vi) a hesitation. We extracted these uninterrupted speech streams as continuous phonological sequences, to use in place of word tokens in training a phonotactic model.

4.1.2. Dictionary

We obtained a list of all headwords listed in *Te Aka*, as well as their inflected (passive) forms. From this list, we removed affixes (headwords that begin or end with a dash) and proper nouns (headwords that begin with a capital letter). The removal of proper nouns was motivated by three primary factors. Firstly, while proper nouns are phonotactically legal, they may not adequately represent the phonotactic statistics of the rest of the lexicon; in our dataset, uncommon (or otherwise-unattested) triphone sequences were overrepresented among proper nouns, perhaps due to a large number of borrowed foreign place names. Secondly, proper nouns may not be constructed following the morphological grammar of the language, which would interfere with our morph-based analyses; for example, the majority of words in Māori involve compounding, reduplication, or affixation, but personal names may be freely constructed by other means. Thirdly, many proper nouns in the dictionary are place names that are used in New Zealand English, and that may therefore not be categorized by non-Māori-speaking New Zealanders as "Māori" in the same way that other words are.

For the remaining words, we stripped punctuation, except hyphens, and replaced digits with spelled-out number words. We split compound words and phrases into their components by

separating on whitespace and hyphens, and we removed proper noun components (components that began with a capital letter). These operations yielded a list containing 19,595 word types. We used the smoothed frequency estimates from the corpora as token frequencies for these word types.

We used the words from the dictionary in three different kinds of phonotactic models, with different treatments of long vowels. Anecdotal evidence suggests that both Māori-speaking and non-Māori-speaking New Zealanders struggle to identify vowel length reliably, and acoustic analyses show that the phonetic distinction between long and short vowels has decreased over time for all vowels except /a ~ a:/ [*36*]. Thus, in addition to the original dictionary training data, in which all long and short vowels are distinct, we developed two modified sets of dictionary-based training data, in which we treated certain long vowels as short. The phonotactic models trained on these sets therefore assume that listeners' phonotactics do not track certain vowel length distinctions. In the first set, we treated all long vowels except /a:/ as short, yielding a total of 19,193 word types. In the second set, we treated all long vowels (including /a:/) as short, yielding a total of 18,703 word types. In both cases, when collapsing the distinction between words differentiated only by vowel length, we combined their token frequencies.

4.1.3. Morphs

In our analysis, we explore the possibility that participants' phonotactic knowledge could be built over units that could potentially be smaller than the word, or *morphs*. To enable this, we asked a fluent Māori-speaking research assistant to decompose words in the *Te Aka* dictionary into parts. We did not ask for a decomposition of all 19,595 words in the dictionary, since some words are clearly simplex or the result of productive and transparent morphological processes.

Māori has been argued to have a strong constraint stating that content words must consist of at least two morae [*41*]. We interpreted this constraint to mean that anything bimoraic or smaller is simplex, i.e. consists of a single morph, and therefore cannot be decomposed. However, Māori also has a productive process of full reduplication, and it has been argued that some monomoraic morphemes undergo such reduplication to meet the minimal word constraint when surfacing on their own (i.e. when not derived, inflected, or compounded). For example, [*42*] argues that the underlying root morpheme of *mimi* is /mi/, since the corresponding passive *mīia* drops one of the instances of /mi/, as is commonly done for cases of reduplication (cf. *tapitapi* > *tāpia*). Thus, we opted to have the research assistant judge the 34 bimoraic words that consist of a repeated syllable, but we held out 1,014 other words that were bimoraic or smaller.

As mentioned above, Māori has a productive process of full reduplication. It also has two productive prefixes, causative *whaka-* and agentive *kai-*, and two productive suffixes, passive *-Cia* and nominalizing *-Canga* (both with numerous allomorphs, where *C* takes on a range of consonantal values). These processes are all transparent: reduplication creates a word that consists of a repeated phoneme sequence, and affixation is anchored at the edge of the word. Thus, it is easy to identify and undo potential instances of them. For present purposes, we assume that all

potential instances of these processes are actual instances of them, and we undo all combinations of them in each word in order to arrive at a set of stem potential stems. We held out all 6,360 words for which any of these potential stems were independently listed in the dictionary. If none of the potential stems for a word were in the dictionary, we retained the word in the list, despite its apparent morphological transparency; a total of 691 words met this criterion.

After these steps, we were left with 12,221 words. We divided these words randomly among 12 sub-lists and put each sub-list in a separate Google Doc, with words in random order and written in a monospaced font. We instructed the fluent Māori-speaking research assistant to work through each list, using their first impression to decompose each word by placing a period (.) between its parts. If they did not know a word, they were to indicate this by placing a slash (/) at the end of the word. We provided a short training session with examples for which the structure is well-known or obvious following our exclusion criteria (*wharenui*: `whare.nui`; *moana*: `moana`; *whakarongo*: `whaka.rongo`; *kaiwhakaatu*: `kai.whaka.atu`). Our instructions intentionally left the criteria for decomposition vague, e.g. by not stating that the words should be decomposed into “meaningful parts”, because we included cases of full and partial reduplication, for which the semantic contribution of the reduplicant is often not clear [*43*]. By the same token, we did not provide any examples involving reduplication, so as not to bias the research assistant toward treating reduplication in a particular way.

Once all 12,221 words were decomposed, we inferred decompositions for the held-out 7,374 words. Under the assumption that the 1,014 held-out short words are simplex, we inferred them to consist of a single morph. For the 6,360 held-out words assumed to be formed from a known stem by productive and transparent morphological processes, we took the decomposed form of the stem and added affixes or reduplicants to it, as necessary, as additional morphs. If the research assistant had indicated that they did not know a stem, we assumed that they also did not know any words formed from that stem. In total, 71 words were labeled as unknown, leaving us with decompositions for 19,524 words.

We extracted a set of all morphs by splitting each of these 19,524 words on periods (or taking the entire word as a morph, if no periods were present). This process yielded a set of 4,263 morphs. For each morph, we obtained a frequency estimate by summing the smoothed frequencies of each word containing it.

In our analysis, we used these morphs to construct a phonotactic model that assumes participants are not tracking long vowels in their phonotactics. To do so, we relabeled all long vowels as short, and we summed the frequency estimates of morphs distinguished only by vowel length. This gave us a total of 3,636 morphs.

The morph set produced by the research assistant undoubtedly relies partly on semantics, to which the NMS of course have no access. Nevertheless, the evaluation of computational tools for morphological segmentation has established that morphemes identified on the basis of

semantics are reasonably correlated with morphs identified via statistical learning without reference to semantics [*44*]. On this basis, we assume that there is a substantial overlap between the morphs that are identified by a native speaker and those that are statistically supported as recurrent over-represented phonological sequences in the NMS's experience.

4.1.4. Frequency smoothing

We used smoothing to obtain token frequency estimates from raw counts in our corpora, so as to better reflect the statistical properties of the Māori lexicon. For each word that was observed in the corpora, smoothing decreases its frequency estimate from the observed count, c, to a smoothed count, s_c, and lets the shortfall of tokens be made up by words that were not observed in the corpora. In this way, smoothing allows each word that was not observed in the corpora to receive a non-zero frequency estimate, which is particularly important because it allows us to account for words in the dictionary that do not appear in the corpora. We applied Simple Good-Turing smoothing [*45*], with a minor modification in the renormalization step, to the raw counts obtained by concatenating the two corpora.[1]

The idea underpinning Good-Turing smoothing is to reallocate the tokens associated with the set of words that occur $c + 1$ times in the corpora to the set of words that occur c times, enabling some tokens to be reserved for words that were not observed. The Simple Good-Turing algorithm presents a practical way to deal with sampling error in this reallocation. The algorithm provides a threshold k such that the number of unique word types that occur c times in the corpora, N_c, is assumed to be accurate for $c \leq k$ (low-frequency words) but affected by sampling error for $c > k$ (all other words). It calculates unnormalized smoothed counts u_c corresponding to each raw count c differently in these two cases:

$$u_c = \begin{cases} (c+1)\dfrac{N_{c+1}}{N_c} & \text{if } c \leq k \\ (c+1)\dfrac{\mathrm{E}[N_{c+1}]}{\mathrm{E}[N_c]} & \text{if } c > k \end{cases}$$

Because the unnormalized smoothed counts are calculated in different ways for different words, the total number of tokens they represent, $U = \sum_c u_c N_c$, is not the same as the original number of tokens in the corpus, $C = \sum_c cN_c$. The renormalization step corrects this mismatch. The

[1] The discussion of the Simple Good-Turing algorithm presented here uses different notation than the original presentation in [*45*], in order to elucidate the effects of renormalization on token frequency estimates. Where the original presentation uses r for raw counts, r^* for corresponding unnormalized smoothed counts, and N for the total token count, we use c, u_c, and C, respectively. Additionally, the original presentation focuses on the derivation of probabilities, p_r, whereas we focus on the derivation of normalized smoothed counts that underpin these probabilities, s_c; the relationship between these values is $p_c = s_c/C$. In the original presentation, the renormalization step consists of rescaling unnormalized probabilities derived from the unnormalized smoothed counts, $p_r^{unnorm} = r^*/N$, into normalized probabilities p_r such that $\sum p_r N_r = 1 - N_1/N$; in our presentation, the renormalization step consists of rescaling the unnormalized smoothed counts into normalized smoothed counts s_c such that $\sum s_c N_c = C - N_1$.

goal of renormalization is to derive the normalized smoothed count corresponding to c, s_c, such that the total number of tokens represented by the normalized smoothed counts, $S = \sum_c s_c N_c$, is equal to $C - N_1$. In this way, a pool of N_1 tokens is reserved to be allocated among unseen words.

In the renormalization step, the standard Simple Good-Turing algorithm enforces proportionality between the normalized smoothed count s_c and the unnormalized smoothed count u_c, setting $s_c = \frac{C-N_1}{U} u_c$. The downside to this approach is that the smoothed normalized counts for the highest-frequency words may be greater than the original counts, which runs counter to the intuition that each word should *decrease* its frequency in order to reserve tokens for the unseen words.

Our modification utilizes an alternative way of performing the renormalization step, which holds proportional the number of tokens each word contributes to unseen words [*46*]. That is, it enforces proportionality between the normalized smoothed tokens that each word contributes to the pool for unseen words, $c - s_c$, and the unnormalized smoothed tokens that each word contributes, $c - u_c$, setting $s_c = c - \frac{N_1}{C-U}(c - u_c)$. This approach ensures that each normalized smoothed count is lower than the original count, since each word makes a positive contribution to the pool of tokens for unseen words in the unnormalized smoothed counts, which can only be scaled in renormalization, not reversed. The result of this alternative method of renormalization is the well-known observation that there is an approximately constant difference between the number of times a word was observed in the corpora, c, and its smoothed frequency, s_c; for example, the word *te* (‘the.SG’), which occurs in the corpora 119,010 times, has a smoothed frequency of 119,009.5, while the word *arawhata* (‘stairs’), which occurs in the corpora 11 times, has a smoothed frequency of 10.52.

One further detail is worth noting, concerning the way we allocated the pool of tokens for unseen words. Good-Turing smoothing reserves N_1 tokens for words that could have been observed in the training data but weren’t, but it makes no assumptions about how many such unseen words there are. In an open-vocabulary setting, one common approach is to assume that all unseen words are instances of a single unknown word type, “UNK”, and all N_1 tokens reserved for unseen words are allocated to this word type. For our purposes, this approach is insufficient, as it does not enable us to separate one unseen word from another: both are “UNK”. Instead, we take an approach that is common to closed-vocabulary settings, where there is a reference wordlist that is assumed to have generated the training data. We calculate the number of unseen words, N_0, as the number of words that occur in the dictionary but not in the corpora; since the corpora include proper nouns, we include proper nouns from the dictionary that do not include in the corpora in this count. We then split the reserved tokens uniformly between these unseen words by setting $s_0 = N_1/N_0$, which means that every unseen word is represented uniquely and thus can be separated from all other unseen words. The downside to this approach is that, since it assumes a closed vocabulary, it provides no coverage of words that do not occur either in the running speech

corpora or in the dictionary. However, since we have no access to the forms of these unknown words, this lack of coverage brings no loss to our analysis, above and beyond the unavoidable loss associated with modeling language knowledge and experience by a particular sample.

A file specifying the c, N_c, u_c, and s_c values obtained from smoothing can be found in the GitHub repository (in `scripts/train/words_corpora_freq-smoothing.txt`). Note that, since our analyses include only non-proper-noun word types from the dictionary and collapses the distinction between words differentiated only by vowel length (see Section 4.1.2), these smoothed frequency values underpin but are not equivalent to the probabilities underlying frequency-weighted sampling in our Monte Carlo analyses (Section 5.3).

4.2. Phonotactic scoring

Different methods of calculating phonotactic scores were used for the selection of stimuli (including the initial matching of nonwords to real words) and for the analysis (including the rematching of nonwords to real words). Additionally, different methods were used for the analysis of Experiment 2 when considering morph-based phonotactic knowledge, depending on whether the participants were assumed to parse the nonword stimuli into morphs or not. We applied these various methods to different sets of training data, as described in Section 4.1, to yield numerous different phonotactic scores.

In all cases, phonotactic scores were calculated using length-normalized log-probabilities from triphone-based n-gram language models trained over word types in the dictionary, as implemented in the SRI Language Modeling Toolkit (SRILM) [*47*]. The use of log-probabilities follows findings in psycholinguistics that they are more predictive of behavior than raw probabilities [*48*] (note that SRILM uses base-10 logarithms for probability calculation). The use of length-normalization enables the statistical comparison of stimuli of different lengths, given that each participant observed stimuli of a single length. The use of triphones allows the model to extend beyond a single syllable and capture known local harmony and disharmony patterns, as well as known differences between word-initial, word-medial, and word-final syllables [*49*]. Each model also used a strategy to score nonwords containing phoneme sequences that are attested in the running speech corpora but not in the dictionary (mostly due to proper names).

4.2.1. Stimulus selection

Though the nonword stimuli were generated with reference to multiple sources, the phonotactic scores that were used to select real words and nonwords as stimuli were calculated solely with reference to the *Te Aka* dictionary. See Section 2 for details of stimulus selection.

For selection purposes, phonotactic scores were computed using a unigram language model, where the unigrams in question were overlapping triphone sequences that included word boundaries (e.g. the word *mana* would consist of the triphones “#ma”, “man”, “ana”, and “na#”); accordingly, the automatic addition of word boundaries by SRILM was disabled. An “UNK” token

was added to account for triphones that were unattested in the dictionary. The phonotactic score for a nonword was computed by dividing its log-probability by the number of triphones required to generate/predict it (equivalently, by the number of phonemes it contained).

This method of computing phonotactic scores has two major drawbacks. Firstly, it does not take account of the conditional dependencies between unigrams, embodied in the fact that they represent partially overlapping triphones. For example, the generation/prediction of "#ma" as the first triphone should place a constraint on the generation/prediction of the second triphone such that it begins with *ma*, but such a constraint is not represented anywhere in the model. By consequence, this scoring method cannot be appropriately extended in any way that relies upon a generative assumption, such as is required for the question of whether stimuli are parsed into morphs (cf. Section 4.2.3). Secondly, it causes all unattested triphones to be treated as "UNK" and given the same probability, though some should intuitively be given vastly lower probabilities than others (e.g. "ēēē" cf. "ingu"). By consequence, nonwords that contain highly unlikely phonotactic sequences are not adequately penalized, meaning that the correlation between phonotactic score and true phonotactic probability breaks down at the lower end of the score scale.

4.2.2. Analysis: assuming stimuli are not parsed into morphs

Given the drawbacks of the phonotactic scoring method used for stimulus selection, we opted to use a different method for calculating phonotactic scores for the analysis. Here, phonotactic scores were generated using a trigram model over phonemes, with Witten-Bell smoothing to account for triphones that were unattested in the training data. The trigram model can be conceptualized as measuring the probability of generating or predicting each phoneme in turn, as well as the end-of-word symbol, given the preceding 2 (or fewer) phonemes as context. Accordingly, the phonotactic score of a stimulus was computed by dividing its log-probability by the number of symbols involved in its generation/prediction: the number of phonemes, plus one (for the end-of-word symbol).

The phonotactic model used in this method does not have the same drawbacks as the unigram model used in the method for stimulus selection. Firstly, since it conditions the generation/prediction of each phoneme on the preceding context, it faithfully represents conditional dependencies. Secondly, its smoothing algorithm allows different unattested triphones to be scored differently, by backing off to consider the probabilities of their attested constituent parts (biphones or phonemes).

We used this method to construct multiple different phonotactic models, based on different training data (see Section 4.1). With the exception of models trained on unsegmented speech streams, these models can be divided into two groups: those based on types, and those based on tokens. The training of both kinds of models utilized lists of unique word or morph types from the relevant training set; in a type-based model, this list was unweighted, while in a token-based model, it was weighted by smoothed frequency. Models trained on unsegmented speech streams utilized

a (non-unique) list of all speech streams in the corpora, unweighted; thus, they were trained analogously to type-based models, though the underlying units are better conceived of as tokens. The SRILM commands used to train the models were as follows (where the types in the input were composed of individual characters representing phonemes, separated by whitespace):

- **Type-based:** `ngram-count -text <INPUT> -lm <OUTPUT> -order 3 -wbdiscount -interpolate`
- **Token-based:** `ngram-count -text <INPUT> -lm <OUTPUT> -order 3 -wbdiscount -interpolate -text-has-weights -float-counts`

Given a phonotactic model, we calculated the log-probabilities underlying phonotactic scores for stimuli by applying the model directly to the generation/prediction of sequences of phonemes in these stimuli. This means that each stimulus is treated as the same kind of entity as makes up the training data: with training data based on unsegmented speech streams, the stimuli are also assumed to be speech streams (i.e. potential multi-word sequences); with training data based on words, the stimuli are also assumed to be words (i.e. with no regard to morphological structure); and with training data based on morphs, the stimuli are also assumed to be morphs (i.e. to be a single morph each, which may be interpreted as corresponding to a morphologically simplex word). When the phonotactic model was trained on data that collapsed certain vowel length distinctions, we also collapsed those distinctions in the stimuli for the purposes of calculating phonotactic probabilities. We used the following SRILM command (where each stimulus was composed of individual characters representing phonemes, separated by whitespace):

```
ngram -lm <MODEL> -ppl <STIMULI> -debug 1 > <OUTPUT>
```

4.2.3. Analysis: assuming stimuli are parsed into morphs

When the training data for the phonotactic model consists of morphs, the method of phonotactic score calculation described in Section 4.2.2 assumes that the stimuli are also morphs (equivalently, are morphologically simplex words). By consequence, participants are assumed not to be parsing the stimuli into sequences of morphs when evaluating them phonotactically. But this is not the only possibility. An alternative assumption would be that participants *are* parsing the stimuli into sequences of morphs, and thus are treating them as potentially complex. Here, we describe a method of calculating phonotactic scores under this alternative assumption.

This method begins from the morph-based phonotactic model described in Section 4.2.2. We modified this model, replacing all instances of the start symbol “<s>” and end symbol “</s>” with a common morph boundary symbol, “+”. Implementationally, when combining the unigrams “<s>” and “</s>”, we took the log-probability associated with “</s>” and the log-backoff-weight associated with “<s>”. We then made additions to the model that rule out the placement of a morph boundary immediately after another morph boundary (“+ +”) or any consonant (“*C* +”, for all

consonants *C*). Implementationally, these additions consisted of adding bigrams with a log-probability of -99.

To calculate log-probabilities underlying phonotactic scores of stimuli, we applied this model with hidden events [*50*], where the only possible hidden event consisted of the insertion of a morph boundary ("+"). As before, when the phonotactic model was trained on data that collapsed certain vowel length distinctions, we also collapsed those distinctions in the stimuli for the purposes of calculating phonotactic probabilities. Since we had modified the language model to unify the start and end symbols ("<s>" and "</s>", respectively) as both corresponding to morph boundaries ("+"), we disabled the automatic generation of start and end symbols in the application of the model and instead added a morph boundary symbol ("+") at the beginning and end of each stimulus to be generated/predicted. We used the following SRILM command (where each stimulus was composed of individual characters representing phonemes, plus an initial and final morph boundary "+", all separated by whitespace, and where the file `morph-boundary.txt` contained only the morph boundary symbol, "+"):

```
ngram -lm <MODEL> -ppl <STIMULI> -debug 2 -hidden-vocab morph-boundary.txt -no-eos -no-sos > <OUTPUT>
```

To calculate the phonotactic score of a stimulus in an analogous manner to the previous methods, we took its log-probability, subtracted the unigram log-probability of the generation/prediction of the initial morph boundary (since it is assumed to be a given start symbol), and divided the result by the length of the stimulus in phonemes, plus one.

The intuition of the language model with hidden events can be understood as follows. A stimulus S can be represented as a sequence of symbols surrounded by morph boundaries, "+ s_1 ... s_n +". The language model is given the starting point, "+", and must evaluate the probability of generating each remaining symbol in the sequence in turn, given a context of (up to) two previous symbols. In doing so, it considers all possible parses of the stimulus into morphs, corresponding to all possible sequences formed by inserting morph boundary symbols between phoneme symbols (the insertion of a morph boundary between a phoneme symbol and the start or end morph boundary is prohibited by the modifications to the language model, so can be ignored). For example, for the stimulus *ani*, four parses are considered, corresponding to the following sequences:

1. + *a n i* +
2. + *a* + *n i* +
3. + *a n* + *i* +
4. + *a* + *n* + *i* +

For each parse, the language model calculates the phonotactic probability of the corresponding sequence; this represents the joint probability of the original sequence of symbols in the stimulus and the sequence of additional "hidden" morph boundaries and non-boundaries

between each pair of original symbols imposed by the parse. Due to the modifications to the language model described earlier, any sequence with a morph boundary after a consonant receives probability 0; for example, parses 3 and 4 above receive probability 0. Furthermore, because the training data consists of isolated morphs, a morph boundary cannot occur in the middle of an n-gram; whenever such an n-gram is observed, the model backs off directly to a lower-order n-gram where the context preceding the morph boundary is erased (implementationally, this occurs because the corresponding backoff weight is $\alpha(+| \ldots) = 1$). For example, the probability of the trigram "$a + n$" is evaluated by considering the probability of the bigram "$+ n$", without any regard to the preceding "a". Thus, the joint probabilities for the sequences above are as follows:

1. $P(a \mid +) \times P(n \mid + a) \times P(i \mid a\ n) \times P(+ \mid n\ i)$
2. $P(a \mid +) \times P(+ \mid + a) \times P(n \mid +) \times P(i \mid + n) \times P(+ \mid n\ i)$
3. 0
4. 0

Since the actual parse is unknown, the phonotactic probability of the stimulus is obtained by marginalizing out the parses, i.e. by summing the phonotactic probability corresponding to each parse.

It is important to note that, even though the stimuli are assumed to be parsed into morphs, all possible parses are considered, including those that include no morphs that were actually attested in the training data. Unattested morphs can still be evaluated on the basis of their phonotactics. In other words, even though the model parses stimuli into morphs, it is a phonotactic model that evaluates an infinity of possible morphs based on their form, rather than a morphotactic model that assumes a finite (fixed) set of morphs.

5. Statistical analysis

5.1. Ordinal regression

The statistical analyses we perform all utilize (logit) ordinal regression, as implemented in the functions `clm` (fixed-effects only) and `clmm` (mixed-effects) in the *R* package `ordinal`. In all regression models using phonotactic scores that ignore vowel length distinctions (see Section 4), we include an additional (binary) predictor for whether or not the stimulus includes at least one long vowel. We make this inclusion because the visual presence of macrons may be highly salient for assessing a word's "Māori-ness" even if participants do not track the length distinctions these macrons indicate in their phonotactic knowledge.

While variants of ordinal regression are gaining increasing recognition as the most appropriate way to analyze responses on a discretized scale [*51*], the use of these methods is still not widespread in linguistics. In this section, we describe the ideas underlying (logit) ordinal regression, to aid understanding of our analysis.

Ordinal regression can be understood in a similar way to (binary) logistic regression. Logistic regression assumes the existence of a latent variable, y^*, that underlies a binary decision, Y. The expected median choice from a sample of equivalent decisions, $\mathrm{M}[Y]$, is assessed by considering whether the latent variable y^* is below or above a threshold at $t_{0|1} = 0$, which represents a tipping point between the "no" (0) option and the "yes" (1) option. If the latent variable is below the threshold, the expected median choice is "no", and if it is above the threshold, the expected median choice is "yes":

$$\mathrm{M}[Y] = \begin{cases} 0 & \text{if } y^* \le t_{0|1} \\ 1 & \text{if } t_{0|1} < y^* \end{cases}$$

Since there are only two options, the expected mode (most likely) choice for any given decision is the same as the expected median choice from a sample of equivalent decisions.

The choice made on any given decision can be obtained by taking the value of the latent variable, adding random noise (in the form of a sample from a logistic distribution with mean 0 and scale 1), and observing whether the result falls above or below the threshold. In this way, the probability of each choice for any given decision can be assessed by comparing the distance between the latent variable y^* and the threshold at $t_{0|1} = 0$, by use of the logistic function $\sigma(z) = 1/(1 + e^{-z})$:

$$P(Y = y) = \begin{cases} 1 - \sigma\left(y^* - t_{0|1}\right) & \text{for } y = 0 \\ \sigma\left(y^* - t_{0|1}\right) & \text{for } y = 1 \end{cases}$$

Ordinal regression assumes that the response Y is not a binary decision, but rather a decision involving the choice of one option among several that are arranged on a scale. It can be conceptualized as a series of logistic regressions all happening at the same time, each of which uses the same latent variable y^* (and therefore the same coefficients) but a different threshold. There is one threshold $t_{i|i+1}$ for each pair of adjacent response options. For expository purposes, we will assume that the scale has response options 1 through 5 (as in the experiments conducted here), yielding 4 thresholds. The expected median choice from a sample of equivalent decisions, $\mathrm{M}[Y]$, is assessed by considering which thresholds the latent variable y^* is below or above:

$$\mathrm{M}[Y] = \begin{cases} 1 & \text{if } y^* \le t_{1|2} \\ 2 & \text{if } t_{1|2} < y^* \le t_{2|3} \\ 3 & \text{if } t_{2|3} < y^* \le t_{3|4} \\ 4 & \text{if } t_{3|4} < y^* \le t_{4|5} \\ 5 & \text{if } t_{4|5} < y^* \end{cases}$$

Provided that the latent variable is not too near any particular threshold, and the thresholds are not too close together, the mode (most likely) choice for any given decision will be the same as the median choice from a sample of equivalent decisions.

The choice made on any given decision can be obtained by taking the value of the latent variable, adding random noise (in the form of a sample from a logistic distribution with mean 0 and scale 1), and observing which thresholds the result falls between. In this way, the probability of each choice for any given decision can be assessed by comparing the distance between the latent variable y^* and the two thresholds corresponding to that choice, by use of the logistic function $\sigma(z) = 1/(1 + e^{-z})$:

$$P(Y = y) = \begin{cases} 1 - \sigma(y^* - t_{1|2}) & \text{for } y = 1 \\ \sigma(y^* - t_{1|2}) - \sigma(y^* - t_{2|3}) & \text{for } y = 2 \\ \sigma(y^* - t_{2|3}) - \sigma(y^* - t_{3|4}) & \text{for } y = 3 \\ \sigma(y^* - t_{3|4}) - \sigma(y^* - t_{4|5}) & \text{for } y = 4 \\ \sigma(y^* - t_{4|5}) & \text{for } y = 5 \end{cases}$$

In our tables reporting ordinal regression results, we include both the estimated coefficients for the predictors (which determine the estimated value of the latent variable y^*, in the manner familiar from other forms of regression) and the estimated thresholds.

In our plots displaying the partial effects of certain predictors in ordinal regression results, we present the expected value of the rating, for ease of interpretation. While this kind of presentation is not technically valid [*51*], as it rests heavily on the assumption of a specific numeric interpretation of the scale of responses, it is highly intuitive. To obtain this rating, we use the equation above to calculate the predicted probability of each response option, using the `effects` package in *R*. We then convert the response options to numerical values 1 through 5 and take an average, weighted by the corresponding predicted probabilities. To obtain the upper (respectively, lower) confidence limits, we take the upper confidence limit of the probability for the highest (lowest) rating, convert it to a latent variable under the assumption that the thresholds are fixed at their maximum-likelihood estimates, and back-compute the probabilities for all other ratings. We note that this method is not technically correct because it doesn't take account of the covariation relationships between thresholds; for this reason, the confidence bands should not be used for inference. Nevertheless, the method does give an indication of the degree of within-group variability; furthermore, it is more accurate than simply using the confidence limits of the latent variable calculated by the `effects` package, as they do not take into account variation in thresholds at all.

5.2. Model comparison with AIC

Our analysis of Experiment 2 consists of a series of comparisons of different ordinal regression models (fitted to the same data), each with different assumptions about participants' underlying phonotactic knowledge. These different assumptions are instantiated through the use of alternative predictors, such as phonotactic scores based on different kinds of representations. Consequently, the models are not nested within each other, and furthermore they all contain multiple predictors (fixed or random effects). As a result, common comparison techniques – such as likelihood-ratio tests, or direct comparison of the correlation of participant well-formedness ratings with phonotactic scores (e.g. via the corresponding regression coefficient or *t*-statistic) – are not appropriate. Instead, we compare the model AIC scores, which is appropriate and fast becoming a recommended standard method [*52*, *53*]. In this comparison, better models have lower AIC.

The AIC score measures the amount of information lost by assuming that the observed data were generated by the model in question [*52*, *53*]; in our case, it can be conceived of as measuring the amount of error involved in the prediction of participant ratings, given particular assumptions about phonotactic knowledge. Because it is based on the model as a whole, it accounts for the ways in which different assumptions about phonotactic knowledge may interact with other factors in the model. For example, when we consider models with phonotactic scores that do not represent long vowels separately from short vowels, we add an extra predictor to retain the possibility that participants are sensitive to the visual presence of macrons indicating vowel length, and the AIC score reflects the joint implications of both predictors. Crucially, the AIC score calibrates for the number of predictors involved in the model, penalizing additional predictors if they do not make sufficient contribution to the model prediction quality; in this may, model comparison via AIC favors models that are both simple and effective.

Because the AIC score represents information loss or prediction error, its absolute value is influenced by the number of datapoints being predicted. For example, in our data, AIC scores for models computed over the large NMS participant group are larger than those for models computed over the smaller MS group. For this reason, the comparison of multiple models draws on *differences* in AIC scores, which can be straightforwardly related to the relative strength of the evidence for various models in probabilistic terms [*52*, *53*]. A standard rule-of-thumb states that a difference of less than 2 AIC points indicates that the models in question have similar degrees of support, while a difference of 10 AIC points between models indicates that the model(s) with higher AIC has essentially no support relative to the model(s) with lower AIC [*52*]. This rule stands regardless of whether the absolute AIC values are small (e.g. 100 vs. 110) or large (e.g. 100,010 vs. 100,000).

5.3. Monte Carlo analyses

At two points in our analysis of Experiment 2, we used Monte Carlo methods to assess whether participants' ratings could be adequately predicted by phonotactic scores computed from a model trained on a subset of types (words or morphs). That is, we (implicitly) performed model selection over phonotactic models with different-sized sets of training data. For NMS, this analysis corresponds to assessing how big the proto-lexicon needs to be in order to adequately derive their behaviorally-observed phonotactic knowledge. We used Monte Carlo methods to compare a range of different proto-lexicon sizes, accounting for the fact that there are many different ways (with varying likelihoods) that one could form a proto-lexicon of a given size.

The basic idea of the Monte Carlo methods is to form a distribution over the performance of ordinal regression models for each proto-lexicon size, where performance is quantified by AIC. For practical reasons of compute time, we used fixed-effects ordinal regression (`clm`) rather than mixed-effects ordinal regression (`clmm`); while this no doubt affected our results quantitatively, we do not expect it to have had a substantial effect qualitatively. In all cases in our analysis, phonotactic scores ignored vowel-length distinctions; accordingly, we included a predictor for the visual presence of macrons in the regression models (see Section 5.1).

For each proto-lexicon size N (e.g. 5,000 words), we performed the following steps 1,000 times:

1. Sample N types from the training data.
2. Train a phonotactic model on these types and calculate phonotactic scores for the stimuli, using the appropriate configuration from Sections 4.2.2-4.2.3.
3. For each participant group, run an ordinal regression predicting participant ratings from phonotactic score and macron presence, and extract the AIC value.

To account for different ways of forming a proto-lexicon of a given size, we used three different sampling schemes, which yield different probability distributions over the set of possible proto-lexicons. Each sampling scheme subsamples N types from the full set of T types, without replacement.

- *Unweighted*: samples types uniformly at random.
  - The probability of the i th sample S_i yielding an as-yet unsampled type t is: $P(S_i = t) = 1/(T - i + 1)$.
  - Under this sampling scheme, all proto-lexicons are equally likely. Intuitively, this means that proto-lexicon formation is not influenced by experiential statistics, i.e. by the frequency with which different units are experienced in ambient exposure.

- *Frequency-weighted*: samples types proportional to their smoothed frequency.
    - The probability of the ith sample S_i yielding an as-yet unsampled type t of frequency $f(t)$ is $P(S_i = t) = f(t)/\sum_{\{x:\, x \notin S\}} f(x)$.
    - Under this sampling scheme, proto-lexicons containing high-frequency types are more likely. Intuitively, this means that proto-lexicon formation is highly sensitive to experiential statistics, in that every experience of a type yields an opportunity to add it to the proto-lexicon. This high degree of sensitivity, coupled with individual-level variation in experiential statistics, may lead different individuals to form substantially different proto-lexicons, containing different low-frequency types.
- *N-highest-frequency*: splits the full set of types into bins, where all types in each bin have the same raw (unsmoothed) frequency. Starting in the highest-frequency bin, samples types uniformly at random until the bin is exhausted or N types have been sampled in total. If the bin is exhausted before sampling N types, moves to the next-highest frequency bin and repeats.
    - This method implies that there is a value k such that the sample includes all types with frequency greater than k, a random subset of types with frequency equal to k, and no types with frequency less than k.
    - Implementationally, we randomly shuffle the list of types, sort it by frequency, and take the top N.
    - Under this sampling scheme, proto-lexicons containing any low-frequency types are impossible, while all proto-lexicons containing only high-frequency types are equally likely. Intuitively, this means that proto-lexicon formation is moderately sensitive to experiential statistics, in that only types that are experienced sufficiently often have an opportunity to be added to the proto-lexicon. This moderate degree of sensitivity provides a measure of counterbalancing against individual-level variation in experiential statistics; while the proto-lexicons formed by different individuals may be different, they will nevertheless be more homogeneous than under the frequency-weighted scheme, as none will contain low-frequency types.

All sampling schemes embody the assumption that participants may have different proto-lexicons, but each scheme places different constraints on the composition of these proto-lexicons. The unweighted sampling scheme places no constraints on composition. The frequency-weighted sampling scheme places loose constraints on composition, whereby the proto-lexicons are biased to reflect experiential statistics of Māori. The *N*-highest-frequency sampling scheme places strong constraints on composition, whereby the proto-lexicons are defined by the experiential statistics of Māori and compositional variation is limited to a small number of types. We include the *N*-highest-frequency sampling scheme so as to indicate the degree to which differences between the

unweighted and frequency-weighted sampling schemes can be attributed to a fixed set of core types of the highest frequency.

We use the insights of the N-highest-frequency sampling scheme to follow up on the results of the Monte Carlo analysis. For a given proto-lexicon size N, each sample under the N-highest-frequency sampling scheme includes a fixed set of M types with frequency greater than some k. From a range of values of N for which the N-highest-frequency sampling scheme yields sufficiently well-performing regression models, we obtain the corresponding range of values of M, and hence a range of fixed proto-lexicons composed strictly of the highest-frequency types. We compare the ability of phonotactic scores based on these proto-lexicons to predict participant ratings, using the AIC of fixed-effects ordinal regression models as above. From this, we identify the best proto-lexicon composed strictly of the highest-frequency types, which we compare against other potential proto-lexicons via AIC-based comparison of mixed-effects ordinal regression models in a final bid to identify the most likely source of participants' phonotactic knowledge.

6. Post-questionnaire

1. How well are you able to speak Māori?
    - ☐ Very well (I can talk about almost anything in Māori)
    - ☐ Well (I can talk about many things in Māori)
    - ☐ Fairly well (I can talk about some things in Māori)
    - ☐ Not very well (I can only talk about simple/basic things in Māori)
    - ☐ No more than a few words or phrases
    - ☐ Not at all
2. How well are you able to understand/read Māori?
    - ☐ Very well (I can understand almost anything said/written in Māori)
    - ☐ Well (I can understand many things said/written in Māori)
    - ☐ Fairly well (I can understand some things said/written in Māori)
    - ☐ Not very well (I can only understand simple/basic things said/written in Māori)
    - ☐ No more than a few words or phrases
    - ☐ Not at all
3. Which age group do you belong to?
    - ☐ 18 - 29
    - ☐ 30 - 39
    - ☐ 40 - 49
    - ☐ 50 - 59
    - ☐ +60

4. Please state your gender:

5. Please state your ethnicity:

6. Your highest education is:
   - ☐ High school
   - ☐ Undergraduate degree
   - ☐ Graduate degree

7. How often do you think you are exposed to the Māori language in your daily life, by means of Māori radio, Māori TV, online media? *(only included for MS and NMS)*
   - ☐ Less than once a year
   - ☐ Less than once a month
   - ☐ Less than once a week
   - ☐ Less than once a day
   - ☐ Multiple times a day

8. How often do you think you are exposed to Māori language in your daily life, in conversation at work, at home, in social settings? *(only included for MS and NMS)*
   - ☐ Less than once a year
   - ☐ Less than once a month
   - ☐ Less than once a week
   - ☐ Less than once a day
   - ☐ Multiple times a day

9. In the past five years, have you had any children living with you who have attended preschool or primary school in New Zealand? *(only included for MS and NMS)*
   - ☐ Yes
   - ☐ No

10. Please tick all boxes that apply.
    - ☐ I can give a mihi in Māori.
    - ☐ I can sing a few songs in Māori.
    - ☐ I can sing the NZ national anthem in Māori.
    - ☐ I know how to say some basic phrases (e.g. My name is..., I'm from...) in Māori.
    - ☐ I know how to say some commands (e.g. Sit down / Come here) in Māori.
    - ☐ I know how to say some greetings in Māori.
    - ☐ I know how to say some numbers in Māori.
    - ☐ I know how to say some body parts in Māori.
    - ☐ I know how to say some colours in Māori.

11. What region of New Zealand do you live in currently? (Please choose 'overseas' if you are living outside of New Zealand.) *(only included for MS and NMS)*
    - ☐ Northland
    - ☐ Auckland
    - ☐ Waikato
    - ☐ Bay of Plenty
    - ☐ Gisborne
    - ☐ Hawke's Bay
    - ☐ Taranaki
    - ☐ Wanganui
    - ☐ Manawatu
    - ☐ Wairarapa
    - ☐ Wellington
    - ☐ Nelson Bays
    - ☐ Marlborough
    - ☐ West Coast
    - ☐ Canterbury
    - ☐ Timaru - Oamaru
    - ☐ Otago
    - ☐ Southland
    - ☐ Overseas

12. How long have you been living there? *(only included for MS and NMS)*

13. Please state your first language (the language you speak/use most of your time).

14. What country were you living in when you first learned this language?

15. Please list any other languages that you can speak fluently:

16. Have you ever lived in Hawaii?
    - ☐ Yes
    - ☐ No

17. Have you ever lived in New Zealand? *(only included for US)*
    - ☐ Yes
    - ☐ No

18. Do you speak/understand any Polynesian languages such as Hawaiian, Tahitian, Sāmoan, or Tongan?
    - ☐ Yes
    - ☐ No
19. If you replied yes to question 18, please state the language you know.
20. Do you have a history of any speech or language impairments that you are aware of?
    - ☐ Yes
    - ☐ No
