# Supplementary material for: Non-Māori-speaking New Zealanders have a Māori proto-lexicon
Source: Sci Rep. 2020 Dec 18;10:22318. doi: 10.1038/s41598-020-78810-4 (PMC7749111; doi:10.1038/s41598-020-78810-4)
Supplement: Supplementary file 2 — Supplementary Information [file 41598_2020_78810_MOESM2_ESM.html]

Non-Māori-speaking New Zealanders have a Māori proto-lexicon


Code 

- Show All Code
- Hide All Code

# Non-Māori-speaking New Zealanders have a Māori proto-lexicon

### Detailed Analysis and Results Supplement

#### Yoon Mi Oh, Simon Todd, Clay Beckner, Jen Hay, Jeanette King, and Jeremy Needle

#### 2020-12-04

```
# Setup -----

library(knitr)
library(tidyverse)
library(ordinal)
library(effects)
library(MASS)
library(kableExtra)
library(egg)

opts_chunk$set(echo=TRUE, message=FALSE, warning=FALSE, fig.show='hold', results='hold')


# Local functions ----

# A function to center variables
c. <- function (x) scale(x, scale = FALSE)

# Custom function to format ordinal regression results in a nice table
clm_table <- function(mod, digits=3, ...) {
  table <- summary(mod)$coefficients %>%
    data.frame() %>%
    rlang::set_names(c("beta", "se", "z", "p")) %>%
    mutate(
      parameter = row.names(.) %>%
        str_replace_all(
          ., 
          str_c("(", str_c(names(attr(mod$terms, "dataClasses"))[attr(mod$terms, "dataClasses") %in% c("factor", "logical")][-1], collapse="|"), ")"), 
          "\\1 = "
        ) %>%
        str_replace_all(
          .,
          "(?<=^|\\:)(?:c\\.\\()([^:]+)(?:\\))(?=$|\\:)",
          "\\1 (centered)"
        ) %>%
        str_replace_all(., fixed(":"), " &times; "),
      significance = case_when(
        p < 0.001 ~ "\\*\\*\\*",
        p < 0.01 ~ "\\*\\*",
        p < 0.05 ~ "\\*",
        p < 0.1 ~ ".",
        TRUE ~ ""
      ),
      is_threshold = parameter %in% names(mod$alpha)
    ) %>%
    mutate_at(c("z", "p", "significance"), ~ ifelse(is_threshold, NA, .)) %>%
    arrange(is_threshold) %>%
    mutate(
      type = c("Effects", rep("", n()-sum(is_threshold)-1), "Thresholds", rep("", sum(is_threshold)-1))
    ) %>%
    dplyr::select(type, parameter, beta, se, z, p, significance) %>%
    mutate(
      p = ifelse(p<0.001, "<0.001", format(round(p, 3), nsmall=3))
    ) %>%
    kable(digits=3, escape=F, col.names=c("", "Parameter", "Estimate", "Std. Error", "$z$", "$p$", ""), align="llrrrrl", ...) %>%
    column_spec(1, bold=TRUE) %>%
    kable_styling()
  return(table)
}
options(knitr.kable.NA = '')

# Custom function to compute summary data for an ordinal regression model, to be plotted. The "type" argument specifies whether the data are for a latent variable plot, a distributional plot, or a mean rating plot. 
clm_plotdat = function(mod, focal.predictors, type="latent", response.coding=NULL, ...) {
  # For latent variable plot, draw straight from Effect
  if (type=="latent") {
    latentdat <-
      Effect(focal.predictors, mod, latent=TRUE, ...) %>%
      as.data.frame() %>%
      dplyr::select(-se) %>%
      rename("pred"=fit, "lci"=lower, "uci"=upper)
    return(latentdat)
  }
  
  # Get distributional data (used for both of other plots)
  distdat <-
    Effect(focal.predictors, mod, ...) %>%
    as_tibble() %>%
    dplyr::select(all_of(focal.predictors), matches("^(?:(?:L|U)\\.)?prob")) %>%
    pivot_longer(
      cols = -all_of(focal.predictors),
      names_to = c(".value", "response"),
      names_pattern = "^((?:(?:L|U)\\.)?prob)\\.(.+)$"      
    ) %>%
    rename("pred"=prob, "lci"=L.prob, "uci"=U.prob) %>%
    mutate(
      response = rep_len(mod$y.levels, n())
    )
  if (type=="dist") return(distdat)
  
  if (type=="mean") {
    # Get threshold values
    thresholds = c(-Inf, as.numeric(mod$alpha), Inf)
    
    # Get response coding
    if (is.null(response.coding)) {
      response.coding = 1:(length(mod$y.levels))
      names(response.coding) = mod$y.levels
    }
    
    # Build latent variable data based on distributional data
    # Note: this is a HACK since drawing latent variable data from Effect doesn't account for threshold variability. The confidence intervals assume fixed thresholds, which obscures patterns in variation of the thresholds; CIs are thus not correct (but are better than what would be obtained from direct transformation of the latent variable from Effect)
    
    # Get distribution-based latent variable data, assuming fixed thresholds
    latentdat <- distdat %>%
      group_by_at(focal.predictors) %>%
      summarise(
        pred = qlogis(1-pred[1]) + thresholds[2],
        lci = qlogis(1-uci[1]) + thresholds[2],
        uci = qlogis(uci[length(uci)]) + thresholds[length(thresholds)-1]
      ) %>%
      as.data.frame()    
    
    # Get probabilities for each response option
    meandat <- latentdat
    for (i in 1:length(mod$y.levels)) {
      meandat[, paste0("(", mod$y.levels[i], ").prob")] = plogis(latentdat[, "pred"] - thresholds[i]) - plogis(latentdat[, "pred"] - thresholds[i+1])
      meandat[, paste0("(", mod$y.levels[i], ").lci")] = plogis(latentdat[, "lci"] - thresholds[i]) - plogis(latentdat[, "lci"] - thresholds[i+1])
      meandat[, paste0("(", mod$y.levels[i], ").uci")] = plogis(latentdat[, "uci"] - thresholds[i]) - plogis(latentdat[, "uci"] - thresholds[i+1])
    }
    meandat <- meandat %>%
      dplyr::select(-pred, -uci, -lci) %>%
      pivot_longer(
        cols = -all_of(focal.predictors),
        names_to = c("response", ".value"),
        names_pattern = "^\\((.+)\\)\\.([^.]+)$"
      ) %>%
      left_join(
        data.frame(response=names(response.coding), code=response.coding, stringsAsFactors=FALSE),
        by = "response"
      ) %>%
      group_by_at(focal.predictors) %>%
      summarise(
        pred = sum(prob * code),
        lci = sum(lci * code),
        uci = sum(uci * code)
      ) %>%
      ungroup()
    return(meandat)
  }
}

# Custom function to get the information about thresholds, in a nice format for plotting
clm_thresholds = function(mod) {
  thresholds <- summary(mod)$coefficients %>%
  as.data.frame() %>%
  rownames_to_column() %>%
  dplyr::select(1:3) %>%
  set_names(c("label", "value", "se")) %>%
  filter(label %in% names(mod$alpha)) %>%
  mutate(
    lci = value - 1.96*se,
    uci = value + 1.96*se
  ) %>%
  dplyr::select(-se)
  return(thresholds)
}

# A function to add phonotactic scores from a file to the dataset
add_scores = function(data, scoreFile, scoreName) {
  scored_data <- data %>%
    left_join(
      read_csv(scoreFile, col_types=cols_only(item=col_character(),
                                              logprob=col_double())
               )%>%
        transmute(
          item = item,
          !!scoreName := logprob / (nchar(item) + 1)
        )
      , by="item"
    )
  return(scored_data)
}

# A function to run shell scripts on Windows or UNIX-based OS
run_shell = function(cmd) {
  if (Sys.info()[['sysname']] == "Windows") {
    return(shell(cmd, flag="", ignore.stdout=TRUE, ignore.stderr=TRUE))
  } else {
    return(system(cmd, ignore.stdout=TRUE, ignore.stderr=TRUE))
  }
}

# A function to highlight the minimum value in a column, for presentation in a kable
# Highlighting is accomplished by coloring red
highlight_min = function(col, digits=1) {
  rounded <- round(col, digits)
  i <- which(rounded==min(rounded))
  highlighted <- cell_spec(format(rounded, nsmall=digits), align="r")
  highlighted[i] <- cell_spec(format(rounded[i], nsmall=digits), align="r", color="red")
  return(highlighted)
}

# A function to display a table of numbers
display_table = function(dat, caption, digits=3, highlight=c()) {
  table <- dat %>%
    mutate_at(highlight, ~ highlight_min(., digits=digits)) %>%
    kable(caption=caption, digits=digits, escape=F) %>%
    kable_styling()
  return(table)
}
```

# 1 Introduction

This R Markdown file contains all the code used for data exclusion, analysis, and plotting. It describes each step of the analysis and includes all statistical results (e.g. model summaries). For further description of materials and methods, see the Detailed Materials and Methods Supplement.

# 2 Experiment 1: Word identification task

In Experiment 1, non-Māori-speaking New Zealanders (NMS) assigned wordhood confidence ratings to words and nonwords with varying frequencies.

We provide an overview of participants’ demographics below; for further details of participants, including exclusion criteria, see Detailed Materials and Methods Section 1.1. For details of the stimuli, see Detailed Materials and Methods Section 2.1. For details of the procedure, see Detailed Materials and Methods Section 3.1.

```
# Load Exp1 data and filter participants

dataExp1 <- read.delim("./data/dataAnonNotFilteredExp1.txt", sep ="\t", header = TRUE)
dataExp1$word = as.character(dataExp1$word)
Encoding(dataExp1$word) = "UTF-8"

# Remove one participant who did 192 items instead of 303
nbResponse <- aggregate(dataExp1$enteredResponse, by=list(dataExp1$workerId),  FUN=length)
rmParticipant1Exp1 <- nbResponse[!nbResponse$x %in% c(303),]$Group.1 # 4c62b407
dataExp1 <- dataExp1[!dataExp1$workerId %in% rmParticipant1Exp1,]
remove(nbResponse, rmParticipant1Exp1)

# Remove three participants who gave ratings lower than 4 to 3 most common Māori words borrowed into New Zealand English
carrots <- dataExp1[dataExp1$word %in% c("haka","kai","aotearoa"),]
carrots <- carrots[as.numeric(carrots$enteredResponse) < 4,]
rmParticipant2Exp1 <- unique(carrots$workerId) # 51aee430 7d4efcd7 15950569
dataExp1 <- dataExp1[!dataExp1$workerId %in% rmParticipant2Exp1,]
remove(carrots, rmParticipant2Exp1)

# Remove ratings for the three words (used to detect outliers)
dataExp1 <- dataExp1[!dataExp1$word %in% c("haka","kai","aotearoa"),]

# Remove two participants whose speakMaori or compMaori is at least (equal to or above) 3
rmParticipant3Exp1 <- unique(dataExp1[dataExp1$speakMaori >= 3 | dataExp1$compMaori >= 3,]$workerId) # b59cb76f 56496f48
dataExp1 <- dataExp1[!dataExp1$workerId %in% rmParticipant3Exp1,]
remove(rmParticipant3Exp1)

# Remove one participant who did not learn their English in NZ and have been living in their current location in NZ for less than ten years (duration == "short")
summaryExp1WorkerId <- unique(dataExp1[,c("workerId","firstLangCountry","place","duration")])
EngNotInNZExp1 <- summaryExp1WorkerId[!summaryExp1WorkerId$firstLangCountry=="NZ",]
rmParticipant4Exp1 <- unique(EngNotInNZExp1[EngNotInNZExp1$duration=="short",]$workerId) # 2fc57ffc
dataExp1 <- dataExp1[!dataExp1$workerId %in% rmParticipant4Exp1,]
remove(summaryExp1WorkerId, EngNotInNZExp1, rmParticipant4Exp1)

# Remove two participants who know any other Polynesian languages
rmParticipant5Exp1 <- unique(dataExp1[dataExp1$anyPolynesian=="Yes",]$workerId) # aa4d5676 2b08d4eb
dataExp1 <- dataExp1[!dataExp1$workerId %in% rmParticipant5Exp1,]
remove(rmParticipant5Exp1)

# Remove five participants with language impairments
rmParticipant6Exp1 <- unique(dataExp1[dataExp1$impairments=="Yes",]$workerId) 
# ae48fa46 b589ecc3 367285b5 6ff6dcb6 bcae3239
dataExp1 <- dataExp1[!dataExp1$workerId %in% rmParticipant6Exp1,]
remove(rmParticipant6Exp1)

# Remove one participant whose pattern of responses (SD) is below 2SD of the mean of all participants
SD <- aggregate(dataExp1$enteredResponse, by=list(dataExp1$workerId), sd)
cut <- mean(SD$x)-2*sd(SD$x)
rmParticipant7Exp1 <- SD[!SD$x > cut,]$Group.1 # d7a9b857
dataExp1 <- dataExp1[!dataExp1$workerId %in% rmParticipant7Exp1,]
remove(SD, cut, rmParticipant7Exp1)

# Check the total number of usable participants for Exp1
# length(unique(dataExp1$workerId)) # 85
```

## 2.1 Dataset structure

The dataset is structured as follows:

- *workerId* is the unique ID for each participant.
- *enteredResponse* is the wordhood confidence rating for each stimulus.
- *reactionTime* is the reaction time for each rating (in seconds).
- *type* is the classification of each stimulus: word (‘real’) or nonword (‘pseudo’).
- *length* is the phoneme length of each stimulus.
- *word* is the stimulus used for the rating.
- *speakMaori* is each participant’s answer to the question how well they can speak Māori (with a scale ranging from 0 to 5).
- *compMaori* is each participant’s answer to the question how well they can understand/read Māori (with a scale ranging from 0 to 5).
- *maoriProf* is the sum of quantified response for speakMaori and compMaori, which refers to the level of each participant’s Māori proficiency (results can range from 0 to 10).
- *age* is the age group that each participant belongs to.
- *gender* is the gender of each participant.
- *ethnicity* is categorized into binary answers, either Māori (M) or non Māori (non M).
- *education* is each participant’s highest level of education.
- *children* is each participant’s answer to the question whether they have had any children who have attended preschool or primary school in New Zealand in the past five years.
- *maoriList* is each participant’s basic knowledge of Māori (with a scale ranging from 0 to 9).
- *place* is each participant’s current place of living (categorized into binary classification, either North or South Island in New Zealand).
- *duration* is each participant’s time duration of living in their current place (categorized into binary classification, long: > 10 years and short: =< 10 years).
- *firstLang* is each participant’s first language.
- *firstLangCountry* is the country where each participant learned their first language.
- *anyOtherLangs* is the information regarding any other languages each participant can speak.
- *hawaii* is the binary response to the question whether participants have lived in Hawaii.
- *anyPolynesian* is the binary response to the question whether participants know any Polynesian such as Hawaiian, Tahitian, Sāmoan, or Tongan.
- *whichPolynesian* is the information regarding participants’ knowledge of any Polynesian languages if they knew any.
- *impairments* is the answer to the question whether participants have a history of any speech or language impairments.
- *maoriExpo* is each participant’s level of exposure to Māori (with a log-like scale ranging from 2 to 10, combining media and conversational exposure).
- *Freq* is the frequency of real word stimulus obtained from Māori running speech corpora (Freq=0 for pseudowords).
- *bin* is the classification of each stimulus according to their frequency of occurence in Māori running speech corpora.
- *score* is the phonotactic score obtained across word types in the dictionary, with vowel length distinctions preserved (following the improved method in the Detailed Methods and Materials, Section 4.2.2, not the original method used for stimulus generation and selection).

## 2.2 Overview of participants’ demographics

Figure S1 summarizes the distribution of participants on demographic and linguistic axes.

```
# Code for Figure S1: Plot participant demographics

# Basic knowledge of Māori
Exp1ML <- unique(dataExp1[,c("maoriList","workerId")]); Exp1ML$maoriList <- as.factor(Exp1ML$maoriList)
Exp1MLT <- as.data.frame(table(Exp1ML$maoriList)); names(Exp1MLT) <- c("maoriList","freq")
FigS1Exp1ML <- ggplot(Exp1ML, aes(x=maoriList,color=maoriList,fill=maoriList)) +
  geom_bar(aes(),size=.1,show.legend=F) + 
  geom_text(data=Exp1MLT, aes(x=maoriList,y=freq,label=freq), color="black", vjust=-0.2,size=3) + 
  labs(x="Basic knowledge of Māori",y="Number of participants") + 
  coord_cartesian(ylim=c(0, 20)) +
  theme_classic() +
  theme(
    panel.background = element_blank()
  )
remove(Exp1ML, Exp1MLT)

# Māori proficiency 
Exp1MP <- unique(dataExp1[,c("maoriProf","workerId")]); Exp1MP$maoriProf <- as.factor(Exp1MP$maoriProf)
Exp1MPT <- as.data.frame(table(Exp1MP$maoriProf)); names(Exp1MPT) <- c("maoriProf","freq")
FigS1Exp1MP <- ggplot(Exp1MP, aes(x=maoriProf, color=maoriProf, fill=maoriProf)) + 
  geom_bar(aes(),size=.1,show.legend=F) + 
  geom_text(data=Exp1MPT, aes(x=maoriProf,y=freq,label=freq), color="black", vjust=-0.2,size=3) + 
  labs(x="Māori proficiency",y="Number of participants") + 
  coord_cartesian(ylim=c(0, 60)) +
  theme_classic() +
  theme(
    panel.background = element_blank()
  )
remove(Exp1MP, Exp1MPT)

# Exposure to Māori
Exp1ME <- unique(dataExp1[,c("maoriExpo","workerId")]); Exp1ME$maoriExpo <- as.factor(Exp1ME$maoriExpo)
Exp1MET <- as.data.frame(table(Exp1ME$maoriExpo)); names(Exp1MET) <- c("maoriExpo","freq")
FigS1Exp1ME <- ggplot(Exp1ME, aes(x=maoriExpo, color=maoriExpo, fill=maoriExpo)) + 
  geom_bar(aes(),size=.1,show.legend=F) + 
  geom_text(data=Exp1MET, aes(x=maoriExpo,y=freq,label=freq), color="black", vjust=-0.2,size=3) +
  labs(x="Māori exposure",y="Number of participants") + 
  coord_cartesian(ylim=c(0, 20)) +
  theme_classic() +
  theme(
    panel.background = element_blank()
  )
remove(Exp1ME, Exp1MET)

# Gender
Exp1Gender <- unique(dataExp1[,c("gender","workerId")])
Exp1GenderT <- as.data.frame(table(Exp1Gender$gender)); names(Exp1GenderT) <- c("gender","freq")
FigS1Exp1Gender <- ggplot(Exp1Gender, aes(x=gender,color=gender,fill=gender)) + 
  geom_bar(aes(),size=.1,show.legend=F) + 
  geom_text(data=Exp1GenderT, aes(x=gender,y=freq,label=freq), color="black", vjust=-0.2,size=3) +
  labs(x="Gender",y="Number of participants") + 
  coord_cartesian(ylim=c(0, 83)) +
  theme_classic() +
  theme(
    panel.background = element_blank()
  )
remove(Exp1Gender, Exp1GenderT)

# Age
Exp1Age <- unique(dataExp1[,c("age","workerId")]); Exp1Age$age <- factor(Exp1Age$age, levels=c("18-29", "30-39", "40-49", "50-59", "+60"), labels=c("18-29", "30-39", "40-49", "50-59", ">60"))
Exp1AgeT <- as.data.frame(table(Exp1Age$age)); names(Exp1AgeT) <- c("age","freq"); Exp1AgeT$age <- factor(Exp1AgeT$age, levels=c("18-29", "30-39", "40-49", "50-59", ">60"))
FigS1Exp1Age <- ggplot(Exp1Age, aes(x=age,color=age,fill=age)) + 
  geom_bar(aes(),size=.1,show.legend=F) + 
  geom_text(data=Exp1AgeT, aes(x=age,y=freq,label=freq), color="black", vjust=-0.2,size=3) + 
  labs(x="Age group",y="Number of participants") + 
  coord_cartesian(ylim=c(0, 23)) +
  theme_classic() +
  theme(
    panel.background = element_blank()
  )
remove(Exp1Age, Exp1AgeT)

# Education
Exp1Education <- unique(dataExp1[,c("education","workerId")]); Exp1Education$education <- factor(Exp1Education$education, levels=c("high","undergraduate","graduate"), labels=c("high school", "undergrad", "graduate"))
Exp1EducationT <- as.data.frame(table(Exp1Education$education)); names(Exp1EducationT) <- c("education","freq"); Exp1EducationT$education <- factor(Exp1EducationT$education, levels=c("high school", "undergrad", "graduate"))
FigS1Exp1Education <- ggplot(Exp1Education, aes(x=education,color=education,fill=education)) + 
  geom_bar(aes(),size=.1,show.legend=F) + 
  geom_text(data=Exp1EducationT, aes(x=education,y=freq,label=freq), color="black", vjust=-0.2,size=3) + 
  labs(x="Highest education",y="Number of participants") + 
  coord_cartesian(ylim=c(0, 35)) + 
  theme_classic() +
  theme(
    panel.background = element_blank()
  )
remove(Exp1Education, Exp1EducationT)

# Place
Exp1Place <- unique(dataExp1[,c("place","workerId")]); Exp1Place$place <- factor(Exp1Place$place, levels=c("north","south"), labels=c("North Island", "South Island"))
Exp1PlaceT <- as.data.frame(table(Exp1Place$place)); names(Exp1PlaceT) <- c("place","freq"); Exp1PlaceT$place <- factor(Exp1PlaceT$place, levels=c("North Island", "South Island"))
FigS1Exp1Place <- ggplot(Exp1Place, aes(x=place, color=place, fill= place)) + 
  geom_bar(aes(),size=.1,show.legend=F) +
  geom_text(data=Exp1PlaceT,aes(x=place,y=freq,label=freq), color="black", vjust=-0.2,size=3) + 
  labs(x = "Place within NZ", y = "Number of participants") + 
  coord_cartesian(ylim=c(0, 65)) +
  theme_classic() +
  theme(
    panel.background = element_blank()
  )
remove(Exp1Place, Exp1PlaceT)

ggarrange(FigS1Exp1ML, FigS1Exp1Gender, FigS1Exp1Place, FigS1Exp1MP, FigS1Exp1Age, FigS1Exp1Education, FigS1Exp1ME, ncol=3, labels=c("a.", "b.", "c.", "d.", "e.", "f.", "g."))
remove(FigS1Exp1ML, FigS1Exp1Gender, FigS1Exp1Place, FigS1Exp1MP, FigS1Exp1Age, FigS1Exp1Education, FigS1Exp1ME)
```

Figure S1: Overview of participants’ demographics in Exp1.

## 2.3 Visualizations of raw data

### 2.3.1 Distributions of ratings across bins and stimulus types

While there is a substantial amount of overlap between the distributions of ratings for words and nonwords, they appear quite distinct, with words receiving higher ratings on average than nonwords. This patterns holds across all frequency bins.

```
# Code for Figure S2: Plot mean ratings per bin, per stimulus type

#Get mean ratings for each stimulus per bin
allBins <- list()
for(i in 1:5){
  Bin <- dataExp1[dataExp1$bin==i,]
  BinMeanRatings <- aggregate(as.numeric(Bin$enteredResponse), by=list(Bin$word, Bin$type), mean)
  names(BinMeanRatings) <- c("word","type","meanRating")
  BinMeanRatings$bin <- paste0("Bin",i)
  allBins[[i]] <- BinMeanRatings
}
remove(i, Bin, BinMeanRatings)

allBins <- do.call(rbind,allBins)
allBins$type = factor(allBins$type)
levels(allBins$type) = c("Nonword", "Word")

# Get grand mean ratings for each stimulus type per bin
binMeans <- allBins %>% 
  group_by(type, bin) %>% 
  summarize(grand_mean = mean(meanRating))

ggplot(allBins,aes(x = bin, y = meanRating, fill = type)) +
    geom_violin(alpha=0.4, position=position_identity()) + 
    geom_point(data=binMeans, aes(y=grand_mean, shape=type), size=4) +
    geom_line(data=binMeans, aes(x=as.numeric(as.factor(bin)), y=grand_mean, color=type, linetype=type), size=1) + 
    scale_fill_manual(name="Stimulus type",
                      values = c("Nonword" = "black", "Word" = "blue"),
                      guide = guide_legend(reverse = TRUE)) +  
    scale_shape_manual(name="Stimulus type",
                      values = c("Nonword" = 21, "Word" = 24),
                      guide = guide_legend(reverse = TRUE)) +   
    scale_color_manual(name="Effect estimate",
                       values = c("Nonword"="black", "Word" = "blue"),
                       guide=FALSE) +
    scale_linetype_manual(name="Effect estimate",
                          values = c("Nonword" = "solid", "Word" = "dotted"),
                          guide=FALSE) + 
    labs(y = "Mean Rating (by stimulus)",x = "Frequency Bin") +
    theme_classic()

remove(binMeans)
```

Figure S2: Mean wordhood confidence ratings for each stimulus per frequency bin. Bin1 contains the most frequent real words and Bin5 contains the least frequent real words, together with their phonotactically matched nonwords. Points represent mean ratings across all real words and nonwords within each bin (joined by lines as a visual aid only).

### 2.3.2 Mean rating and phonotactic score for each stimulus per frequency bin

Words appear to receive higher ratings than nonwords, across all frequency bins and phonotactic scores. For both words and nonwords, ratings appear to increase with phonotactic score.

This is Figure 1 in the main paper.

```
# Code for Figure 1: Plot ratings per bin, per stimulus type, against phonotactic score

scores <- unique(dataExp1[,c("word","score")])
allBinsScored <- merge(allBins, scores, by="word")
remove(scores)

ggplot(allBinsScored, aes(x=score, y=meanRating, color=type)) +
  geom_point(aes(shape=type), alpha=0.3, size=3) +
  geom_smooth(aes(fill=type), method="lm", formula="y~x", alpha=0.6, size=1) +
  facet_grid(. ~ bin) +
  xlab("Phonotactic score") +
  ylab("Mean rating (per stimulus)") +
  scale_shape_manual(name="Stimulus type",
                    values = c("Nonword" = 1, "Word" = 2),
                    guide = guide_legend(reverse = TRUE)) +   
  scale_color_manual(name="Stimulus type",
                     values = c("Nonword"="black", "Word" = "blue"),
                     guide = guide_legend(reverse = TRUE)) +
  scale_fill_manual(name="Stimulus type",
                     values = c("Nonword"="gray70", "Word" = "dodgerblue1"),
                     guide = guide_legend(reverse = TRUE)) +  
  theme_bw() + 
  theme(
    panel.grid = element_blank()
  )

remove(allBins, allBinsScored)
```

Figure 1: Mean rating vs. phonotactic score for each stimulus for real words and nonwords by frequency bin. Lines show correlations within each bin, for each stimulus type.

## 2.4 Rematching and filtering word-nonword pairs

Under the improved method for calculating phonotactic scores used in the analysis (cf. the original method used for stimulus generation and selection), many original word-nonword pairs have a larger difference in phonotactic scores than originally intended. This can be seen clearly in Figure 1, where the phonotactic scores for word and nonword stimuli only overlap in the middle range. The large gap between the phonotactic scores of words and nonwords raises the possibility that the apparent rating differences are driven by differences in phonotactic score.

To address this issue, we re-match the stimulus pairs and filter based on these new matches. We work through nonwords in decreasing order of phonotactic score and re-match each one with the as-yet-unrematched word of the same length with the closest score (from any frequency bin). We then discard any word-nonword pairs where the difference in score is greater than 0.1 (cf. the original difference threshold of 0.3). This stricter threshold on phonotactic score difference means that, at worst, one item in the pair could have approximately 1.25 times the phonotactic probability of the other.

This filtering process removes reponses to 1,552 (77.6%) of the original 2,000 stimuli; after filtering, we are left with 57 stimulus pairs in Bin1, 39 pairs in Bin2, 38 pairs in Bin3, 44 pairs in Bin4, and 46 pairs in Bin5 (each down from 200 pairs originally).

```
# Rematch stimulus pairs: for each nonword, find a word with the lowest absolute difference

listPseudo <- unique(dataExp1[dataExp1$type=="pseudo",c("word","score","length")])
listPseudo$matched = NA
listPseudo <- listPseudo[order(listPseudo$score, decreasing=TRUE),]
listReal <- unique(dataExp1[dataExp1$type=="real",c("word","score","length","bin")])
listReal$matched = NA

for(pseudo_i in 1:nrow(listPseudo)) {
  length = listPseudo[pseudo_i, "length"]
  pseudo_word = listPseudo[pseudo_i, "word"]
  pseudo_score = listPseudo[pseudo_i, "score"]
  
  real_candidates = listReal[listReal$length==length & is.na(listReal$matched),]
  real_word = real_candidates[which.min(abs(pseudo_score - real_candidates$score)), "word"]
  real_i = which(listReal$word==real_word)
  real_score = listReal[real_i, "score"]
  bin = listReal[real_i, "bin"]
  
  score_diff = abs(pseudo_score - real_score)
  
  listPseudo[pseudo_i, c("bin", "matched", "score_diff")] = c(bin, real_word, score_diff)
  listReal[real_i, c("matched", "score_diff")] = c(pseudo_word, score_diff)
}

listAll <- rbind(listPseudo, listReal)
dataRematched <- merge(dataExp1[, names(dataExp1) != "bin"], listAll)
remove(listPseudo, listReal, listAll, pseudo_i, real_i, length, bin, score_diff, pseudo_word, real_word, pseudo_score, real_score, real_candidates)

dataRematched <- dataRematched[dataRematched$score_diff < 0.1,]
```

### 2.4.1 Distributions of ratings across bins and stimulus types (rematched)

Among the rematched pairs, there is much greater overlap in the ratings of words and nonwords, which is expected because the rematching removes undue influence of words with extremely high phonotactic scores and nonwords with extremely low phonotactic scores. Crucially, however, the ratings still appear somewhat distinct, with words being rated higher than nonwords across all frequency bins.

```
# Code for Figure S3: Plot mean ratings per bin, per stimulus type, for rematched stimulus pairs

#Get mean ratings for each stimulus per bin
allRematchedBins <- list()
for(i in 1:5){
  Bin <- dataRematched[dataRematched$bin==i,]
  BinMeanRatings <- aggregate(as.numeric(Bin$enteredResponse), by=list(Bin$word, Bin$type), mean)
  names(BinMeanRatings) <- c("word","type","meanRating")
  BinMeanRatings$bin <- paste0("Bin",i)
  allRematchedBins[[i]] <- BinMeanRatings
}
remove(i, Bin, BinMeanRatings)

allRematchedBins <- do.call(rbind,allRematchedBins)
allRematchedBins$type = factor(allRematchedBins$type, labels=c("Nonword", "Word"))

# Get grand mean ratings for each stimulus type per bin
rematchedBinMeans <- allRematchedBins %>% 
  group_by(type, bin) %>% 
  summarize(grand_mean = mean(meanRating))

ggplot(allRematchedBins,aes(x = bin, y = meanRating, fill = type)) +
    geom_violin(alpha=0.4, position=position_identity()) + 
    geom_point(data=rematchedBinMeans, aes(y=grand_mean, shape=type), size=4) +
    geom_line(data=rematchedBinMeans, aes(x=as.numeric(as.factor(bin)), y=grand_mean, color=type, linetype=type), size=1) + 
    scale_fill_manual(name="Stimulus type",
                      values = c("Nonword" = "black", "Word" = "blue"),
                      guide = guide_legend(reverse = TRUE)) +  
    scale_shape_manual(name="Stimulus type",
                      values = c("Nonword" = 21, "Word" = 24),
                      guide = guide_legend(reverse = TRUE)) +   
    scale_color_manual(name="Effect estimate",
                       values = c("Nonword"="black", "Word" = "blue"),
                       guide=FALSE) +
    scale_linetype_manual(name="Effect estimate",
                          values = c("Nonword" = "solid", "Word" = "dotted"),
                          guide=FALSE) + 
    labs(y = "Mean Rating (by stimulus)",x = "Frequency Bin") +
    theme_classic()

remove(allRematchedBins, rematchedBinMeans)
```

Figure S3: Mean wordhood confidence ratings for each stimulus per frequency bin. Bin1 contains the most frequent real words and Bin5 contains the least frequent real words, together with their phonotactically matched nonwords (after rematching and filtering). Points represent mean ratings across all real words and nonwords within each bin (joined by lines as a visual aid only).

### 2.4.2 Mean rating and phonotactic score for each stimulus per frequency bin (rematched)

Among the rematched pairs, in addition to the ratings of words and nonwords still appearing somewhat distinct, both still appear to increase with phonotactic probability.

```
# Code for Figure S4: Plot ratings per bin, per stimulus type, against phonotactic score, for rematched stimulus pairs

dataRematched %>%
  group_by(bin, word, score) %>%
  summarise(
    type = if_else(type=="pseudo", "Nonword", "Word")[1],
    meanRating = mean(enteredResponse)
  ) %>%
  ggplot(., aes(x=score, y=meanRating, color=type)) +
    geom_point(aes(shape=type), alpha=0.6, size=3) +
    geom_smooth(aes(fill=type), method="lm", formula="y~x", alpha=0.4, size=1) +
    facet_grid(. ~ bin, labeller=labeller(bin=function(n) paste0("Bin",n))) +
    xlab("Phonotactic score") +
    ylab("Mean rating (per stimulus)") +
    scale_shape_manual(name="Stimulus type",
                      values = c("Nonword" = 1, "Word" = 2),
                      guide = guide_legend(reverse = TRUE)) +   
    scale_color_manual(name="Stimulus type",
                       values = c("Nonword"="black", "Word" = "blue"),
                       guide = guide_legend(reverse = TRUE)) +
    scale_fill_manual(name="Stimulus type",
                       values = c("Nonword"="gray70", "Word" = "dodgerblue1"),
                       guide = guide_legend(reverse = TRUE)) + 
    scale_x_continuous(breaks=seq(-1.2, -0.8, by=0.2)) +  
    theme_bw() + 
    theme(
      panel.grid = element_blank()
    )
```

Figure S4: Mean rating vs. phonotactic score for each stimulus for real words and nonwords by frequency bin (after rematching and filtering). Lines show correlations within each bin, for each stimulus type.

## 2.5 Statistical analysis

We use mixed-effects ordinal regression to predict participants’ wordhood confidence ratings. For an introduction to ordinal regression, see the Detailed Materials and Methods, Section 5.1.

The predictors considered in the analysis are:

- `length`: Stimulus length in phonemes (centered)
- `score`: Stimulus phonotactic score (centered)
- `type`: Stimulus type: word or nonword (binary factor)
- `bin`: Frequency bin (Helmert-coded factor)
  - Note: we use manually-defined Helmert contrasts rather than the built-in `contr.helmert` function, which performs reverse Helmert coding

We consider `score` and `type` to be the predictors of primary interest, while `length` and `bin` are control predictors.

For pratical reasons of computing time, we perform a step-down model fitting procedure in a fixed-effects setting (`clm`) before transitioning to a mixed-effects setting (`clmm`). We begin by considering a model containing all two-way interactions between a predictor of primary interest (`score` and `type`) and a control predictor (`length` and `bin`), as well as the interaction between the two predictors of primary interest. We remove interactions one at a time if they do not make a significant contribution to the model’s explanatory power, assessed via Analysis of Deviance (using a type III test from the `anova` function), starting with the interaction with the least contribution. We then remove main effects one a time if they are not included in any remaining interactions and they do not make a significant contribution to the model’s explanatory power. Once there are no more candidate terms for removal, we re-fit the model in a mixed-effects setting, adding random intercepts by participant and item and random slopes by participant for each remaining term. For practical reasons of computing time, we identify candidates for removal in the mixed-effects setting if their coefficient is not significant in the model; after performing each removal, we confirm that it was justified via model comparison, using a log-likelihood ratio test.

We first analyze the original dataset, attempting to control for the previously-identified phonotactic differences between words and nonwords statistically. We then analyze the subset of the data with rematched and filtered stimulus pairs, re-applying the model fitting procedure. This subset of the data enforces a strong control over phonotactic differences between words and nonwords and enables us to be confident that the main significant effects reported by the regression model are real. We note, however, that the extensive filtering used for this dataset means that we have limited ability to rule out other (insignificant) effects due to issues of statistical power.

### 2.5.1 Original dataset

Table S1 reports the results of the ordinal regression analysis for the original (full) dataset.

We see a significant main effect of stimulus `type`, indicating that real words are rated higher than nonwords. Additionally, we see a significant interaction between stimulus `type` and frequency `bin` in Bin1, indicating that the highest-frequency real words are rated higher relative to their matched nonwords than would be expected based on all other words and nonwords.

We also see a significant main effect of phonotactic `score`, where stimuli with higher phonotactic scores receive higher ratings.

Finally, we see a significant main effect of stimulus `length` and a significant interaction between `length` and `type`, indicating that nonwords are rated lower as their length increases, while real words are not.

For ease of interpretation, we present partial effect plots in Figure S5.

```
# Code for Table S1: statistical analysis of Exp1

dataExp1$enteredResponse <- as.factor(dataExp1$enteredResponse)

helmert <- matrix(c(c(4,-1,-1,-1,-1)/5, c(0,3,-1,-1,-1)/4, c(0,0,2,-1,-1)/3, c(0,0,0,1,-1)/2), ncol=4)
colnames(helmert) <- c("1v2+", "2v3+", "3v4+", "4v5")
dataExp1$bin <- as.factor(dataExp1$bin)
contrasts(dataExp1$bin) <- helmert
remove(helmert)

# Model fitting process: fixed-effects only
# m1 <- clm(enteredResponse ~ (c.(score) + type) * (c.(length) + bin) + c.(score):type, data=dataExp1)
# anova(m1, type="III") # Removal candidate: c.(score):type
# m2 <- update(m1, . ~ . - c.(score):type)
# anova(m2, type="III") # Removal candidate: c.(score):c.(length)
# m3 <- update(m2, . ~ . - c.(score):c.(length))
# anova(m3, type="III") # Removal candidate: c.(score):bin
# m4 <- update(m3, . ~ . - c.(score):bin)
# anova(m4, type="III") # No further candidate predictors for removal. Switch to mixed-effects
# m5 <- clmm(enteredResponse ~ c.(score) + type * (c.(length) + bin) + (1 + c.(score) + type * (c.(length) + bin)|workerId) + (1|word), data=dataExp1)
# summary(m5) # No candidate predictors for removal
# saveRDS(m5, file = "dumps/clmm/modelTableS1.rds")
# remove(m1, m2, m3, m4, m5)
modelTableS1 <- readRDS("dumps/clmm/modelTableS1.rds")

clm_table(modelTableS1, caption="Table S1: Ordinal mixed-effects model of NMS wordhood confidence ratings. All numeric variables in this model except for the wellformedness rating are centered.")
```

Table S1: Ordinal mixed-effects model of NMS wordhood confidence ratings. All numeric variables in this model except for the wellformedness rating are centered.

|  | Parameter | Estimate | Std. Error | \(z\) | \(p\) |  |
| --- | --- | --- | --- | --- | --- | --- |
| Effects | score (centered) | 2.060 | 0.233 | 8.847 | <0.001 | \*\*\* |
|  | type = real | 0.719 | 0.093 | 7.737 | <0.001 | \*\*\* |
|  | length (centered) | -0.043 | 0.022 | -1.971 | 0.049 | \* |
|  | bin = 1v2+ | -0.017 | 0.087 | -0.199 | 0.842 |  |
|  | bin = 2v3+ | 0.096 | 0.086 | 1.109 | 0.267 |  |
|  | bin = 3v4+ | 0.054 | 0.092 | 0.582 | 0.560 |  |
|  | bin = 4v5 | 0.130 | 0.105 | 1.230 | 0.219 |  |
|  | type = real × length (centered) | 0.073 | 0.024 | 3.062 | 0.002 | \*\* |
|  | type = real × bin = 1v2+ | 0.468 | 0.133 | 3.532 | <0.001 | \*\*\* |
|  | type = real × bin = 2v3+ | 0.102 | 0.127 | 0.804 | 0.421 |  |
|  | type = real × bin = 3v4+ | 0.072 | 0.130 | 0.558 | 0.577 |  |
|  | type = real × bin = 4v5 | -0.038 | 0.151 | -0.253 | 0.800 |  |
| Thresholds | 1|2 | -2.609 | 0.119 |  |  |  |
|  | 2|3 | -0.805 | 0.118 |  |  |  |
|  | 3|4 | 1.107 | 0.118 |  |  |  |
|  | 4|5 | 3.057 | 0.119 |  |  |  |

```
# Code for Figure S5: statistical analysis of Exp1

figS5a.mean <- clm_plotdat(modelTableS1, c("type", "bin"), type="mean") %>%
  mutate(
    type = fct_recode(type, "Nonword"="pseudo", "Word"="real") %>% fct_relevel("Word")
  ) %>%
  ggplot(., aes(x=as.integer(bin), y=pred, color=type, fill=type, shape=type)) +
    geom_ribbon(aes(ymin=lci, ymax=uci), alpha=0.1, color=NA) +
    geom_line(size=1, alpha=0.4) +
    geom_point(size=4) +
    geom_errorbar(aes(ymin=lci, ymax=uci), size=1, width=0.3) +
    xlab("Frequency Bin (High to Low)") +
    ylab("Predicted mean rating") + 
    scale_shape_manual(values = c("Nonword" = 19, "Word" = 17)) + 
    scale_color_manual(values = c("Nonword" = "black", "Word" = "blue")) +
    scale_fill_manual(values = c("Nonword" = "black", "Word" = "blue")) +
    theme_classic() + 
    theme(
      plot.title = element_text(hjust = 0.5, size=15),
      legend.position="none",
      legend.title=element_blank(),
      panel.background = element_blank()
    )

figS5a.dist <- clm_plotdat(modelTableS1, c("type", "bin"), type="dist") %>%
  mutate(
    type = fct_recode(type, "Nonword"="pseudo", "Word"="real") %>% fct_relevel("Word")
  ) %>%  
  ggplot(aes(x=as.integer(bin), y=pred, color=type, fill=type, shape=type)) +
    geom_ribbon(aes(ymin=lci, ymax=uci), alpha=0.2, color=NA) +
    geom_line(size=1, alpha=0.4) +
    geom_point(size=2) +
    geom_errorbar(aes(ymin=lci, ymax=uci), size=1, width=0.3) +
    facet_grid(. ~ response, labeller=as_labeller(function(x) str_c("Rating: ", x))) +
    ylim(0, 1) +
    xlab("Frequency Bin (High to Low)") +
    ylab("Probability of rating") + 
    scale_shape_manual(values = c("Nonword" = 19, "Word" = 17)) + 
    scale_color_manual(values = c("Nonword" = "black", "Word" = "blue")) +
    scale_fill_manual(values = c("Nonword" = "black", "Word" = "blue")) +
    theme_bw() +
    theme(
      plot.title = element_text(hjust = 0.5, size=15),
      legend.position="right",
      legend.title=element_blank()
    )  

figS5b.mean <- clm_plotdat(modelTableS1, "score", xlevels=25, type="mean") %>%
  mutate(type="Word and\nNonword") %>%
  ggplot(., aes(x=score, y=pred, color=type, fill=type)) +
    geom_ribbon(aes(ymin=lci, ymax=uci), alpha=0.3, color=NA) +
    geom_line(size=1) +
    scale_x_continuous(n.breaks=4) +
    xlab("Phonotactic score") +
    ylab("Predicted mean rating") + 
    scale_color_manual(values = c("Word and\nNonword" = "purple")) +
    scale_fill_manual(values = c("Word and\nNonword" = "purple")) +    
    theme_classic() + 
    theme(
      plot.title = element_text(hjust = 0.5, size=15),
      legend.position="none",
      legend.title=element_blank(),
      panel.background = element_blank()
    )

figS5b.dist <- clm_plotdat(modelTableS1, "score", xlevels=25, type="dist") %>%
  mutate(type="Word and\nNonword") %>%
  ggplot(., aes(x=score, y=pred, color=type, fill=type)) +
    geom_ribbon(aes(ymin=lci, ymax=uci), alpha=0.3, color=NA) +
    geom_line(size=1) +
    scale_x_continuous(n.breaks=4) +
    xlab("Phonotactic score") +
    ylab("Probability of rating") + 
    scale_color_manual(values = c("Word and\nNonword" = "purple")) +
    scale_fill_manual(values = c("Word and\nNonword" = "purple")) +  
    facet_grid(. ~ response, labeller=as_labeller(function(x) str_c("Rating: ", x))) +
    ylim(0, 1) +  
    theme_bw() + 
    theme(
      plot.title = element_text(hjust = 0.5, size=15),
      legend.position="right",
      legend.title=element_blank()
    ) 

figS5c.mean <- clm_plotdat(modelTableS1, c("type", "length"), xlevels=list(length=25), type="mean") %>%
  mutate(
    type = fct_recode(type, "Nonword"="pseudo", "Word"="real") %>% fct_relevel("Word")
  ) %>%
  ggplot(., aes(x=length, y=pred, color=type, fill=type)) +
    geom_ribbon(aes(ymin=lci, ymax=uci), alpha=0.2, color=NA) +
    geom_line(size=1) +
    xlab("Phoneme length") +
    ylab("Predicted mean rating") + 
    scale_color_manual(values = c("Nonword" = "black", "Word" = "blue")) +
    scale_fill_manual(values = c("Nonword" = "black", "Word" = "blue")) +
    theme_classic() + 
    theme(
      plot.title = element_text(hjust = 0.5, size=15),
      legend.position="none",
      legend.title=element_blank(),
      panel.background = element_blank()
    )

figS5c.dist <- clm_plotdat(modelTableS1, c("type", "length"), xlevels=list(length=25), type="dist") %>%
  mutate(
    type = fct_recode(type, "Nonword"="pseudo", "Word"="real") %>% fct_relevel("Word")
  ) %>%
  ggplot(., aes(x=length, y=pred, color=type, fill=type)) +
    geom_ribbon(aes(ymin=lci, ymax=uci), alpha=0.2, color=NA) +
    geom_line(size=1) +
    xlab("Phoneme length") +
    ylab("Probability of rating") + 
    scale_color_manual(values = c("Nonword" = "black", "Word" = "blue")) +
    scale_fill_manual(values = c("Nonword" = "black", "Word" = "blue")) +
    facet_grid(. ~ response, labeller=as_labeller(function(x) str_c("Rating: ", x))) +
    ylim(0, 1) +  
    theme_bw() + 
    theme(
      plot.title = element_text(hjust = 0.5, size=15),
      legend.position="right",
      legend.title=element_blank()
    )

ggarrange(figS5a.mean, figS5a.dist, figS5b.mean, figS5b.dist, figS5c.mean, figS5c.dist, ncol=2, labels=c("a.", "", "b.", "", "c.", ""), widths=c(1,5))

remove(figS5a.mean, figS5a.dist, figS5b.mean, figS5b.dist, figS5c.mean, figS5c.dist)
```

Figure S5: Partial effect plots for ordinal mixed-effects model of NMS wordhood confidence ratings. Left-hand plots show predicted mean ratings; right-hand plots show predicted distributions over ratings (facets). Figure S5a (top) shows the interaction between frequency bin and lexicality (real words vs. nonwords); Figure S5b (middle) shows the effect of phonotactic score; Figure S5c (bottom) shows the interaction between stimulus length and lexicality (real words vs. nonwords). Error bars and ribbons represent 95% confidence intervals.

### 2.5.2 Subset of dataset with rematched and filtered stimulus pairs

Table S2 reports the results of the ordinal regression analysis for the subset of the dataset after rematching and filtering of stimulus pairs.

It is noteworthy that we still see a significant main effect of `type`, indicating that real words are rated higher than nonwords, and a significant main effect of phonotactic `score`, where stimuli with higher phonotactic scores receive higher ratings. These core results confirm that, while NMS’ ratings are affected by phonotactic knowledge, their ability to distinguish between words and nonwords is not simply an artefact of that phonotactic knowledge.

Instead of the interaction between frequency `bin` and stimulus `type` in Bin1 observed before, we now see a significant main effect of `bin` in Bin1 across both words and nonwords. However, we cannot be sure that this effect is not driven by real words as was the case previously, due to the substantial drop in statistical power.

For ease of interpretation, we present partial effect plots in Figure S6.

```
# Code for Table S2: statistical analysis for Exp1, restricted to rematched stimulus pairs

dataRematched$enteredResponse <- as.factor(dataRematched$enteredResponse)

helmert <- matrix(c(c(4,-1,-1,-1,-1)/5, c(0,3,-1,-1,-1)/4, c(0,0,2,-1,-1)/3, c(0,0,0,1,-1)/2), ncol=4)
colnames(helmert) <- c("1v2+", "2v3+", "3v4+", "4v5")
dataRematched$bin <- as.factor(dataRematched$bin)
contrasts(dataRematched$bin) <- helmert
remove(helmert)

# score and type are variables of interest; length and bin are controls. Start by testing all two-way interactions between interest and controls, plus score:type.

# r1 <- clm(enteredResponse ~ (c.(score) + type) * (c.(length) + bin) + c.(score):type, data=dataRematched)
# anova(r1, type="III") # Removal candidate: type:bin
# r2 <- update(r1, . ~ . - type:bin)
# anova(r2, type="III") # Removal candidate: type:c.(length)
# r3 <- update(r2, . ~ . - type:c.(length))
# anova(r3, type="III") # Removal candidate: c.(score):type
# r4 <- update(r3, . ~ . - c.(score):type) 
# anova(r4, type="III") # No further candidate predictors for removal. Switch to mixed-effects
# r5 <- clmm(enteredResponse ~ type + c.(score) * (c.(length) + bin) + (1 + type + c.(score) + c.(length) + bin|workerId) + (1|word), data=dataRematched) # Note that we have had to remove the random slopes by workerId for all interaction terms, for reasons of convergence
# summary(r5) # Removal candidate: c.(score):c.(length)
# r6 <- update(r5, . ~ . - c.(score):c.(length))
# anova(r5, r6) # Removal justified!
# summary(r6) # Removal candidate: c.(score):bin
# r7 <- update(r6, . ~ . - c.(score):bin)
# anova(r6, r7) # Removal justified!
# summary(r7) # Removal candidate: c.(length) and associated random slope
# r8 <- clmm(enteredResponse ~ type + c.(score) + bin + (1 + type + c.(score) + bin|workerId) + (1|word), data=dataRematched)
# anova(r7, r8) # Removal not justified; end removal procedure
# saveRDS(r7, file = "dumps/clmm/modelTableS2.rds")
# remove(r1, r2, r3, r4, r5, r6, r7, r8)
modelTableS2 <- readRDS("dumps/clmm/modelTableS2.rds")

clm_table(modelTableS2, caption="Table S2: Ordinal mixed-effects model of NMS wordhood confidence ratings (for rematched stimulus pairs). All numeric variables in this model except for the wellformedness rating are centered.")
```

Table S2: Ordinal mixed-effects model of NMS wordhood confidence ratings (for rematched stimulus pairs). All numeric variables in this model except for the wellformedness rating are centered.

|  | Parameter | Estimate | Std. Error | \(z\) | \(p\) |  |
| --- | --- | --- | --- | --- | --- | --- |
| Effects | type = real | 0.498 | 0.107 | 4.673 | <0.001 | \*\*\* |
|  | score (centered) | 2.513 | 0.648 | 3.880 | <0.001 | \*\*\* |
|  | length (centered) | 0.059 | 0.037 | 1.606 | 0.108 |  |
|  | bin = 1v2+ | 0.466 | 0.130 | 3.588 | <0.001 | \*\*\* |
|  | bin = 2v3+ | 0.139 | 0.142 | 0.980 | 0.327 |  |
|  | bin = 3v4+ | 0.334 | 0.146 | 2.285 | 0.022 | \* |
|  | bin = 4v5 | 0.071 | 0.156 | 0.456 | 0.649 |  |
| Thresholds | 1|2 | -2.548 | 0.131 |  |  |  |
|  | 2|3 | -0.784 | 0.124 |  |  |  |
|  | 3|4 | 1.064 | 0.125 |  |  |  |
|  | 4|5 | 2.856 | 0.131 |  |  |  |

```
# Code for Figure S6: statistical analysis for Exp1, restricted to rematched stimulus pairs

figS6a.mean <- clm_plotdat(modelTableS2, c("type", "bin"), type="mean") %>%
  mutate(
    type = fct_recode(type, "Nonword"="pseudo", "Word"="real") %>% fct_relevel("Word")
  ) %>%
  ggplot(., aes(x=as.integer(bin), y=pred, color=type, fill=type, shape=type)) +
    geom_ribbon(aes(ymin=lci, ymax=uci), alpha=0.1, color=NA) +
    geom_line(size=1, alpha=0.4) +
    geom_point(size=4) +
    geom_errorbar(aes(ymin=lci, ymax=uci), size=1, width=0.3) +
    xlab("Frequency Bin (High to Low)") +
    ylab("Predicted mean rating") + 
    scale_shape_manual(values = c("Nonword" = 19, "Word" = 17)) + 
    scale_color_manual(values = c("Nonword" = "black", "Word" = "blue")) +
    scale_fill_manual(values = c("Nonword" = "black", "Word" = "blue")) +
    theme_classic() + 
    theme(
      plot.title = element_text(hjust = 0.5, size=15),
      legend.position="none",
      legend.title=element_blank(),
      panel.background = element_blank()
    )

figS6a.dist <- clm_plotdat(modelTableS2, c("type", "bin"), type="dist") %>%
  mutate(
    type = fct_recode(type, "Nonword"="pseudo", "Word"="real") %>% fct_relevel("Word")
  ) %>%
  ggplot(., aes(x=as.integer(bin), y=pred, color=type, fill=type, shape=type)) +
    geom_ribbon(aes(ymin=lci, ymax=uci), alpha=0.2, color=NA) +
    geom_line(size=1, alpha=0.4) +
    geom_point(size=2) +
    geom_errorbar(aes(ymin=lci, ymax=uci), size=1, width=0.3) +
    xlab("Frequency Bin (High to Low)") +
    ylab("Probability of rating") + 
    scale_shape_manual(values = c("Nonword" = 19, "Word" = 17)) + 
    scale_color_manual(values = c("Nonword" = "black", "Word" = "blue")) +
    scale_fill_manual(values = c("Nonword" = "black", "Word" = "blue")) +
    facet_grid(. ~ response, labeller=as_labeller(function(x) str_c("Rating: ", x))) +
    ylim(0, 1) +  
    theme_bw() + 
    theme(
      plot.title = element_text(hjust = 0.5, size=15),
      legend.position="right",
      legend.title=element_blank(),
      panel.background = element_blank()
    )

figS6b.mean <- clm_plotdat(modelTableS2, "score", xlevels=25, type="mean") %>%
  mutate(type="Word and\nNonword") %>%
  ggplot(., aes(x=score, y=pred, color=type, fill=type)) +
    geom_ribbon(aes(ymin=lci, ymax=uci), alpha=0.2, color=NA) +
    geom_line(size=1) +
    xlab("Phonotactic score") +
    ylab("Predicted mean rating") +
    scale_x_continuous(n.breaks=4) +
    scale_color_manual(values = c("Word and\nNonword" = "purple")) +
    scale_fill_manual(values = c("Word and\nNonword" = "purple")) +   
    theme_classic() + 
    theme(
      plot.title = element_text(hjust = 0.5, size=15),
      legend.position = "none",
      legend.title=element_blank(),
      panel.background = element_blank()
    )

figS6b.dist <- clm_plotdat(modelTableS2, "score", xlevels=25, type="dist") %>%
  mutate(type="Word and\nNonword") %>%
  ggplot(., aes(x=score, y=pred, color=type, fill=type)) +
    geom_ribbon(aes(ymin=lci, ymax=uci), alpha=0.3, color=NA) +
    geom_line(size=1) +
    xlab("Phonotactic score") +
    ylab("Probability of rating") + 
    scale_x_continuous(n.breaks=4) +
    scale_color_manual(values = c("Word and\nNonword" = "purple")) +
    scale_fill_manual(values = c("Word and\nNonword" = "purple")) +   
    facet_grid(. ~ response, labeller=as_labeller(function(x) str_c("Rating: ", x))) +
    ylim(0, 1) +  
    theme_bw() + 
    theme(
      plot.title = element_text(hjust = 0.5, size=15),
      legend.position = "right",
      legend.title=element_blank(),
      panel.background = element_blank()
    )

figS6c.mean <- clm_plotdat(modelTableS2, c("type", "length"), xlevels=list(length=25), type="mean") %>%
  mutate(
    type = fct_recode(type, "Nonword"="pseudo", "Word"="real") %>% fct_relevel("Word")
  ) %>%
  ggplot(., aes(x=length, y=pred, color=type, fill=type)) +
    geom_ribbon(aes(ymin=lci, ymax=uci), alpha=0.1, color=NA) +
    geom_line(size=1) +
    xlab("Phoneme length") +
    ylab("Predicted mean rating") + 
    scale_color_manual(values = c("Nonword" = "black", "Word" = "blue")) +
    scale_fill_manual(values = c("Nonword" = "black", "Word" = "blue")) +
    theme_classic() + 
    theme(
      plot.title = element_text(hjust = 0.5, size=15),
      legend.position="none",
      legend.title=element_blank(),
      panel.background = element_blank()
    )

figS6c.dist <- clm_plotdat(modelTableS2, c("type", "length"), xlevels=list(length=25), type="dist") %>%
  mutate(
    type = fct_recode(type, "Nonword"="pseudo", "Word"="real") %>% fct_relevel("Word")
  ) %>%
  ggplot(., aes(x=length, y=pred, color=type, fill=type)) +
    geom_ribbon(aes(ymin=lci, ymax=uci), alpha=0.2, color=NA) +
    geom_line(size=1) +
    xlab("Phoneme length") +
    ylab("Probability of rating") + 
    scale_color_manual(values = c("Nonword" = "black", "Word" = "blue")) +
    scale_fill_manual(values = c("Nonword" = "black", "Word" = "blue")) +
    facet_grid(. ~ response, labeller=as_labeller(function(x) str_c("Rating: ", x))) +
    ylim(0, 1) +  
    theme_bw() + 
    theme(
      plot.title = element_text(hjust = 0.5, size=15),
      legend.position="right",
      legend.title=element_blank(),
      panel.background = element_blank()
    )

ggarrange(figS6a.mean, figS6a.dist, figS6b.mean, figS6b.dist, figS6c.mean, figS6c.dist, ncol=2, labels=c("a.", "", "b.", "", "c.", ""), widths=c(1,5))

remove(figS6a.mean, figS6a.dist, figS6b.mean, figS6b.dist, figS6c.mean, figS6c.dist)
```

Figure S6: Partial effect plots for ordinal mixed-effects model of NMS wordhood confidence ratings (for rematched stimulus pairs). The figure is laid out in the same way as Figure S5. Left-hand plots show predicted mean ratings; right-hand plots show predicted distributions over ratings (facets). Figure S6a (top) shows the effects of frequency bin and lexicality (real words vs. nonwords); Figure S6b (middle) shows the effect of phonotactic score; Figure S6c (bottom) shows the effects of stimulus length and lexicality (real words vs. nonwords). Error bars and ribbons represent 95% confidence intervals.

# 3 Experiment 2: Well-formedness rating task

In Experiment 1, Māori-speaking New Zealanders (MS), non-Māori-speaking New Zealanders (NMS), and English-speaking Americans (US) assigned well-formedness ratings to nonwords with varying phonotactic scores.

We provide an overview of participants’ demographics below; for further details of participants, including exclusion criteria, see Detailed Materials and Methods Section 1.2. For details of the stimuli, see Detailed Materials and Methods Section 2.2. For details of the procedure, see Detailed Materials and Methods Section 3.2.

```
# Load the data for Exp2 and filter participants

dataExp2 <- read.delim("./data/dataAnonNotFilteredExp2.txt", sep ="\t", header = TRUE)
dataExp2$word <- as.character(dataExp2$word)
Encoding(dataExp2$word) = "UTF-8"
dataExp2$item <- as.character(dataExp2$item)

# Part 2: Removing unusable NMS participants
dataNMS <- dataExp2[dataExp2$group=="NMS",]

# Move 6 participants whose speakMaori and compMaori are at least (equal to or above) 3 to the MS group
moveParticipants <- unique(dataNMS[dataNMS$speakMaori >= 3 & dataNMS$compMaori >= 3, "workerId"]) # 13076185 fa784dba a9695b3e 10c02fd0 379bf450 91950415
dataExp2[dataExp2$workerId %in% moveParticipants, "group"] <- "MS"
dataNMS <- dataNMS[!(dataNMS$workerId %in% moveParticipants),]
remove(moveParticipants)

# Remove nine participants whose speakMaori or compMaori is at least (equal to or above) 3
rmParticipant1 <- unique(dataNMS[dataNMS$speakMaori >= 3 | dataNMS$compMaori >= 3,]$workerId)  # 3cde3d04 dab57fd5 945a57c8 8f889ddc 8c51cdc1 930984af 61dd030b 3efd81e0 72d13b4e
dataNMS <- dataNMS[!dataNMS$workerId %in% rmParticipant1,]
remove(rmParticipant1)

# Remove ten participants who did not learn their English in NZ and have been living in their current location in NZ for less than ten years (duration == "short")
summaryNMSWorkerId <- unique(dataNMS[,c("workerId","firstLangCountry","place","duration")])
EngNotInNZ <- summaryNMSWorkerId[!summaryNMSWorkerId$firstLangCountry=="NZ",]
rmParticipant2 <- unique(EngNotInNZ[EngNotInNZ$duration=="short",]$workerId) # 8332d72f 70dafe1e 4f8b6f05 d3083fc5 e53ac07a 17f67c54 8277a12b 6a1740c7 29d42922 9a7885b3
dataNMS <- dataNMS[!dataNMS$workerId %in% rmParticipant2,]
remove(summaryNMSWorkerId, EngNotInNZ, rmParticipant2)

#Remove one participant who has lived in Hawaii
rmParticipant3 <- unique(dataNMS[dataNMS$hawaii=="Yes",]$workerId) # fc35cab1
dataNMS <- dataNMS[!dataNMS$workerId %in% rmParticipant3,]
remove(rmParticipant3)

# Remove four participants who know any other Polynesian languages
rmParticipant4 <- unique(dataNMS[dataNMS$anyPolynesian=="Yes",]$workerId) # f7466ae0 519ab1a7 a8ef4bcb bdabfcc5
dataNMS <- dataNMS[!dataNMS$workerId %in% rmParticipant4,]
remove(rmParticipant4)

# Remove three participants with language impairments
rmParticipant5 <- unique(dataNMS[dataNMS$impairments=="Yes",]$workerId) # 8e8c19fe 55d7e82a fc8d6ce1
dataNMS <- dataNMS[!dataNMS$workerId %in% rmParticipant5,]
remove(rmParticipant5)

#Remove one participant whose median reactionTime is shorter than 2*SD below the mean of all NMS
median_RT <- aggregate(dataNMS$reactionTime, by=list(dataNMS$workerId), median); names(median_RT) <- c("workerId","median")
cut <- mean(median_RT$median)-2*sd(median_RT$median)
rmParticipant6 <- median_RT[!median_RT$median > cut,]$workerId # 4b1aac09
dataNMS <- dataNMS[!dataNMS$workerId %in% rmParticipant6,]
remove(median_RT, cut, rmParticipant6)

# Remove four participants whose pattern of responses (SD) is below 2SD of the mean of all NMS participants
SD <- aggregate(dataNMS$enteredResponse, by=list(dataNMS$workerId), sd)
cut <- mean(SD$x)-2*sd(SD$x)
rmParticipant7 <- SD[!SD$x > cut,]$Group.1 # 3bd22196 4f737f51 6889ff72 98590568
dataNMS <- dataNMS[!dataNMS$workerId %in% rmParticipant7,]
remove(SD, cut, rmParticipant7)

# Check the total number of usable NMS participants
# length(unique(dataNMS$workerId)) #113

# Part 2: Removing unusable MS participants
dataMS <- dataExp2[dataExp2$group=="MS",]

# Remove two participants who did the wrong number of items
nbResponse <- aggregate(dataMS$enteredResponse, by=list(dataMS$workerId),  FUN=length)
rmParticipant8 <- nbResponse[!nbResponse$x %in% c(320,240),]$Group.1 # 10c02fd0 b7619945
dataMS <- dataMS[!dataMS$workerId %in% rmParticipant8,]
remove(nbResponse, rmParticipant8)

# Remove two participants whose speakMaori or compMaori is below 3
rmParticipant9 <- unique(dataMS[dataMS$speakMaori <3 | dataMS$compMaori < 3,]$workerId) # 1544dbf9 c4d7bd69
dataMS <- dataMS[!dataMS$workerId %in% rmParticipant9,]
remove(rmParticipant9)

# Remove one participant with language impairments
rmParticipant10 <- unique(dataMS[dataMS$impairments=="Yes",]$workerId) # 7fae7c22
dataMS <- dataMS[!dataMS$workerId %in% rmParticipant10,]
remove(rmParticipant10)

# Remove one participant whose pattern of responses (SD) is below 2SD of the mean of all MS participants
SD <- aggregate(dataMS$enteredResponse, by=list(dataMS$workerId), sd)
cut <- mean(SD$x)-2*sd(SD$x)
rmParticipant11 <- SD[!SD$x > cut,]$Group.1 # 379bf450
dataMS <- dataMS[!dataMS$workerId %in% rmParticipant11,]
remove(SD, cut, rmParticipant11)

# Check the total number of usable MS participants
# length(unique(dataMS$workerId)) #40

# Part3: Removing unusable US participants
dataUS <- dataExp2[dataExp2$group=="US",]

# Remove one participant who did 248 items instead of 240 (length 3)
nbResponse <- aggregate(dataUS$enteredResponse, by=list(dataUS$workerId),  FUN=length)
rmParticipant12 <- nbResponse[!nbResponse$x %in% c(320,240),]$Group.1 # 9198e3ed
dataUS <- dataUS[!dataUS$workerId %in% rmParticipant12,]
remove(nbResponse, rmParticipant12)

# Remove fourteen participants whose speakMaori or compMaori is above 0
rmParticipant13 <- unique(dataUS[dataUS$speakMaori > 0 | dataUS$compMaori > 0,]$workerId)  # 5621e137 da8aa1c2 76e49a1d 20a0b59d cd213e66 a0d0f317 349460f3 78e9bed5 8fcb2f09 84975d69 73c79807 ec169941 7c487108 89bf37e6
dataUS <- dataUS[!dataUS$workerId %in% rmParticipant13,]
remove(rmParticipant13)

# Remove six participants whose maoriList is above 0
rmParticipant14 <- unique(dataUS[dataUS$maoriList > 0,]$workerId) # 5d404970 b40035cc 7233c727 30f0793a 01446e83 c3d73f11
dataUS <- dataUS[!dataUS$workerId %in% rmParticipant14,]
remove(rmParticipant14)

# Remove one participant who did not learn English in the US
rmParticipant15 <- unique(dataUS[!dataUS$firstLangCountry=="US",]$workerId) # c7b992cc
dataUS <- dataUS[!dataUS$workerId %in% rmParticipant15,]
remove(rmParticipant15)

#Remove three participants who have been to Hawaii
rmParticipant16 <- unique(dataUS[dataUS$hawaii=="Yes",]$workerId) # d7464dfa a507847e 6f8cf2ef
dataUS <- dataUS[!dataUS$workerId %in% rmParticipant16,]
remove(rmParticipant16)

#Remove one participant with language impairments
rmParticipant17 <- unique(dataUS[dataUS$impairments=="Yes",]$workerId) # 328bd924
dataUS <- dataUS[!dataUS$workerId %in% rmParticipant17,]
remove(rmParticipant17)

# Remove one participant whose pattern of responses (SD) is below 2SD of the mean of all US participants
SD <- aggregate(dataUS$enteredResponse, by=list(dataUS$workerId), sd)
cut <- mean(SD$x)-2*sd(SD$x)
rmParticipant18 <- SD[!SD$x > cut,]$Group.1 # 7960174b
dataUS <- dataUS[!dataUS$workerId %in% rmParticipant18,]
remove(SD, cut, rmParticipant18)

# Check the total number of usable US participants
# length(unique(dataUS$workerId)) #94
dataExp2 <- rbind(dataMS, dataNMS, dataUS)
remove(dataMS, dataNMS, dataUS)

# Set up factor levels for later
dataExp2$group <- factor(dataExp2$group, levels=c("NMS", "MS", "US"))
dataExp2$enteredResponse <- as.factor(dataExp2$enteredResponse)
```

## 3.1 Dataset structure

The dataset is structured as follows:

- *workerId* is the unique ID for each participant.
  - *group* is the classification of participants according to their fluency of Māori and/or exposure to Māori (values: `NMS`, `MS`, `US`).
  - *speakMaori* is each participant’s answer to the question how well they can speak Māori (with a scale ranging from 0 to 5).
  - *compMaori* is each participant’s answer to the question how well they can understand/read Māori (with a scale ranging from 0 to 5).
  - *maoriProf* is the sum of quantified response for speakMaori and compMaori, which refers to the level of each participant’s Māori proficiency (results can range from 0 to 10).
  - *age* is the age group that each participant belongs to.
  - *gender* is the gender of each participant.
  - *ethnicity* is categorized into binary answers, either Māori (`M`) or non Māori (`non M`).
  - *education* is each participant’s highest level of education.
  - *children* is each NZ-based participant’s answer to the question whether they have had any children who have attended preschool or primary school in New Zealand in the past five years.
  - *maoriList* is each participant’s basic knowledge of Māori (with a scale ranging from 0 to 9).
  - *place* is each NZ-based participant’s current place of living (categorized into binary classification, either North or South Island in New Zealand).
  - *duration* is each participant’s time duration of living in their current place (categorized into binary classification, `long`: > 10 years and `short`: =< 10 years).
  - *firstLang* is each participant’s first language.
  - *firstLangCountry* is the country where each participant learned their first language.
  - *anyOtherLangs* is the information regarding any other languages each participant can speak.
  - *hawaii* is the binary response to the question whether participants have lived in Hawaii.
  - *anyPolynesian* is the binary response to the question whether participants know any Polynesian such as Hawaiian, Tahitian, Sāmoan, or Tongan.
  - *whichPolynesian* is the information regarding participants’ knowledge of any Polynesian languages if they knew any.
  - *impairments* is the answer to the question whether participants have a history of any speech or language impairments.
  - *maoriExpo* is each NZ-based participant’s level of exposure to Māori (with a log-like scale ranging from 2 to 10, combining media and conversational exposure).
  - *nz* is the binary response to the question whether US participants have ever lived in New Zealand.
  - *word* is the stimulus used for the rating, in standard orthgraphic form.
  - *item* is the stimulus used for the rating, in phonological form with one distinct character per phoneme (long vowels are represented by capitals; *wh* is represented by `f`, and *ng* is represented by `N`).
  - *length* is the length of the stimulus in phonemes.
  - *macron* is a binary indicator of whether or not the stimulus contains at least one long vowel, as indicated orthographically by a macron.
  - *enteredResponse* is the wellformedness rating for each stimulus (scale of 1 to 5).
  - *reactionTime* is the reaction time for each rating (seconds).
  - *scoreDictShortVType* is the phonotactic score based on all word types in the dictionary, ignoring all vowel length distinctions.
  - *scoreNBestFixed* is the phonotactic score based on the 3164 most frequent word types, ignoring all vowel length distinctions.
  - *scoreMorphShortVType* is the phonotactic score based on all morph types derived from all words in the dictionary, ignoring vowel length distinctions, assuming that participants are attempting to parse stimuli into morphs.
  - *scoreMorphNBestFixed* is the phonotactic score based on the 1,629 most frequent morph types derived from all words in the dictionary, ignoring vowel length distinctions, assuming that participants are attempting to parse stimuli into morphs.

The phonotactic scores included in the dataset are those that perform best at various stages of the analysis, which we go on to compare in the final stage of the analysis. In the course of the analysis, we also introduce alternative scores, which we label and describe at the time but do not carry through the dataset. For details of the way that phonotactic scores are calculated, see Detailed Methods and Materials, Section 4.2.2-4.2.3, and for details of the different sets of training data used, see Detailed Methods and Materials, Section 4.1.

## 3.2 Overview of participants’ demographics

Figure S7 summarizes the distribution of participants on demographic and linguistic axes, and Table S3 summarizes the number of participants from each group in each stimulus-length condition.

```
# Code for Figure S7: participant demographics for Exp2

participants <- dataExp2 %>%
  group_by(group, workerId) %>%
  summarise(
    maoriList = factor(maoriList[1], levels=0:9),
    maoriProf = factor(maoriProf[1], levels=0:10),
    gender = gender[1],
    age = factor(age[1], levels=c("18-29", "30-39", "40-49", "50-59", "+60"), labels=c("18-29", "30-39", "40-49", "50-59", ">60")),
    education = factor(education[1], levels=c("high", "undergraduate", "graduate"), labels=c("high school", "undergrad", "graduate")),
    place = factor(place[1], levels=c("north", "south"), labels=c("North Island", "South Island")),
    maoriExpo = factor(maoriExpo[1], levels=2:10)
  ) %>%
  ungroup() %>%
  mutate(
    group = fct_relevel(group, c("US", "NMS", "MS"))
  )

FigS7ML <- ggplot(participants, aes(x=maoriList, fill=maoriList)) +
  geom_bar(show.legend=F) +
  stat_count(aes(label=stat(count)), geom="text", vjust=-0.2) +
  facet_grid(. ~ group) +
  labs(x="Basic knowledge of Māori", y="Number of participants") +
  scale_y_continuous(expand=expansion(add=c(0,15))) +
  theme_bw() +
  theme(
    panel.grid = element_blank()
  ) +
  ggtitle("Basic knowledge of Māori, by participant group")

FigS7MP <- ggplot(participants, aes(x=maoriProf, fill=maoriProf)) +
  geom_bar(show.legend=F) +
  stat_count(aes(label=stat(count)), geom="text", vjust=-0.2) +
  facet_grid(. ~ group) +
  labs(x="Māori Proficiency", y="Number of participants") +
  scale_y_continuous(expand=expansion(add=c(0,15))) +
  theme_bw() +
  theme(
    panel.grid = element_blank()
  ) +
  ggtitle("Māori Proficiency, by participant group")

FigS7gender <- ggplot(participants, aes(x=gender, fill=gender)) +
  geom_bar(show.legend=F) +
  stat_count(aes(label=stat(count)), geom="text", vjust=-0.2) +
  facet_grid(. ~ group) +
  labs(x="Gender", y="Number of participants") +
  scale_y_continuous(expand=expansion(add=c(0,20))) +
  theme_bw() +
  theme(
    panel.grid = element_blank()
  ) +
  ggtitle("Gender, by participant group")

FigS7age <- ggplot(participants, aes(x=age, fill=age)) +
  geom_bar(show.legend=F) +
  stat_count(aes(label=stat(count)), geom="text", vjust=-0.2) +
  facet_grid(. ~ group) +
  labs(x="Age group", y="Number of participants") +
  scale_y_continuous(expand=expansion(add=c(0,10))) +
  theme_bw() +
  theme(
    panel.grid = element_blank()
  ) +
  ggtitle("Age group, by participant group")

FigS7edu <- ggplot(participants, aes(x=education, fill=education)) +
  geom_bar(show.legend=F) +
  stat_count(aes(label=stat(count)), geom="text", vjust=-0.2) +
  facet_grid(. ~ group) +
  labs(x="Highest level of education", y="Number of participants") +
  scale_y_continuous(expand=expansion(add=c(0,10))) +
  theme_bw() +
  theme(
    panel.grid = element_blank()
  ) +
  ggtitle("Highest level of education, by participant group")

FigS7place <- ggplot(participants, aes(x=place, fill=place)) +
  geom_bar(show.legend=F) +
  stat_count(aes(label=stat(count)), geom="text", vjust=-0.2) +
  facet_grid(. ~ group) +
  labs(x="Place within NZ", y="Number of participants") +
  scale_x_discrete(na.translate = FALSE) +
  scale_y_continuous(expand=expansion(add=c(0,10))) +
  theme_bw() +
  theme(
    panel.grid = element_blank()
  ) +
  ggtitle("Place within NZ, by participant group")

FigS7ME <- ggplot(participants, aes(x=maoriExpo, fill=maoriExpo)) +
  geom_bar(show.legend=F) +
  stat_count(aes(label=stat(count)), geom="text", vjust=-0.2) +
  facet_grid(. ~ group) +
  labs(x="Level of exposure to Māori", y="Number of participants") +
  scale_x_discrete(na.translate = FALSE) +
  scale_y_continuous(expand=expansion(add=c(0,5))) +
  theme_bw() +
  theme(
    panel.grid = element_blank()
  ) +
  ggtitle("Level of exposure to Māori, by participant group")

ggarrange(FigS7ML, FigS7MP, FigS7gender, FigS7age, FigS7edu, FigS7place, FigS7ME, ncol=1)

remove(participants, FigS7ML, FigS7MP, FigS7gender, FigS7age, FigS7edu, FigS7place, FigS7ME)
```

Figure S7: Overview of participants’ demographics in Exp2.

```
# Code for Table S3: number of participants per length condition, per group

dataExp2 %>%
  distinct(group, workerId, length) %>%
  count(group, length) %>%
  pivot_wider(names_from=group, values_from=n) %>%
  dplyr::select(length, US, NMS, MS) %>%
  display_table(caption="Table S3: Number of participants per length within each group")
```

Table S3: Number of participants per length within each group

| length | US | NMS | MS |
| --- | --- | --- | --- |
| 3 | 16 | 18 | 8 |
| 4 | 13 | 17 | 5 |
| 5 | 16 | 20 | 6 |
| 6 | 16 | 21 | 7 |
| 7 | 14 | 18 | 9 |
| 8 | 19 | 19 | 5 |

## 3.3 Statistical analysis

The analysis of Experiment 2 revolves around the identification of an appropriate source of phonotactic knowledge. We perform this identification through the selection of phonotactic scores that enable an ordinal regression model to best predict participants’ well-formedness ratings (see Detailed Materials and Methods, Sections 4.2.2-4.2.3, for details of phonotactic scoring). The selection process is stepwise, following seven stages, each carrying through and building upon the conclusions of previous stages.

At each stage, we compare a variety of ordinal regression models that are identical apart from the phonotactic scores they employ (and, at one stage, the inclusion of a predictor for macron presence), and we select the model (and corresponding scores) with lowest AIC (see Section 5.2 of the Detailed Materials and Methods Supplement for discussion of AIC). Each model contains a predictor for phonotactic score (centered). In models where the phonotactic score collapses vowel length distinctions, we additionally include a predictor for the presence of visually salient macrons that mark vowel length (binary factor). At different stages, we analyze groups of participants in separate models or in one combined model; whenever groups are combined, we include a predictor for group, interacted with all other predictors in the model. Except for in Monte Carlo analyses, we use mixed-effects ordinal regression models, with random intercepts for participant and stimulus, random slopes by participant for phonotactic score and macron presence (if the phonotactic score collapses vowel length distinctions), and random slopes by stimulus for participant group (if the model combines all groups). In Monte Carlo analyses, we use fixed-effects ordinal regression models for practical reasons; these fixed-effects models differ from the mixed-effects models only in omitting the random effects (see Detailed Materials and Methods, Section 5.3, for more information about the Monte Carlo analyses).

The stages ask the following questions:

1. Is it better to have knowledge of words? If so, tokens or types?
2. To what degree should phonotactic knowledge track vowel length distinctions?
3. How many words do participants need to have knowledge of? *(Monte Carlo analysis)*
4. Is knowledge of Māori borrowings into NZ English sufficient for NMS?
5. Could phonotactic knowledge be underpinned by morphs, i.e. units that could be smaller than words?
6. How many morphs do participants need to have knowledge of, and do participants attempt to parse stimuli into morphs in order to evaluate them phonotactically? *(Monte Carlo analysis)*
7. Once and for all, what is the source of phonotactic knowledge that best explains NMS participants’ well-formedness ratings?

After Stage 7, we consider the role of self-reported exposure to Māori in the best-fitting model of NMS wellformedness ratings.

### 3.3.1 Stage 1: Do we need words?

In the first stage of the analysis, we ask whether participants’ phonotactic knowledge is better assumed to be calculated over unsegmented running speech or over words, and whether calculation over words is better assumed to be over types or tokens. We compare mixed-effects ordinal regression models that combine all groups of participants, which use phonotactic scores based on four different sets of training data:

- **Unsegmented running speech**: the training data are uninterrupted speech streams, without word boundaries (see Detailed Materials and Methods, Section 4.1.1).
- **Word tokens from segmented running speech**: the training data are word tokens taken from running speech corpora (see Detailed Materials and Methods, Section 4.1.1).
- **Word tokens from a dictionary**: the training data are word types from the *Te Aka* dictionary, matched with their token frequencies from running speech corpora (see Detailed Materials and Methods, Section 4.1.2).
  - This represents an intermediate step between word tokens from segmented running speech and word types from a dictionary, to check whether differences between them are due to the assumption of word tokens vs. types, or to differences in the inventory of underlying words.
- **Word types (from a dictionary)**: the training data are word types from the *Te Aka* dictionary (see Detailed Materials and Methods, Section 4.1.2).

The AIC values for the four models are given in Table S4.

The model with phonotactic scores based on word types has lowest AIC. Thus, we only consider word types in word-based phonotactic scores in the following stages.

```
# Code for Table S4: Stage 1 statistical analysis for Exp2

# modelRsUnsegmented <- clmm(
#   enteredResponse ~ 
#     c.(scoreRsUnsegmented) * group + 
#     (1 + c.(scoreRsUnsegmented) | workerId) + (1 + group | word),
#   data=add_scores(dataExp2, "probs/rs_unsegmented.csv", "scoreRsUnsegmented")
# )
# saveRDS(modelRsUnsegmented, file = "dumps/clmm/modelRsUnsegmented.rds")
modelRsUnsegmented <- readRDS("dumps/clmm/modelRsUnsegmented.rds")

# modelRsSegmented <- clmm(
#   enteredResponse ~ 
#     c.(scoreRsSegmented) * group + 
#     (1 + c.(scoreRsSegmented) | workerId) + (1 + group | word),
#   data=add_scores(dataExp2, "probs/rs_segmented.csv", "scoreRsSegmented")
# )
# saveRDS(modelRsSegmented, file = "dumps/clmm/modelRsSegmented.rds")
modelRsSegmented <- readRDS("dumps/clmm/modelRsSegmented.rds")

# modelDictToken <- clmm(
#   enteredResponse ~ 
#     c.(scoreDictToken) * group + 
#     (1 + c.(scoreDictToken) | workerId) + (1 + group | word), 
#   data=add_scores(dataExp2, "probs/dict_tokens.csv", "scoreDictToken")
# )
# saveRDS(modelDictToken, file = "dumps/clmm/modelDictToken.rds")
modelDictToken <- readRDS("dumps/clmm/modelDictToken.rds")

# modelDictType <- clmm(
#   enteredResponse ~ 
#     c.(scoreDictType) * group + 
#     (1 + c.(scoreDictType) | workerId) + (1 + group | word), 
#   data=add_scores(dataExp2, "probs/dict_types.csv", "scoreDictType")
# )
# saveRDS(modelDictType, file = "dumps/clmm/modelDictType.rds")
modelDictType <- readRDS("dumps/clmm/modelDictType.rds")

tibble(
  "Phonotactic score basis"=c("Unsegmented running speech", 
                              "Word tokens from running speech", 
                              "Word tokens from a dictionary", 
                              "Word types (from a dictionary)"),
  "AIC" = c(AIC(modelRsUnsegmented),
            AIC(modelRsSegmented),
            AIC(modelDictToken),
            AIC(modelDictType))
  ) %>%
  display_table(digits=1, highlight=c("AIC"), caption="Table S4: Stage 1 comparison of AIC using phonotactic scores based on different kinds of underlying units")

remove(modelRsUnsegmented, modelRsSegmented, modelDictToken)
```

Table S4: Stage 1 comparison of AIC using phonotactic scores based on different kinds of underlying units

| Phonotactic score basis | AIC |
| --- | --- |
| Unsegmented running speech | 210203.5 |
| Word tokens from running speech | 209007.4 |
| Word tokens from a dictionary | 208806.4 |
| Word types (from a dictionary) | 208613.5 |

### 3.3.2 Stage 2: Should we track vowel length?

In the second stage of the analysis, we ask whether participants’ word-type-based phonotactic knowledge needs to track vowel length distinctions. We compare mixed-effects ordinal regression models that combine all groups of participants, which use phonotactic scores based on word types with three different treatments of vowel length (see Detailed Materials and Methods, Section 4.1.2, for details):

- **All long vowels**: tracks all long vowels separately from short vowels.
- **Long /a:/**: tracks /a:/ separately from /a/, but does not track any other long vowel separately from the corresponding short vowel. (Note that we add a predictor for the visual presence of macrons.)
- **No long vowels**: does not track any long vowel separately from the corresponding short vowel. (Note that we add a predictor for the visual presence of macrons.)

The AIC values for the three models are given in Table S5.

The model with no long vowels has lowest AIC. Thus, we collapse the distinction between long and short vowels in phonotactic scores in the following stages. The phonotactic scores corresponding to this model with no long vowels are preserved in the original dataset as `scoreDictShortVType`.

```
# Code for Table S5: Stage 2 statistical analysis for Exp2

# The model with all long vowels is modelDictType from Stage 1

# modelDictLongAType <- clmm(
#   enteredResponse ~ 
#     (c.(scoreDictLongAType) + macron) * group + 
#     (1 + c.(scoreDictLongAType) + macron | workerId) + (1 + group | word),
#   data=add_scores(dataExp2, "probs/dict-longA_types.csv", "scoreDictLongAType")
# )
# saveRDS(modelDictLongAType, file = "dumps/clmm/modelDictLongAType.rds")
modelDictLongAType <- readRDS("dumps/clmm/modelDictLongAType.rds")

# modelDictShortVType <- clmm(
#   enteredResponse ~ 
#     (c.(scoreDictShortVType) + macron) * group + 
#     (1 + c.(scoreDictShortVType) + macron | workerId) + (1 + group | word), 
#   data=dataExp2
# )
# saveRDS(modelDictShortVType, file = "dumps/clmm/modelDictShortVType.rds")
modelDictShortVType <- readRDS("dumps/clmm/modelDictShortVType.rds")

tibble(
  "Tracking of vowel length"=c("All long vowels", 
                               "Long /a:/", 
                               "No long vowels"),
  "AIC" = c(AIC(modelDictType),
            AIC(modelDictLongAType),
            AIC(modelDictShortVType))
  ) %>%
  display_table(digits=1, highlight=c("AIC"), caption="Table S5: Stage 2 comparison of AIC using phonotactic scores based on word types with different degrees of tracking vowel length")

remove(modelDictType, modelDictLongAType)
```

Table S5: Stage 2 comparison of AIC using phonotactic scores based on word types with different degrees of tracking vowel length

| Tracking of vowel length | AIC |
| --- | --- |
| All long vowels | 208613.5 |
| Long /a:/ | 206543.8 |
| No long vowels | 206336.4 |

#### 3.3.2.1 Summary of Stage 2 model

It is the model with no long vowels that is used for the initial comparison of groups in the main paper. We provide a summary of the model results in Table S6 and Figure 2. (Figure 2a is Figure 2 from the main paper, with slightly different formatting.)

The primary point of interest is that there is no significant interaction between NMS and MS with respect to phonotactic score. This result means that both groups of participants are equally sensitive to phonotactic probability (calculated in this way), and therefore suggests that NMS have a surprisingly high degree of phonotactic knowledge.

```
# Code for Table S6: summary of best model from Stage 2

clm_table(modelDictShortVType, caption="Table S6: Ordinal mixed-effects model summary for well-formedness ratings by participant group. All numeric variables in this model are centered.")
```

Table S6: Ordinal mixed-effects model summary for well-formedness ratings by participant group. All numeric variables in this model are centered.

|  | Parameter | Estimate | Std. Error | \(z\) | \(p\) |  |
| --- | --- | --- | --- | --- | --- | --- |
| Effects | scoreDictShortVType (centered) | 3.002 | 0.179 | 16.748 | <0.001 | \*\*\* |
|  | macron = TRUE | 0.334 | 0.130 | 2.561 | 0.010 | \* |
|  | group = MS | 0.272 | 0.250 | 1.091 | 0.275 |  |
|  | group = US | 0.064 | 0.197 | 0.323 | 0.746 |  |
|  | scoreDictShortVType (centered) × group = MS | -0.030 | 0.305 | -0.099 | 0.921 |  |
|  | scoreDictShortVType (centered) × group = US | -2.391 | 0.242 | -9.863 | <0.001 | \*\*\* |
|  | macron = TRUE × group = MS | -0.375 | 0.220 | -1.703 | 0.088 | . |
|  | macron = TRUE × group = US | 0.571 | 0.176 | 3.235 | 0.001 | \*\* |
| Thresholds | 1|2 | -1.505 | 0.142 |  |  |  |
|  | 2|3 | 0.077 | 0.142 |  |  |  |
|  | 3|4 | 1.230 | 0.142 |  |  |  |
|  | 4|5 | 2.791 | 0.142 |  |  |  |

```
# Code for Figure 2: phonotactic score effect by group, from best model from Stage 2

fig.2a <- clm_plotdat(modelDictShortVType, c("scoreDictShortVType", "group"), xlevels=list(scoreDictShortVType=25), type="mean") %>%
  mutate(
    Group = fct_relevel(group, c("MS", "NMS", "US"))
  ) %>%
  ggplot(., aes(x=scoreDictShortVType, y=pred, fill=Group, color=Group, shape=Group)) +
  geom_ribbon(aes(ymin=lci, ymax=uci), alpha=0.2, color=NA) + 
  geom_line(size=1) +
  scale_color_manual(values = c("black", "red", "orange")) + 
  scale_fill_manual(values = c("black", "red", "orange")) +
  xlab("Phonotactic score") +
  ylab("Predicted mean rating") + 
  theme_classic() + 
  theme(
    legend.position="none", 
    panel.background = element_blank()
  )

fig.2b <- clm_plotdat(modelDictShortVType, c("scoreDictShortVType", "group"), xlevels=list(scoreDictShortVType=25), type="dist") %>%
  mutate(
    Group = fct_relevel(group, c("MS", "NMS", "US"))
  ) %>%
  ggplot(., aes(x=scoreDictShortVType, y=pred, fill=Group, color=Group, shape=Group)) +
  geom_ribbon(aes(ymin=lci, ymax=uci), alpha=0.2, color=NA) + 
  geom_line(size=1) +
  scale_x_continuous(n.breaks=4) +
  scale_color_manual(values = c("black", "red", "orange")) + 
  scale_fill_manual(values = c("black", "red", "orange")) +
  xlab("Phonotactic score") +
  ylab("Probability of rating") +     
  facet_grid(. ~ response, labeller=as_labeller(function(x) str_c("Rating: ", x))) +
  ylim(0, 1) +
  theme_bw() + 
  theme(
    legend.position="right", 
    panel.background = element_blank()
  )

ggarrange(fig.2a, fig.2b, ncol=2, labels=c("a.", "b."), widths=c(1,5))

remove(fig.2a, fig.2b)
```

Figure 2: Interaction between phonotactic scores and participant groups in the best model from Stage 2. Figure 2a (left) shows predicted mean ratings; Figure 2b (right) shows predicted distributions over ratings (facets).

### 3.3.3 Stage 3: How many words do we need?

In the third stage of the analysis, we ask how many word types underpin the phonotactic knowledge evidenced by NMS and MS, in order to evaluate (i) whether there are signs of differences between them, and (ii) if NMS really require a proto-lexicon consisting of over 18,000 words. We conduct Monte Carlo analyses that compare fixed-effects ordinal regression models separately for each participant group, using phonotactic scores based on different numbers of word types without vowel length distinctions. We consider three different ways of sampling a *vocabulary* consisting of a fixed number of words (see Detailed Materials and Methods, Section 5.3, for details):

- **Unweighted**: samples types uniformly at random, regardless of frequency.
- **Frequency-weighted**: samples types proportional to their frequency.
- **N-highest-frequency**: samples only the highest-frequency types.

The results are visualized in Figure 3 (also included in the main paper). For NMS, models suffer no substantial increase in AIC with the use of phonotactic scores based on a subset of approximately 3,000 high-frequency words. For MS, optimal performance is seen with phonotactic knowledge based on a larger subset of words.

The Monte Carlo analysis provides us with an idea of the best vocabulary size, but this idea is based on a thousand different models, each with a different vocabulary of that size. Thus, the analysis does not provide us with a fixed vocabulary that can be assumed in a single model, for comparison with other models in a mixed-effects setting. To obtain such a fixed vocabulary, we repeat the fixed-effects ordinal regression analysis using phonotactic scores based on all words with raw frequency greater than or equal to *k*, for values of *k* from 0 to 8. This is essentially a version of the *N-highest-frequency* sampling scheme that admits no randomness.

The results of the fixed-vocabulary analysis are presented in Table S7. The lowest AIC comes from the use of phonotactic scores based on the 3,164 words with raw frequency greater than or equal to 5. Thus, we use these phonotactic scores for comparison in the following stages. These phonotactic scores are preserved in the original dataset as `scoreNBestFixed`.

```
# Stage 3 Monte Carlo analysis for Exp2 -- words

# Note: since we purchased the set of dictionary types, we cannot make them freely available here; thus, this section of code cannot be re-run.

# Set up file structure
dir.create("EXCLUDED/MC_words")
for (scheme in c("unweighted", "freq-weighted", "N-highest-freq")) {
  dir.create(paste("EXCLUDED/MC_words", scheme, sep="/"))
  for (subdir in c("samples", "auxiliaries", "phonotactic-models", "probs")) {
    dir.create(paste("EXCLUDED/MC_words", scheme, subdir, sep="/"))
    for (vocabsize in seq(from=1000, to=18000, by=1000)) {
      dir.create(paste("EXCLUDED/MC_words", scheme, subdir, vocabsize, sep="/"))
    }
  }
}
remove(subdir)

# Step 1: get samples
dictShortVFreq <- read.delim("EXCLUDED/train/dict-shortvowels_freq.txt", sep="\t", header=TRUE, stringsAsFactors=FALSE); dictShortVFreq$word = trimws(gsub("(.)", "\\1 ", dictShortVFreq$word))
set.seed(1234)

for (vocabsize in seq(from=1000, to=18000, by=1000)) {
  for (samplenum in 1:1000) {
    
    # Unweighted sample
    sample(dictShortVFreq$word, vocabsize, replace=FALSE) %>%
      write.table(., 
                  paste("EXCLUDED/MC_words/unweighted/samples", 
                        vocabsize, 
                        paste0("sample_", samplenum, ".txt"), 
                        sep="/"), 
                  row.names=FALSE, col.names=FALSE, quote=FALSE)
    
    # Frequency-weighted sample
    sample(dictShortVFreq$word, vocabsize, replace=FALSE, prob=dictShortVFreq$tokens) %>%
      write.table(., 
                  paste("EXCLUDED/MC_words/freq-weighted/samples", 
                        vocabsize, 
                        paste0("sample_", samplenum, ".txt"), 
                        sep="/"), 
                  row.names=FALSE, col.names=FALSE, quote=FALSE)
    
    # N-highest-frequency sample
    dictShortVFreq <- dictShortVFreq[order(runif(nrow(dictShortVFreq))),]
    dictShortVFreq <- dictShortVFreq[order(dictShortVFreq$tokens, decreasing=TRUE),] 
    dictShortVFreq[1:vocabsize, "word"] %>%
      write.table(., 
                  paste("EXCLUDED/MC_words/N-highest-freq/samples", 
                        vocabsize, 
                        paste0("sample_", samplenum, ".txt"), 
                        sep="/"), 
                  row.names=FALSE, col.names=FALSE, quote=FALSE)
  }    
}
remove(dictShortVFreq)

# Step 2: calculate phonotactic probabilities, using the score-unparsed.sh shell script
setwd("scripts")
for (scheme in c("unweighted", "freq-weighted", "N-highest-freq")) {
  for (vocabsize in seq(from=1000, to=18000, by=1000)) {
    for (samplenum in 1:1000) {
      shellCommand <- paste0(
        "sh score-unparsed.sh", 
        " ../EXCLUDED/MC_words/", scheme, "/samples/", vocabsize, "/sample_", samplenum, ".txt",
        " stimuli-shortvowels.txt",
        " ../EXCLUDED/MC_words/", scheme, "/phonotactic-models/", vocabsize,
        " ../EXCLUDED/MC_words/", scheme, "/auxiliaries/", vocabsize,
        " ../EXCLUDED/MC_words/", scheme, "/probs/", vocabsize
      )
      run_shell(shellCommand)
    }
  }
}
setwd("..")
remove(shellCommand)

# Step 3: compute fixed-effects ordinal regression models and save the AIC values
AIC_MC_words = data.frame(
  group = rep(c("NMS", "MS"), each=3*18*1000),
  scheme = rep(rep(c("unweighted", "freq-weighted", "N-highest-freq"), each=18*1000), 2),
  vocabsize = rep(rep(seq(from=1000, to=18000, by=1000), each=1000), 2*3),
  samplenum = rep(1:1000, 2*3*18),
  aic = rep(NA, 2*3*18*1000)
)
for (group in c("NMS", "MS")) {
  groupdat <- dataExp2[dataExp2$group==group,]
  for (scheme in c("unweighted", "freq-weighted", "N-highest-freq")) {
    for (vocabsize in seq(from=1000, to=18000, by=1000)) {
      aic_values = rep(NA, 1000)
      for (samplenum in 1:1000) {
        mod <- clm(
          enteredResponse ~ c.(score) + macron,
          data=add_scores(groupdat, paste0("EXCLUDED/MC_words/", scheme, "/probs/", vocabsize, "/sample_", samplenum, ".csv"), "score")
        )
        aic_values[samplenum] <- AIC(mod)
      }
      AIC_MC_words[AIC_MC_words$group==group & AIC_MC_words$scheme==scheme & AIC_MC_words$vocabsize==vocabsize, "aic"] <- aic_values
    }
  }
}

write.table(AIC_MC_words, file="dumps/monte-carlo/AIC_MC_words.txt", row.names=FALSE, quote=FALSE, sep="\t")
remove(AIC_MC_words, aic_values, mod, samplenum, vocabsize, scheme, groupdat, group)
```

```
# Code for Figure 3: Stage 3 Monte Carlo analysis for Exp2 -- words

fig3dat <- read.delim("dumps/monte-carlo/AIC_MC_words.txt", sep="\t", header=TRUE) %>%
  group_by(group, scheme, vocabsize) %>%
  summarise(
    mean.AIC = mean(aic),
    lower.AIC = quantile(aic, probs=c(0.025)),
    upper.AIC = quantile(aic, probs=c(0.975))
  ) %>%
  ungroup() %>%
  mutate(
    group = fct_relevel(group, "NMS")
  )

# Get the AIC values for the full dictionary for each group, in a fixed-effects setting
# These are preserved as a baseline for the morph-based Monte Carlo analysis to come
fullDictAICNMS <- clm(
  enteredResponse ~ c.(scoreDictShortVType) + macron,
  data=dataExp2[dataExp2$group=="NMS",]
) %>%
  AIC(.)
fullDictAICMS <- clm(
  enteredResponse ~ c.(scoreDictShortVType) + macron,
  data=dataExp2[dataExp2$group=="MS",]
) %>%
  AIC(.)

fig3A <- ggplot(fig3dat[fig3dat$group=="NMS",], aes(x=vocabsize, y=mean.AIC, ymin=lower.AIC, ymax=upper.AIC, color=scheme, fill=scheme)) +
  geom_errorbar(position=position_dodge(width=200), size=1) +
  geom_line(position=position_dodge(width=200), size=1, alpha=0.5) +
  geom_hline(aes(yintercept=fullDictAICNMS, linetype="All words (18.7k)"), color="black", size=1) +   
  geom_point(shape=21, color="black", position=position_dodge(width=200), size=3) +
  labs(
    title="Word-based Monte Carlo analyses with NMS participants",
    x="Vocabulary size", 
    y="AIC score"
  ) + 
  scale_x_continuous(
    breaks=seq(from=1000, to=18000, by=1000),
    labels=function(b){return(paste0(b/1000,"k"))}
    ) + 
  scale_color_discrete(name="Sampling", breaks=c("unweighted", "freq-weighted", "N-highest-freq")) +
  scale_fill_discrete(name="Sampling", breaks=c("unweighted", "freq-weighted", "N-highest-freq")) +
  scale_linetype_manual(name=NULL, values=c("All words (18.7k)"="dashed")) +
  guides(color=guide_legend(order=1), fill=guide_legend(order=1), linetype=guide_legend(order=2)) +
  theme_classic() + 
  theme(
    plot.title=element_text(hjust=0.5, size=15),
    legend.text=element_text(size=10),
    panel.background = element_blank()
  )

fig3B <- ggplot(fig3dat[fig3dat$group=="MS",], aes(x=vocabsize, y=mean.AIC, ymin=lower.AIC, ymax=upper.AIC, color=scheme, fill=scheme)) +
  geom_errorbar(position=position_dodge(width=200), size=1) +
  geom_line(position=position_dodge(width=200), size=1, alpha=0.5) +
  geom_hline(aes(yintercept=fullDictAICMS, linetype="All words (18.7k)"), color="black", size=1) +   
  geom_point(shape=21, color="black", position=position_dodge(width=200), size=3) +
  labs(
    title="Word-based Monte Carlo analyses with MS participants",
    x="Vocabulary size", 
    y="AIC score"
  ) + 
  scale_x_continuous(
    breaks=seq(from=1000, to=18000, by=1000),
    labels=function(b){return(paste0(b/1000,"k"))}
    ) +  
  scale_color_discrete(name="Sampling", breaks=c("unweighted", "freq-weighted", "N-highest-freq")) +
  scale_fill_discrete(name="Sampling", breaks=c("unweighted", "freq-weighted", "N-highest-freq")) +
  scale_linetype_manual(name=NULL, values=c("All words (18.7k)"="dashed")) +    
  guides(color=guide_legend(order=1), fill=guide_legend(order=1), linetype=guide_legend(order=2)) +  
  theme_classic() + 
  theme(
    plot.title=element_text(hjust=0.5, size=15),
    legend.text=element_text(size=10),
    panel.background = element_blank()
  )

ggarrange(fig3A, fig3B, ncol=1)

remove(fig3A, fig3B, fig3dat)
```

Figure 3: Monte Carlo analyses with 1,000 random samples over 18 vocabulary sizes. Error bars represent 95% bootstrap percentile intervals.

```
# Code for Table S7: Stage 3 statistical comparison of fixed vocabularies for Exp2

# Get AIC from clm for different fixed subsets
path <- "probs/NBestFixed-word-shortvowels/"
filenames <- list.files(path)
NBestFixedAICs <- data.frame(
  group = rep(c("NMS", "MS"), each=length(filenames)),
  freq = as.integer(rep(str_extract(filenames, "(?<=Freq)\\d+(?=_)"), 2)),
  N = as.integer(rep(str_extract(filenames, "(?<=_)\\d+(?=\\.csv)"), 2)),
  aic = rep(NA, length(filenames)*2)
)
for (group in c("NMS", "MS")) {
  groupdat <- dataExp2[dataExp2$group==group,]
  for (filename in filenames) {
    freq <- str_extract(filename, "(?<=Freq)\\d+(?=_)")
    N <- str_extract(filename, "(?<=_)\\d+(?=\\.csv)")
    aic <- clm(
      enteredResponse ~ score + macron,
      data=add_scores(groupdat, paste0(path, filename), "score")
    ) %>%
      AIC(.)
    NBestFixedAICs[NBestFixedAICs$group==group & NBestFixedAICs$freq==freq & NBestFixedAICs$N==N, "aic"] <- aic
  }
}
remove(aic, freq, N, filename, groupdat, group, filenames, path)

NBestFixedAICs %>%
  pivot_wider(names_from=group, values_from=aic) %>%
  rename(
    "Min. freq" = "freq",
    "Vocab size" = "N",
    "AIC (NMS)" = "NMS",
    "AIC (MS)" = "MS"
  ) %>%
  display_table(digits=1, highlight=c("AIC (NMS)", "AIC (MS)"), caption="Table S7: AIC scores for NMS and MS participants, using phonotactic scores based on fixed vocabularies of all words with frequency greater than or equal to a given minimum")

remove(NBestFixedAICs)
```

Table S7: AIC scores for NMS and MS participants, using phonotactic scores based on fixed vocabularies of all words with frequency greater than or equal to a given minimum

| Min. freq | Vocab size | AIC (NMS) | AIC (MS) |
| --- | --- | --- | --- |
| 0 | 18703 | 103751.8 | 36671.1 |
| 1 | 5821 | 103837.8 | 36666.4 |
| 2 | 4526 | 103770.0 | 36669.2 |
| 3 | 3879 | 103760.8 | 36652.0 |
| 4 | 3470 | 103782.6 | 36645.5 |
| 5 | 3164 | 103703.1 | 36652.1 |
| 6 | 2910 | 103804.2 | 36660.9 |
| 7 | 2707 | 103813.8 | 36667.5 |
| 8 | 2539 | 103915.8 | 36686.9 |

### 3.3.4 Stage 4: Are Māori borrowings sufficient?

The Monte Carlo analysis demonstrated that we can provide an adequate explanation of the ratings of NMS participants by assuming that they form phonotactic models over a subset of the lexicon (ignoring vowel length). Here, we test whether the same is true for a smaller subset consisting of Māori words that have been borrowed into NZ English.

We compare two mixed-effects ordinal regression models over NMS only, one of which uses phonotactic scores based on the 3,164 highest-frequency words in the *Te Aka* Māori dictionary, and the other of which uses phonotactic scores based on 1,760 Māori borrowings and placenames in New Zealand English. In keeping with the conclusions of stage two, both sources have vowel length distinctions collapsed.

The results of the comparison are presented in Table S8. As can be seen, the model with phonotactic scores based on words from the Māori dictionary has lowest AIC. Thus, we continue with the following stages assuming that NMS have implicit knowledge of high-frequency types from the dictionary, and not just of borrowings in New Zealand English.

Table S8 shows that the performance of NMS on the well-formedness ratings task cannot be boiled down to knowledge of words in NZ English only. This conclusion is further reinforced by the results of a mixed-effects regression model across all participant groups that includes phonotactic scores from both sources (Table S9). In this regression, both the dictionary-based and the borrowings-based phonotactic scores have significant main effects (i.e. for NMS participants). Thus, while NMS participants may recruit phonotactic knowledge from both sources, we have no evidence that they recruit phonotactic knowledge from one to the exclusion of the other.

```
# Code for Table S8: Stage 4 statistical analysis for Exp2

# modelNBestFixedNMS <- clmm(
#   enteredResponse ~
#     c.(scoreNBestFixed) + macron +
#     (1 + c.(scoreNBestFixed) + macron | workerId) + (1 | word),
#   data=dataExp2[dataExp2$group=="NMS",]
# )
# saveRDS(modelNBestFixedNMS, file = "dumps/clmm/modelNBestFixedNMS.rds")
modelNBestFixedNMS <- readRDS("dumps/clmm/modelNBestFixedNMS.rds")

# modelBorrowingsNMS <- clmm(
#   enteredResponse ~
#     c.(scoreBorrowings) + macron +
#     (1 + c.(scoreBorrowings) + macron | workerId) + (1 | word),
#   data=add_scores(dataExp2[dataExp2$group=="NMS",], "probs/borrowings-shortvowels_types.csv", "scoreBorrowings")
# )
# saveRDS(modelBorrowingsNMS, file = "dumps/clmm/modelBorrowingsNMS.rds")
modelBorrowingsNMS <- readRDS("dumps/clmm/modelBorrowingsNMS.rds")

tibble(
  "Source of words"=c("Dictionary subset",
                      "Borrowings"),
  "AIC" = c(AIC(modelNBestFixedNMS),
            AIC(modelBorrowingsNMS))
  ) %>%
  display_table(digits=1, highlight=c("AIC"), caption="Table S8: Stage 4 comparison of AIC using phonotactic scores based on the 3,164 highest-frequency word types from the Te Aka Māori dictionary and 1,760 Māori borrowings in NZ English (ignoring vowel length distinctions)")

remove(modelBorrowingsNMS)
```

Table S8: Stage 4 comparison of AIC using phonotactic scores based on the 3,164 highest-frequency word types from the Te Aka Māori dictionary and 1,760 Māori borrowings in NZ English (ignoring vowel length distinctions)

| Source of words | AIC |
| --- | --- |
| Dictionary subset | 93999.2 |
| Borrowings | 94067.3 |

```
# Code for Table S9: Statistical analysis combining phonotactic scores from Māori dictionary and borrowings

# modelBorrowingsAll <- clmm(
#   enteredResponse ~
#     (c.(scoreNBestFixed) + c.(scoreBorrowings) + macron) * group +
#     (1 + macron | workerId) + (0 + c.(scoreNBestFixed) + c.(scoreBorrowings) | workerId) + (1 + group | word),
#   data=add_scores(dataExp2, "probs/borrowings-shortvowels_types.csv", "scoreBorrowings")
# ) # Note the use of uncorrelated random slopes by workerId, due to convergence issues
# saveRDS(modelBorrowingsAll, file = "dumps/clmm/modelBorrowingsAll.rds")
modelBorrowingsAll <- readRDS("dumps/clmm/modelBorrowingsAll.rds")

clm_table(modelBorrowingsAll, caption="Table S9: Ordinal mixed-effects model of well-formedness ratings including separate phonotactic scores derived from Te Aka and from the dictionary of Māori words in New Zealand English")

remove(modelBorrowingsAll)
```

Table S9: Ordinal mixed-effects model of well-formedness ratings including separate phonotactic scores derived from Te Aka and from the dictionary of Māori words in New Zealand English

|  | Parameter | Estimate | Std. Error | \(z\) | \(p\) |  |
| --- | --- | --- | --- | --- | --- | --- |
| Effects | scoreNBestFixed (centered) | 1.718 | 0.189 | 9.083 | <0.001 | \*\*\* |
|  | scoreBorrowings (centered) | 1.639 | 0.201 | 8.166 | <0.001 | \*\*\* |
|  | macron = TRUE | 0.255 | 0.128 | 1.991 | 0.046 | \* |
|  | group = MS | 0.265 | 0.249 | 1.066 | 0.286 |  |
|  | group = US | -0.002 | 0.195 | -0.011 | 0.991 |  |
|  | scoreNBestFixed (centered) × group = MS | -0.144 | 0.255 | -0.566 | 0.571 |  |
|  | scoreNBestFixed (centered) × group = US | -1.616 | 0.225 | -7.168 | <0.001 | \*\*\* |
|  | scoreBorrowings (centered) × group = MS | -0.105 | 0.266 | -0.394 | 0.694 |  |
|  | scoreBorrowings (centered) × group = US | -1.080 | 0.237 | -4.554 | <0.001 | \*\*\* |
|  | macron = TRUE × group = MS | -0.364 | 0.218 | -1.674 | 0.094 | . |
|  | macron = TRUE × group = US | 0.645 | 0.174 | 3.712 | <0.001 | \*\*\* |
| Thresholds | 1|2 | -1.580 | 0.141 |  |  |  |
|  | 2|3 | 0.003 | 0.141 |  |  |  |
|  | 3|4 | 1.159 | 0.141 |  |  |  |
|  | 4|5 | 2.726 | 0.141 |  |  |  |

### 3.3.5 Stage 5: Are morphs better?

In the fifth stage of the analysis, we ask whether participants’ phonotactic knowledge could be underpinned not by words, but by morphs that could be smaller than words, since morphs occur across words and thus should be easier to implicitly acquire from streams of speech. We compare three mixed-effects ordinal regression models that combine all groups of participants, using phonotactic scores based on three different kinds of units:

- **Word types**: the training data consist of all of the word types in the dictionary, without vowel length distinctions (as in the result of stage two).
- **Morph types**: the training data consist of morph types derived from all of the words in the dictionary, without vowel length distinctions (see Detailed Materials and Methods, Section 4.1.3, for details of morphs). Scores assume that participants are attempting to parse stimuli into morphs (see Detailed Materials and Methods, Section 4.2.3).
- **Morph tokens**: the training data consist of morph types derived from all of the words in the dictionary, without vowel length distinctions, weighted by their frequency in corpora (see Detailed Materials and Methods, Section 4.1.3, for details of morphs). Scores assume that participants are attempting to parse stimuli into morphs (see Detailed Materials and Methods, Section 4.2.3).

The AIC values for the three models are given in Table S10.

The model with phonotactic scores based on morph types has lowest AIC. Thus, we only consider morph types for morph-based scores in the following stages. The phonotactic scores corresponding to this model with no long vowels are preserved in the original dataset as `scoreMorphShortVType`.

In the morph-based model, we still do not observe a significant difference in the sensitivities of NMS and MS participants to phonotactic scores, as shown in the model summary in Table S11.

```
# Code for Table S10: Stage 5 statistical analysis for Exp2

# The model based on word types is modelDictShortVType from Stage 2

# modelMorphShortVType <- clmm(
#   enteredResponse ~ 
#     (c.(scoreMorphShortVType) + macron) * group + 
#     (1 + c.(scoreMorphShortVType) + macron | workerId) + (1 + group | word),
#   data=dataExp2
# )
# saveRDS(modelMorphShortVType, file = "dumps/clmm/modelMorphShortVType.rds")
modelMorphShortVType <- readRDS("dumps/clmm/modelMorphShortVType.rds")

# modelMorphShortVToken <- clmm(
#   enteredResponse ~ 
#     (c.(scoreMorphShortVToken) + macron) * group + 
#     (1 + c.(scoreMorphShortVToken) + macron | workerId) + (1 + group | word),
#   data=add_scores(dataExp2, "probs/morphs-shortvowels_tokens_parsed.csv", "scoreMorphShortVToken")
# )
# saveRDS(modelMorphShortVToken, file = "dumps/clmm/modelMorphShortVToken.rds")
modelMorphShortVToken <- readRDS("dumps/clmm/modelMorphShortVToken.rds")

tibble(
  "Phonotactic score basis"=c("Word types", 
                              "Morph types", 
                              "Morph tokens"),
  "AIC" = c(AIC(modelDictShortVType),
            AIC(modelMorphShortVType),
            AIC(modelMorphShortVToken))
  ) %>%
  display_table(digits=1, highlight=c("AIC"), caption="Table S10: Stage 5 comparison of AIC using phonotactic scores based on word types, morph types, and morph tokens (ignoring vowel length distinctions, and assuming participants are attempting to parse stimuli into morphs for morph-based scores)")

remove(modelMorphShortVToken)
```

Table S10: Stage 5 comparison of AIC using phonotactic scores based on word types, morph types, and morph tokens (ignoring vowel length distinctions, and assuming participants are attempting to parse stimuli into morphs for morph-based scores)

| Phonotactic score basis | AIC |
| --- | --- |
| Word types | 206336.4 |
| Morph types | 205994.1 |
| Morph tokens | 207944.8 |

```
# Code for Table S11: summary of best model from Stage 5

clm_table(modelMorphShortVType, caption="Table S11: Ordinal mixed-effects model summary for well-formedness ratings by participant group, assuming morph-based phonotactic scores (and parsing of stimuli into morphs). All numeric variables in this model are centered.")
```

Table S11: Ordinal mixed-effects model summary for well-formedness ratings by participant group, assuming morph-based phonotactic scores (and parsing of stimuli into morphs). All numeric variables in this model are centered.

|  | Parameter | Estimate | Std. Error | \(z\) | \(p\) |  |
| --- | --- | --- | --- | --- | --- | --- |
| Effects | scoreMorphShortVType (centered) | 4.918 | 0.288 | 17.057 | <0.001 | \*\*\* |
|  | macron = TRUE | 0.288 | 0.129 | 2.235 | 0.025 | \* |
|  | group = MS | 0.259 | 0.250 | 1.037 | 0.300 |  |
|  | group = US | 0.020 | 0.196 | 0.103 | 0.918 |  |
|  | scoreMorphShortVType (centered) × group = MS | -0.463 | 0.503 | -0.919 | 0.358 |  |
|  | scoreMorphShortVType (centered) × group = US | -4.103 | 0.396 | -10.372 | <0.001 | \*\*\* |
|  | macron = TRUE × group = MS | -0.344 | 0.220 | -1.562 | 0.118 |  |
|  | macron = TRUE × group = US | 0.626 | 0.175 | 3.573 | <0.001 | \*\*\* |
| Thresholds | 1|2 | -1.547 | 0.141 |  |  |  |
|  | 2|3 | 0.039 | 0.141 |  |  |  |
|  | 3|4 | 1.196 | 0.141 |  |  |  |
|  | 4|5 | 2.762 | 0.141 |  |  |  |

### 3.3.6 Stage 6: How many morphs do we need?

In the sixth stage of the analysis, we ask how many morph types underpin the phonotactic knowledge evidenced by NMS and MS, and whether participants in these groups attempt to parse the stimuli into morphs when they evaluate them phonotactically. As in Stage 3, our investigation has two key motivations: (i) to identify signs of differences between NMS and MS; and (ii) to further reduce the size of the proto-lexicon required to best explain NMS’ phonotactic knowledge.

We conduct Monte Carlo analyses that compare fixed-effects ordinal regression models separately for each participant group, using phonotactic scores based on different numbers of morph types without vowel length distinctions. As in Stage 3, we consider three different ways of sampling a *morph set* consisting of a fixed number of morphs (see Detailed Materials and Methods, Section 5.3, for details):

- **Unweighted**: samples morph types uniformly at random, regardless of frequency.
- **Frequency-weighted**: samples morph types proportional to their frequency.
- **N-highest-frequency**: samples only the highest-frequency morph types.

For each sample, we also consider two different morph-parsing approaches to stimuli:

- **Unparsed**: participants do not attempt to parse the nonword stimuli into morphs, instead treating them as consisting of a single morph (see Detailed Materials and Methods, Section 4.2.1, for details).
- **Parsed**: participants attempt to parse the nonword stimuli into morphs (see Detailed Materials and Methods, section 4.2.2, for details).  
  *(Note: this is the approach assumed in Stage 5.)*

The results are visualized in Figure 4 (also included in the main paper). For NMS, lowest AIC is seen in models that assume that participants are parsing stimuli into morphs, while for MS, lowest AIC is seen in models that assume that participants are *not* parsing stimuli into morphs. For NMS, parsing-based models suffer no substantial increase in AIC with the use of phonotactic scores based on a subset of approximately 1,500 high-frequency morphs.

As in Stage 3, we also consider fixed morph sets. We repeat the fixed-effects ordinal regression analysis using phonotactic scores based on all morphs with raw frequency greater than or equal to *k*, for values of *k* from 0 to 6. This is essentially a version of the *N-highest-frequency* sampling scheme that admits no randomness.

The results of the fixed-vocabulary analysis are presented in Table S12. For NMS, the lowest AIC comes from the use of parsing-based phonotactic scores based on the 1,629 morphs with raw frequency greater than or equal to 2, while for MS, the lowest AIC comes from the use of *non*-parsing-based phonotactic scores based on morphs derived from *all* words in the dictionary. We carry forward the parsing-based scores based on the highest-frequency 1,629 morphs to the final stage of comparison for NMS; they are also preserved in the original dataset as `scoreMorphNBestFixed`.

What does it mean to have a proto-lexicon of this size? To get some insight, we consider the average number of morphs an NMS participant would have to learn.

Averaged across all of our participants, the best NMS proto-lexicon contained approximately 1,600 morphs. We have no way of knowing the average age of our participants, since we did not collect precise age measurements, but, given the distribution in Figure S7, we can be quite sure that it is at least 20 years old. Thus, if we make the simplifying assumption that the proto-lexicon grows at a constant rate, our results imply that the average NMS learns 80 or fewer morphs per year.

```
# Stage 3 Monte Carlo analysis for Exp2 -- morphs

# Note: this section of the code can be re-run, but it will take a long time (~24hrs) and create a lot of files in the directory denoted by the variable "container".
# Use of the scripts requires SRILM to be installed.
# On a Windows machine, use of the scripts additionally requires a POSIX shell (e.g. cygwin) to be installed, with the corresponding env set to the environment variable R_SHELL.

container <- "EXCLUDED" # Set to "." for current directory

# Set up file structure
dir.create(paste(container, "MC_morphs", sep="/"))
for (scheme in c("unweighted", "freq-weighted", "N-highest-freq")) {
  dir.create(paste(container, "MC_morphs", scheme, sep="/"))
  dir.create(paste(container, "MC_morphs", scheme, "samples", sep="/"))
  for (parsing in c("parsed", "unparsed")) {
    dir.create(paste(container, "MC_morphs", scheme, parsing, sep="/"))
    for (subdir in c("auxiliaries", "phonotactic-models", "probs")) {
      dir.create(paste(container, "MC_morphs", scheme, parsing, subdir, sep="/"))
      for (morphsetsize in seq(from=500, to=3500, by=500)) {
        dir.create(paste(container, "MC_morphs", scheme, "samples", morphsetsize, sep="/"))
        dir.create(paste(container, "MC_morphs", scheme, parsing, subdir, morphsetsize, sep="/"))
      }
    }
  }
}
remove(subdir)

# Step 1: get samples
morphShortVFreq <- read.delim("scripts/train/morphs-shortvowels_weighted-tokens-smoothed.txt", sep="\t", header=FALSE, stringsAsFactors=FALSE) %>%
  rename("tokens"="V1", "morph"="V2") %>%
  left_join(
    read.delim("scripts/train/morphs-shortvowels_weighted-tokens-raw.txt", sep="\t", header=FALSE, stringsAsFactors=FALSE) %>%
      rename("tokens.raw"="V1", "morph"="V2"),
    by="morph"
  )
set.seed(1234)

for (morphsetsize in seq(from=500, to=3500, by=500)) {
  for (samplenum in 1:1000) {
    
    # Unweighted sample
    sample(morphShortVFreq$morph, morphsetsize, replace=FALSE) %>%
      write.table(., 
                  paste(container, "MC_morphs/unweighted/samples", 
                        morphsetsize, 
                        paste0("sample_", samplenum, ".txt"), 
                        sep="/"), 
                  row.names=FALSE, col.names=FALSE, quote=FALSE)
    
    # Frequency-weighted sample
    sample(morphShortVFreq$morph, morphsetsize, replace=FALSE, prob=morphShortVFreq$tokens) %>%
      write.table(., 
                  paste(container, "MC_morphs/freq-weighted/samples", 
                        morphsetsize, 
                        paste0("sample_", samplenum, ".txt"), 
                        sep="/"), 
                  row.names=FALSE, col.names=FALSE, quote=FALSE)
    
    # N-highest-frequency sample
    morphShortVFreq <- morphShortVFreq[order(runif(nrow(morphShortVFreq))),]
    morphShortVFreq <- morphShortVFreq[order(morphShortVFreq$tokens.raw, decreasing=TRUE),] 
    morphShortVFreq[1:morphsetsize, "morph"] %>%
      write.table(., 
                  paste(container, "MC_morphs/N-highest-freq/samples", 
                        morphsetsize, 
                        paste0("sample_", samplenum, ".txt"), 
                        sep="/"), 
                  row.names=FALSE, col.names=FALSE, quote=FALSE)
  }    
}
remove(morphShortVFreq)

# Step 2: calculate phonotactic probabilities, using the score-unparsed.sh and score-parsed.sh shell scripts
setwd("scripts")
for (scheme in c("unweighted", "freq-weighted", "N-highest-freq")) {
  for (parsing in c("parsed", "unparsed")) {
    if (parsing=="parsed") {
      stimsuffix = "_parsed"
    } else {
      stimsuffix = ""
    }
    for (morphsetsize in seq(from=500, to=3500, by=500)) {
      for (samplenum in 1:1000) {
        shellCommand <- paste0(
          "sh score-", parsing ,".sh", 
          " ../", container, "/MC_morphs/", scheme, "/samples/", morphsetsize, "/sample_", samplenum, ".txt",
          " stimuli-shortvowels", stimsuffix, ".txt",
          " ../", container, "/MC_morphs/", scheme, "/", parsing, "/phonotactic-models/", morphsetsize,
          " ../", container, "/MC_morphs/", scheme, "/", parsing, "/auxiliaries/", morphsetsize,
          " ../", container, "/MC_morphs/", scheme, "/", parsing, "/probs/", morphsetsize
        )
        run_shell(shellCommand)
      }
    }
  }
}
setwd("..")
remove(shellCommand, stimsuffix)

# Step 3: compute fixed-effects ordinal regression models and save the AIC values
AIC_MC_morphs = data.frame(
  group = rep(c("NMS", "MS"), each=3*2*7*1000),
  scheme = rep(rep(c("unweighted", "freq-weighted", "N-highest-freq"), each=2*7*1000), 2),
  parsing = rep(rep(c("parsed", "unparsed"), each=7*1000), 2*3),
  morphsetsize = rep(rep(seq(from=500, to=3500, by=500), each=1000), 2*3*2),
  samplenum = rep(1:1000, 2*3*2*7),
  aic = rep(NA, 2*3*2*7*1000)
)
for (group in c("NMS", "MS")) {
  groupdat <- dataExp2[dataExp2$group==group,]
  for (scheme in c("unweighted", "freq-weighted", "N-highest-freq")) {
    for (parsing in c("parsed", "unparsed")) {
      for (morphsetsize in seq(from=500, to=3500, by=500)) {
        aic_values = rep(NA, 1000)
        for (samplenum in 1:1000) {
          mod <- clm(
            enteredResponse ~ c.(score) + macron,
            data=add_scores(groupdat, paste0(container, "/MC_morphs/", scheme, "/", parsing, "/probs/", morphsetsize, "/sample_", samplenum, ".csv"), "score")
          )
          aic_values[samplenum] <- AIC(mod)
        }
        AIC_MC_morphs[AIC_MC_morphs$group==group & AIC_MC_morphs$scheme==scheme & AIC_MC_morphs$parsing==parsing & AIC_MC_morphs$morphsetsize==morphsetsize, "aic"] <- aic_values
      }
    }
  }
}

write.table(AIC_MC_morphs, file="dumps/monte-carlo/AIC_MC_morphs.txt", row.names=FALSE, quote=FALSE, sep="\t")
remove(AIC_MC_morphs, aic_values, mod, samplenum, morphsetsize, parsing, scheme, groupdat, group, container)
```

```
# Code for Figure 4: Stage 6 Monte Carlo analysis for Exp2 -- morphs

fig4dat <- read.delim("dumps/monte-carlo/AIC_MC_morphs.txt", sep="\t", header=TRUE) %>%
  group_by(group, scheme, parsing, morphsetsize) %>%
  summarise(
    mean.AIC = mean(aic),
    lower.AIC = quantile(aic, probs=c(0.025)),
    upper.AIC = quantile(aic, probs=c(0.975))
  ) %>%
  ungroup() %>%
  mutate(
    group = fct_relevel(group, "NMS"),
    parsing = fct_relevel(parsing, "parsed")
  )

# The AIC values from the full dictionary for each group are preserved from Figure 3

# Get the AIC values for the full morph set for each group with different parsing, in a fixed-effects setting
fullMorphParsedAICNMS <- clm(
  enteredResponse ~ c.(scoreMorphShortVType) + macron,
  data=dataExp2[dataExp2$group=="NMS",]
) %>%
  AIC(.)
fullMorphParsedAICMS <- clm(
  enteredResponse ~ c.(scoreMorphShortVType) + macron,
  data=dataExp2[dataExp2$group=="MS",]
) %>%
  AIC(.)
fullMorphUnparsedAICNMS <- clm(
  enteredResponse ~ c.(score) + macron,
  data=add_scores(dataExp2[dataExp2$group=="NMS",], "probs/morphs-shortvowels_types_unparsed.csv", "score")
) %>%
  AIC(.)
fullMorphUnparsedAICMS <- clm(
  enteredResponse ~ c.(score) + macron,
  data=add_scores(dataExp2[dataExp2$group=="MS",], "probs/morphs-shortvowels_types_unparsed.csv", "score")
) %>%
  AIC(.)

fullMorphAICsNMS <- data.frame(
  parsing = c("parsed", "unparsed"),
  aic = c(fullMorphParsedAICNMS, fullMorphUnparsedAICNMS)
)
fullMorphAICsMS <- data.frame(
  parsing = c("parsed", "unparsed"),
  aic = c(fullMorphParsedAICMS, fullMorphUnparsedAICMS)
)
remove(fullMorphParsedAICNMS, fullMorphUnparsedAICNMS, fullMorphParsedAICMS, fullMorphUnparsedAICMS)

fig4A <- ggplot(fig4dat[fig4dat$group=="NMS",], aes(x=morphsetsize, y=mean.AIC, ymin=lower.AIC, ymax=upper.AIC, color=scheme, fill=scheme)) +
  geom_errorbar(position=position_dodge(width=200), size=1) +
  geom_line(position=position_dodge(width=200), size=1, alpha=0.5) +
  geom_hline(aes(yintercept=fullDictAICNMS, linetype="All words (18.7k)"), color="gray30", size=1) +   
  geom_hline(data=fullMorphAICsNMS, aes(yintercept=aic, linetype="All morphs (3.6k)"), color="black", size=1) + 
  geom_point(shape=21, color="black", position=position_dodge(width=200), size=3) +
  facet_grid(. ~ parsing, labeller=labeller(parsing=function(s) paste("Stimuli:", s))) +
  labs(
    title="Morph-based Monte Carlo analyses with NMS participants",
    x="Morph set size", 
    y="AIC score"
  ) + 
  scale_x_continuous(
    breaks=seq(from=500, to=3500, by=500),
    labels=function(b){return(paste0(b/1000,"k"))}
    ) + 
  scale_color_discrete(name="Sampling", breaks=c("unweighted", "freq-weighted", "N-highest-freq")) +
  scale_fill_discrete(name="Sampling", breaks=c("unweighted", "freq-weighted", "N-highest-freq")) +
  scale_linetype_manual(name=NULL, values=c("All words (18.7k)"="dashed", "All morphs (3.6k)"="dotted")) +
  guides(color=guide_legend(order=1), fill=guide_legend(order=1), linetype=guide_legend(order=2)) +
  theme_bw() + 
  theme(
    plot.title=element_text(hjust=0.5, size=15),
    legend.text=element_text(size=10),
    strip.text = element_text(size=12),
    panel.grid = element_blank(),
    panel.background = element_blank()
  )

fig4B <- ggplot(fig4dat[fig4dat$group=="MS",], aes(x=morphsetsize, y=mean.AIC, ymin=lower.AIC, ymax=upper.AIC, color=scheme, fill=scheme)) +
  geom_errorbar(position=position_dodge(width=200), size=1) +
  geom_line(position=position_dodge(width=200), size=1, alpha=0.5) +
  geom_hline(aes(yintercept=fullDictAICMS, linetype="All words (18.7k)"), color="gray30", size=1) +   
  geom_hline(data=fullMorphAICsMS, aes(yintercept=aic, linetype="All morphs (3.6k)"), color="black", size=1) + 
  geom_point(shape=21, color="black", position=position_dodge(width=200), size=3) +
  facet_grid(. ~ parsing, labeller=labeller(parsing=function(s) paste("Stimuli:", s))) +
  labs(
    title="Morph-based Monte Carlo analyses with MS participants",
    x="Morph set size", 
    y="AIC score"
  ) + 
  scale_x_continuous(
    breaks=seq(from=500, to=3500, by=500),
    labels=function(b){return(paste0(b/1000,"k"))}
    ) + 
  scale_color_discrete(name="Sampling", breaks=c("unweighted", "freq-weighted", "N-highest-freq")) +
  scale_fill_discrete(name="Sampling", breaks=c("unweighted", "freq-weighted", "N-highest-freq")) +
  scale_linetype_manual(name=NULL, values=c("All words (18.7k)"="dashed", "All morphs (3.6k)"="dotted")) +
  guides(color=guide_legend(order=1), fill=guide_legend(order=1), linetype=guide_legend(order=2)) +
  theme_bw() + 
  theme(
    plot.title=element_text(hjust=0.5, size=15),
    legend.text=element_text(size=10),
    strip.text = element_text(size=12),
    panel.grid = element_blank(),
    panel.background = element_blank()
  )

ggarrange(fig4A, fig4B, ncol=1)

remove(fig4A, fig4B, fig4dat, fullDictAICMS, fullDictAICNMS, fullMorphAICsNMS, fullMorphAICsMS)
```

Figure 4: Monte Carlo analyses with 1,000 samples over 7 sizes of morph set, with different assumptions about the parsing of stimuli into morphs. The bars represent 95% bootstrap percentile intervals.

```
# Code for Table S12: Stage 6 statistical comparison of fixed morph sets for Exp2

# Get AIC from clm for different fixed morph sets and different parsing strategies
path <- "probs/NBestFixed-morph-shortvowels/"
filenames <- c(
  paste0("parsed/", list.files(paste0(path, "parsed/"))),
  paste0("unparsed/", list.files(paste0(path, "unparsed/")))
)
morphNBestFixedAICs <- data.frame(
  group = rep(c("NMS", "MS"), each=length(filenames)),
  parsing = rep(str_extract(filenames, "^.+(?=/)"), 2),
  freq = as.integer(rep(str_extract(filenames, "(?<=Freq)\\d+(?=_)"), 2)),
  N = as.integer(rep(str_extract(filenames, "(?<=_)\\d+(?=\\.csv)"), 2)),
  aic = rep(NA, length(filenames)*2)
)
for (group in c("NMS", "MS")) {
  groupdat <- dataExp2[dataExp2$group==group,]
  for (filename in filenames) {
    parsing <- str_extract(filename, "^.+(?=/)")
    freq <- str_extract(filename, "(?<=Freq)\\d+(?=_)")
    N <- str_extract(filename, "(?<=_)\\d+(?=\\.csv)")
    aic <- clm(
      enteredResponse ~ score + macron,
      data=add_scores(groupdat, paste0(path, filename), "score")
    ) %>%
      AIC(.)
    morphNBestFixedAICs[morphNBestFixedAICs$group==group & morphNBestFixedAICs$parsing==parsing & morphNBestFixedAICs$freq==freq & morphNBestFixedAICs$N==N, "aic"] <- aic
  }
}
remove(aic, freq, N, parsing, filename, groupdat, group, filenames, path)

morphNBestFixedAICs %>%
  pivot_wider(names_from=c(group, parsing), values_from=aic) %>%
  rename(
    "Min. freq" = "freq",
    "Morph set size" = "N",
    "AIC (NMS, parsed)" = "NMS_parsed",
    "AIC (NMS, unparsed)" = "NMS_unparsed",
    "AIC (MS, parsed)" = "MS_parsed",
    "AIC (MS, unparsed)" = "MS_unparsed"
  ) %>%
  display_table(digits=1, highlight=c("AIC (NMS, parsed)", "AIC (MS, unparsed)"), caption="Table S12: AIC scores for NMS and MS participants, using phonotactic scores based on fixed morph sets of all morphs with frequency greater than or equal to a given minimum, assuming that participants are or are not parsing nonword stimuli into morphs")

remove(morphNBestFixedAICs)
```

Table S12: AIC scores for NMS and MS participants, using phonotactic scores based on fixed morph sets of all morphs with frequency greater than or equal to a given minimum, assuming that participants are or are not parsing nonword stimuli into morphs

| Min. freq | Morph set size | AIC (NMS, parsed) | AIC (NMS, unparsed) | AIC (MS, parsed) | AIC (MS, unparsed) |
| --- | --- | --- | --- | --- | --- |
| 0 | 3636 | 103434.5 | 103978.9 | 36707.8 | 36531.8 |
| 1 | 1813 | 103388.6 | 104028.3 | 36725.1 | 36566.5 |
| 2 | 1629 | 103343.8 | 103972.2 | 36729.8 | 36581.9 |
| 3 | 1475 | 103379.0 | 103923.5 | 36732.5 | 36539.7 |
| 4 | 1395 | 103396.9 | 104094.0 | 36733.5 | 36563.8 |
| 5 | 1339 | 103352.4 | 104021.7 | 36720.3 | 36546.6 |
| 6 | 1276 | 103442.3 | 104112.9 | 36726.6 | 36554.3 |

### 3.3.7 Stage 7: What is the best source of NMS phonotactics?

In the seventh and final stage of the analysis, we compare the final models from previous stages, together with new models representing intermediate steps, to identify once and for all the best phonotactic scores for predicting NMS’ ratings. This comparison was not possible previously because some models were over all participants while others were just over NMS, and some models used fixed-effects only while others used mixed-effects.

We compare mixed-effects ordinal regression models over NMS only, which use six different phonotactic scores (all collapsing the distinction between long and short vowels):

- **Word types**: phonotactic scores are based on all 18,703 word types in the dictionary, as in stage two (but restricted to NMS only).
- **Best subset of word types**: phonotactic scores are based on the 3,164 word types with highest raw frequency in corpora, as in stage three (but in a mixed-effects setting).
- **Morph types**: phonotactic scores are based on the 3,636 morph types derived from all words in the dictionary, and are calculated under the assumption that stimuli are parsed into morphs, as in stage five (but restricted to NMS only).
- **Best subset of morph types**: phonotactic scores are based on the 1,629 morph types with highest raw frequency in corpora, and are calculated under the assumption that stimuli are parsed into morphs, as in stage six (but in a mixed-effects setting).
- **Morph types based on best subset of word types**: phonotactic scores are based on the 1,303 morph types derived from the 3,164 highest-frequency words in the dictionary, and are calculated under the assumption that stimuli are parsed into morphs.
  - This represents an intermediate step between the best subset of word types and the best subset of morph types, to check whether differences between them are due to different assumptions about the underlying unit (word vs. morph) or differences in the range of words that each corresponds to.
  - The underlying assumption here is that participants know morphs, not words. General knowledge (of words and of phonotactics) is built from knowledge of morphs.
- **Weighted morph types based on best subset of word types**: phonotactic scores are based on the 1,303 morph types derived from the 3,164 highest-frequency words in the dictionary, weighted by the number of those words they occur in, and are calculated under the assumption that stimuli are parsed into morphs.
  - This represents an alternative intermediate step between the best subset of word types and the best subset of morph types, to check whether differences between them are due to different assumptions about the underlying unit (word vs. morph) or differences in the number of times that each morph is effectively counted.
  - The underlying assumption here is that participants know words, but those words are decomposed into morphs for the purposes of generalization to unknown words.

The results of the comparison are presented in Table S13. The lowest AIC comes from the use of phonotactic scores based on the best subset of morph types.

```
# Code for Table S13: Stage 7 statistical comparison of NMS phonotactic sources for Exp2

# modelDictShortVTypeNMS <- clmm(
#   enteredResponse ~ 
#     c.(scoreDictShortVType) + macron + 
#     (1 + c.(scoreDictShortVType) + macron | workerId) + (1 | word), 
#   data=dataExp2[dataExp2$group=="NMS",]
# )
# saveRDS(modelDictShortVTypeNMS, file = "dumps/clmm/modelDictShortVTypeNMS.rds")
modelDictShortVTypeNMS <- readRDS("dumps/clmm/modelDictShortVTypeNMS.rds")

# The model based on the best subset of word types is modelNBestFixedNMS from Stage 4

# modelMorphShortVTypeNMS <- clmm(
#   enteredResponse ~ 
#     c.(scoreMorphShortVType) + macron + 
#     (1 + c.(scoreMorphShortVType) + macron | workerId) + (1 | word), 
#   data=dataExp2[dataExp2$group=="NMS",]
# )
# saveRDS(modelMorphShortVTypeNMS, file = "dumps/clmm/modelMorphShortVTypeNMS.rds")
modelMorphShortVTypeNMS <- readRDS("dumps/clmm/modelMorphShortVTypeNMS.rds")

# modelMorphNBestFixedNMS <- clmm(
#   enteredResponse ~ 
#     c.(scoreMorphNBestFixed) + macron + 
#     (1 + c.(scoreMorphNBestFixed) + macron | workerId) + (1 | word), 
#   data=dataExp2[dataExp2$group=="NMS",]
# )
# saveRDS(modelMorphNBestFixedNMS, file = "dumps/clmm/modelMorphNBestFixedNMS.rds")
modelMorphNBestFixedNMS <- readRDS("dumps/clmm/modelMorphNBestFixedNMS.rds")

# modelMorphNBestWordNMS <- clmm(
#   enteredResponse ~ 
#     c.(scoreMorphNBestWord) + macron + 
#     (1 + c.(scoreMorphNBestWord) + macron | workerId) + (1 | word), 
#   data=add_scores(dataExp2[dataExp2$group=="NMS",], "probs/morphs-shortvowels_types-NBestWords-unweighted_parsed.csv", "scoreMorphNBestWord")
# )
# saveRDS(modelMorphNBestWordNMS, file = "dumps/clmm/modelMorphNBestWordNMS.rds")
modelMorphNBestWordNMS <- readRDS("dumps/clmm/modelMorphNBestWordNMS.rds")

# modelMorphShortVWordNMS <- clmm(
#   enteredResponse ~ 
#     c.(scoreMorphShortVWord) + macron + 
#     (1 + c.(scoreMorphShortVWord) + macron | workerId) + (1 | word), 
#   data=add_scores(dataExp2[dataExp2$group=="NMS",], "probs/morphs-shortvowels_types-NBestWords-weighted_parsed.csv", "scoreMorphShortVWord"))
# saveRDS(modelMorphShortVWordNMS, file = "dumps/clmm/modelMorphShortVWordNMS.rds")
modelMorphShortVWordNMS <- readRDS("dumps/clmm/modelMorphShortVWordNMS.rds")

tibble(
  "Source of phonotactics"=c("All word types",
                             "Best subset words",
                             "All morph types",
                             "Best subset morphs",
                             "Morphs from word subset",
                             "Morphs from word subset (weighted)"),
  "AIC" = c(AIC(modelDictShortVTypeNMS),
            AIC(modelNBestFixedNMS),
            AIC(modelMorphShortVTypeNMS),
            AIC(modelMorphNBestFixedNMS),
            AIC(modelMorphNBestWordNMS),
            AIC(modelMorphShortVWordNMS))
  ) %>%
  display_table(digits=1, highlight=c("AIC"), caption="Table S13: Stage 7 comparison of AIC using best-performing phonotactic scores from all stages, applied to NMS only")

remove(modelDictShortVTypeNMS, modelNBestFixedNMS, modelMorphShortVTypeNMS, modelMorphNBestFixedNMS, modelMorphNBestWordNMS, modelMorphShortVWordNMS)
```

Table S13: Stage 7 comparison of AIC using best-performing phonotactic scores from all stages, applied to NMS only

| Source of phonotactics | AIC |
| --- | --- |
| All word types | 94021.7 |
| Best subset words | 93999.2 |
| All morph types | 93747.7 |
| Best subset morphs | 93695.6 |
| Morphs from word subset | 93824.0 |
| Morphs from word subset (weighted) | 93829.1 |

### 3.3.8 Self-reported exposure to Māori

We add NMS’ self-reported exposure to Māori as an additional predictor to the best ordinal mixed-effects model from stage seven, interacting with the other predictors. For illustrative purposes, we leave the nonsignificant interactions intact in the model (the results remain the same if they are omitted).

The results of the model are shown in Table S14. NMS’ well-formedness ratings increase significantly with their self-reported Māori exposure. Numerically, NMS with more exposure to Māori show more sensitivity to phonotactics and less sensitivity to the presence of a macron, as we might expect if their (implicit) knowledge of Māori is greater in some way. However, the interactions between Māori exposure and phonotactic score or macron presence are not significant. In other words, while NMS with high Māori exposure tend to rate items higher than NMS with low Māori exposure, they do not show any significant differences in sensitivity to phonotactic score or macron presence, suggesting that any differences in phonotactic knowledge are limited.

We plot the partial effects corresponding to the interactions of Māori exposure with macron presence and phonotactic score in Figure S8, comparing the estimates for NMS with the lower-quartile Māori exposure (score of 5) with those for NMS with the upper-quartile Māori exposure (score of 8).

```
# Code for Table S14: consideration of self-reported Māori exposure

# modelExpoNMS <- clmm(
#   enteredResponse ~
#     c.(maoriExpo) * (c.(scoreMorphNBestFixed) + macron) +
#     (1 + c.(scoreMorphNBestFixed) + macron | workerId) + (1 + c.(maoriExpo) | word),
#   data=dataExp2[dataExp2$group=="NMS",]
# )
# saveRDS(modelExpoNMS, file = "dumps/clmm/modelExpoNMS.rds")
modelExpoNMS <- readRDS("dumps/clmm/modelExpoNMS.rds")

clm_table(modelExpoNMS, digits=3, caption="Table S14: Ordinal mixed-effects model of well-formedness ratings including NMS' self-reported exposure to Māori")
```

Table S14: Ordinal mixed-effects model of well-formedness ratings including NMS’ self-reported exposure to Māori

|  | Parameter | Estimate | Std. Error | \(z\) | \(p\) |  |
| --- | --- | --- | --- | --- | --- | --- |
| Effects | maoriExpo (centered) | 0.149 | 0.049 | 3.054 | 0.002 | \*\* |
|  | scoreMorphNBestFixed (centered) | 4.962 | 0.299 | 16.612 | <0.001 | \*\*\* |
|  | macron = TRUE | 0.276 | 0.104 | 2.652 | 0.008 | \*\* |
|  | maoriExpo (centered) × scoreMorphNBestFixed (centered) | 0.219 | 0.125 | 1.756 | 0.079 | . |
|  | maoriExpo (centered) × macron = TRUE | -0.074 | 0.038 | -1.915 | 0.055 | . |
| Thresholds | 1|2 | -1.548 | 0.121 |  |  |  |
|  | 2|3 | 0.035 | 0.121 |  |  |  |
|  | 3|4 | 1.218 | 0.121 |  |  |  |
|  | 4|5 | 2.629 | 0.122 |  |  |  |

```
# Code for Figure S8: plot of partial effects of model considering self-reported Māori exposure

figS8a.mean <- clm_plotdat(modelExpoNMS, c("maoriExpo", "scoreMorphNBestFixed"), xlevels=list(scoreMorphNBestFixed=25, maoriExpo=c(5,8)), type="mean") %>%
  mutate(
    maoriExpo = factor(maoriExpo, labels=c("LQ (5)", "UQ (8)")) %>% fct_relevel("UQ (8)")
  ) %>%
  ggplot(., aes(x=scoreMorphNBestFixed, y=pred, color=maoriExpo, fill=maoriExpo)) +
    geom_ribbon(aes(ymin=lci, ymax=uci), alpha=0.3, color=NA) +
    geom_line(size=1) +
    xlab("Phonotactic score") +
    ylab("Predicted mean rating") + 
    scale_x_continuous(n.breaks=4) +
    scale_color_discrete(name="Māori\nexposure") +
    scale_fill_discrete(name="Māori\nexposure") +
    theme_classic() + 
    theme(
      legend.position="none",
      panel.background = element_blank()
    )

figS8a.dist <- clm_plotdat(modelExpoNMS, c("maoriExpo", "scoreMorphNBestFixed"), xlevels=list(scoreMorphNBestFixed=25, maoriExpo=c(5,8)), type="dist") %>%
  mutate(
    maoriExpo = factor(maoriExpo, labels=c("LQ (5)", "UQ (8)")) %>% fct_relevel("UQ (8)")
  ) %>%
  ggplot(., aes(x=scoreMorphNBestFixed, y=pred, color=maoriExpo, fill=maoriExpo)) +
    geom_ribbon(aes(ymin=lci, ymax=uci), alpha=0.3, color=NA) +
    geom_line(size=1) +
    xlab("Phonotactic score") +
    ylab("Probability of rating") + 
    scale_x_continuous(n.breaks=4) +
    scale_color_discrete(name="Māori\nexposure") +
    scale_fill_discrete(name="Māori\nexposure") +
    facet_grid(. ~ response, labeller=as_labeller(function(x) str_c("Rating: ", x))) +
    ylim(0, 1) +  
    theme_bw() + 
    theme(
      legend.position="right",
      panel.background = element_blank()
    )

figS8b.mean <- clm_plotdat(modelExpoNMS, c("maoriExpo", "macron"), xlevels=list(maoriExpo=c(5,8)), type="mean") %>%
  mutate(
    maoriExpo = factor(maoriExpo, labels=c("LQ (5)", "UQ (8)")) %>% fct_relevel("UQ (8)"),
    macron.int = as.integer(macron),
    macron = fct_recode(macron, "Yes"="TRUE", "No"="FALSE")
  ) %>%
  ggplot(., aes(x=macron, y=pred, color=maoriExpo, shape=maoriExpo)) +
    geom_point(size=4, position=position_dodge(width=0.2)) +
    geom_line(aes(x=as.integer(macron)), size=1, alpha=0.4, position=position_dodge(width=0.2)) +
    geom_errorbar(aes(ymin=lci, ymax=uci), size=1, width=0.3, position=position_dodge(width=0.2)) +
    xlab("Presence of macron") +
    ylab("Predicted mean rating") + 
    scale_color_discrete(name="Māori\nexposure") +
    scale_shape_discrete(name="Māori\nexposure") +  
    theme_classic() + 
    theme(
      legend.position="none",
      panel.background = element_blank()
    )

figS8b.dist <- clm_plotdat(modelExpoNMS, c("maoriExpo", "macron"), xlevels=list(maoriExpo=c(5,8)), type="dist") %>%
  mutate(
    maoriExpo = factor(maoriExpo, labels=c("LQ (5)", "UQ (8)")) %>% fct_relevel("UQ (8)"),
    macron.int = as.integer(macron),
    macron = fct_recode(macron, "Yes"="TRUE", "No"="FALSE")
  ) %>%
  ggplot(., aes(x=macron, y=pred, color=maoriExpo, shape=maoriExpo)) +
    geom_point(size=2, position=position_dodge(width=0.2)) +
    geom_line(aes(x=as.integer(macron)), size=1, alpha=0.7, position=position_dodge(width=0.2)) +
    geom_errorbar(aes(ymin=lci, ymax=uci), size=1, width=0.3, position=position_dodge(width=0.2)) +
    xlab("Presence of macron") +
    ylab("Probability of rating") + 
    scale_color_discrete(name="Māori\nexposure") +
    scale_shape_discrete(name="Māori\nexposure") +  
    facet_grid(. ~ response, labeller=as_labeller(function(x) str_c("Rating: ", x))) +
    ylim(0, 1) +  
    theme_bw() + 
    theme(
      legend.position="right",
      panel.background = element_blank()
    )

ggarrange(figS8a.mean, figS8a.dist, figS8b.mean, figS8b.dist, ncol=2, labels=c("a.", "", "b.", ""), widths=c(1,5))

remove(figS8a.mean, figS8a.dist, figS8b.mean, figS8b.dist, modelExpoNMS)
```

Figure S8: Partial effect plots for interactions with self-reported Māori exposure in ordinal mixed-effects model of NMS well-formedness ratings. Left-hand plots show predicted mean ratings; right-hand plots show predicted distributions over ratings (facets). Figure S8a (top) shows the interaction between Māori exposure and phonotactic score; Figure S8b (bottom) shows the interaction between Māori exposure and presence of macrons. Error bars represent 95% confidence intervals.
